# Total Phenylethanoid Glycosides from *Cistanche tubulosa* Alleviate 5-Fluorouracil-Induced Fatigue-Like Behavior and Skeletal Muscle Injury in Mice

**DOI:** 10.3390/biomedicines14102236

**Published:** 2026-10-02

**Authors:** Jinjin Chen, Jianhua Yang, Yexia Cao, Limei Wen, Xufeng Su, Jiayuan Yan, Qiang Hou, Shilei Zhang, Junping Hu

**Affiliations:** 1 School of Pharmacy, Xinjiang Medical University, Urumqi 830017, China; 13325648820@163.com (J.C.); yjh_yfy@163.com (J.Y.); 15199683978@163.com (Y.C.); wendywenlimei@163.com (L.W.); hq_sci@163.com (Q.H.); 2 School of Public Health, Xinjiang Medical University, Urumqi 830011, China; 18160517480@163.com (X.S.); young_jiayuan@163.com (J.Y.)

**Keywords:** chemotherapy-induced fatigue-like behavior, *Cistanche tubulosa*, phenylethanoid glycosides, skeletal muscle injury

## Abstract

**Objectives:** This study investigated the effects of total phenylethanoid glycosides from *Cistanche tubulosa* (CPhGs) on 5-fluorouracil (5-FU)-induced fatigue-like behavior and tissue injury in mice and examined associated molecular changes. **Methods:** Public transcriptomic datasets (GSE80473, GSE121972, and GSE77745) were analyzed to provide exploratory pathway-level context for hypothesis-driven markers associated with chemotherapy- or cachexia-related skeletal muscle injury. Forty tumor-free BALB/c mice were randomly assigned to a normal control group or treated with 5-FU (20 mg/kg, intraperitoneally) for five consecutive days. The 5-FU-treated mice were subsequently divided into model, low-dose-CPhG (250 mg/kg), and high-dose-CPhG (500 mg/kg) groups and treated orally for 14 days. Exercise endurance, forelimb grip strength, serum markers of tissue injury and inflammation, and histopathological changes in skeletal muscle, epididymal adipose tissue, and liver were evaluated. Proteins involved in Akt/FoxO3a signaling, muscle protein degradation, and mitochondrial metabolism were measured in skeletal muscle. Molecular docking was used to assess the predicted interactions of echinacoside and acteoside with selected proteins. **Results:** Exploratory transcriptomic analysis suggested alterations in muscle contraction, fatty acid oxidation and energy metabolism, with external cachexia data providing context for FoxO/ubiquitin–proteasome signaling and inflammation in skeletal muscle injury. Compared with control mice, 5-FU-treated mice showed body weight loss, reduced swimming endurance, decreased forelimb grip strength, elevated serum CK, CK-MB, LDH, LDH1, TNF-α, and IL-6 levels, and pathological changes in skeletal muscle, adipose tissue, and liver. CPhGs attenuated body weight loss, prolonged swimming time, improved forelimb grip strength, reduced serum markers of tissue injury and inflammation, and improved tissue morphology. CPhGs were also associated with lower skeletal muscle MuRF1 and Atrogin-1 expression and higher p-Akt/Akt and p-FoxO3a/FoxO3a ratios and PGC-1α expression. These effects were generally more pronounced at the higher dose. Docking analysis generated exploratory poses of echinacoside and acteoside with AKT1, MuRF1, and IL-6; the docking scores do not establish binding affinity or target engagement. **Conclusions:** CPhGs alleviated 5-FU-induced fatigue-like behavior and associated skeletal muscle, adipose tissue, and hepatic abnormalities in mice. These effects were associated with regulation of Akt/FoxO3a signaling, lower expression of proteolysis-associated proteins, higher PGC-1α expression, and reduced systemic inflammation. The in vivo findings concern the complete CPhG preparation; the contributions and direct molecular targets of echinacoside and acteoside require experimental confirmation.

## 1. Introduction

Cancer-related fatigue (CRF) ranks among the most prevalent complications in malignancy, with prevalence varying by disease and treatment setting [1]. Unlike physiological fatigue, CRF is characterized by prolonged duration, severe intensity, and resistance to rest, substantially impairing quality of life and compliance with anticancer therapy [2]. Although the pathogenesis of CRF remains incompletely understood, chronic inflammatory activation and metabolic dysregulation may contribute to fatigue; skeletal muscle wasting is particularly relevant to cancer cachexia, a distinct but potentially coexisting syndrome [1,3].

Glucose–lipid metabolic dysfunction is a feature of cancer cachexia that may also contribute to fatigue. Aberrant tumor proliferation and sustained release of inflammatory cytokines (e.g., TNF-α, IL-6) can alter energy-substrate availability and peripheral metabolism [4,5]. Additionally, persistent inflammation and altered lipid handling can contribute to adipose tissue dysfunction and disturbed energy balance, providing a pathological context for skeletal muscle wasting [6,7]. These tumor-associated processes are relevant background but are not reproduced fully by chemotherapy exposure in tumor-free mice.

Excessive activation of the ubiquitin-proteasome system (UPS) is an important mechanism of skeletal muscle atrophy. Marked upregulation of the E3 ubiquitin ligases MuRF1 and Atrogin-1 mediates myofibrillar protein degradation, culminating in progressive loss of muscle mass [8,9]. Concurrently, sustained activation of NF-κB inflammatory signaling promotes transcription of these atrophy-related genes, linking inflammation with muscle wasting [10,11].

Current clinical management of CRF includes exercise and psychosocial interventions, while evidence for pharmacological approaches remains limited [12]. *Cistanche tubulosa* (Schenk) Wight, a traditional tonic herb, contains total phenylethanoid glycosides (CPhGs; primarily echinacoside and acteoside) as major bioactive constituents, exhibiting multifaceted pharmacological activities including anti-inflammatory, antioxidant, anti-fatigue, and glucose-lipid regulatory effects [13,14]. Our previous work investigated isolated acteoside from *C. tubulosa* and reported effects on PHD2-related skeletal muscle mitophagy in a fatigue model [15]. That study does not establish the same mechanism for the complete CPhG mixture. However, the effects of CPhGs on atrophy-associated alterations and metabolic tissue injury during chemotherapy require further investigation. Therefore, this study investigated the effects of CPhGs on skeletal muscle, adipose tissue, and hepatic abnormalities in mice with 5-FU-induced fatigue-like behavior and systemic toxicity, using animal experiments alongside exploratory GEO analysis and molecular docking.

To systematically address this question, we incorporated public transcriptomic data as an initial approach. Such datasets provide external disease or drug-injury context for hypothesis-driven experimental readouts. Accordingly, this study first utilized GEO transcriptomic data to explore potential pathways involved in skeletal muscle injury under FOLFIRI combination chemotherapy and cancer cachexia contexts, establishing a rationale for selecting detection indices including Akt/FoxO3a, MuRF1, Atrogin-1, and PGC-1α. Building on this foundation, we established a tumor-free mouse model of 5-FU-induced fatigue-like behavior and systemic toxicity and systematically evaluated the effects of CPhGs on metabolic tissue abnormalities and atrophy-associated alterations through behavioral assessments, serum biochemistry, multi-organ histopathology, and detection of skeletal muscle Akt/FoxO3a signaling and UPS key protein expression. Furthermore, we employed molecular docking to simulate and analyze the spatial binding conformations and interactions (including hydrogen bonding and hydrophobic forces) between major characterized constituents (echinacoside and acteoside) and key target proteins (AKT1, MuRF1, and IL-6), generating hypotheses for subsequent binding studies. These complementary analyses evaluate the CPhG mixture in a chemotherapy-toxicity model; they do not constitute an unbiased target-discovery or mechanistic-validation pipeline.

## 2. Materials and Methods

### 2.1. Materials

#### 2.1.1. Animals

Forty SPF-grade male BALB/c mice, 8 weeks old, weighing 22 ± 3.0 g, were purchased from and housed at the Experimental Animal Center of Xinjiang Medical University [Animal Production License: SCXK (Xin) 2023-0001; Animal Use License: SYXK (Xin) 2023-0004]. All mice underwent a 7-day acclimatization period in a standardized environment-controlled facility before experimental procedures, maintained at 22 ± 2 °C with 50–70% relative humidity and a 12 h/12 h light–dark cycle. This study was approved by the Animal Ethics Committee of Xinjiang Medical University (ethics approval number: IACUC-20250425-52). The sample size (*n* = 10/group; total *N* = 40) was based on previous comparable models, feasibility, and animal welfare rather than a formal a priori power calculation. Group allocation used a random number table. Allocation was concealed using numbered cages prepared by an independent investigator. Behavioral testing, histological evaluation, and data collection and analysis were performed with investigators blinded to group allocation.

#### 2.1.2. Drugs and Reagents

Total phenylethanoid glycosides from *Cistanche tubulosa* (CPhGs; >80%, containing 44.5% echinacoside and 16.1% acteoside) were obtained from Hetian Dichen Biotechnology Co., Ltd. (Xinjiang, China). 5-Fluorouracil (5-FU, batch number 250904) was obtained from Southwest Pharmaceutical Co., Ltd. (Chongqing, China).TNF-α and IL-6 ELISA kits (catalog numbers GEM0004 and GEM0001) were obtained from Wuhan Servicebio Technology Co., Ltd. (Wuhan, China). An H&E staining kit, Masson’s trichrome staining kit, PAS staining kit, and Oil Red O staining solution (catalog numbers G1003, G1006, G1008, and G1015) were acquired from Wuhan Servicebio Technology Co., Ltd. MuRF1, Atrogin-1, and GAPDH antibodies (catalog numbers 55456-1-AP, 67172-1-Ig, and 60004-1-Ig) were acquired from Wuhan Sanying Biotechnology Co., Ltd. (Wuhan, China). Akt antibody (catalog number 9272S) was obtained from Cell Signaling Technology (CST). (Danvers, MA, USA). P-Akt, FoxO3a, p-FoxO3a, and PGC-1α antibodies (catalog numbers YM8304, YT1763, YM8032, and YM0519) were obtained from Suzhou Riying Biotechnology Co., Ltd. (Suzhou, China). Eco-friendly GD muscle fixative and adipose tissue-specific fixative (catalog numbers G1111 and G1119) were acquired from Wuhan Servicebio Technology Co., Ltd. HRP-conjugated goat anti-rabbit IgG and HRP-conjugated goat anti-mouse IgG secondary antibodies (catalog numbers SA00001-2 and SA00001-1-A, respectively) were obtained from Wuhan Sanying Biotechnology Co., Ltd. Adiponectin antibody (catalog number GB115581-100) was acquired from Wuhan Servicebio Technology Co., Ltd.

The *C. tubulosa* material was collected in Xinjiang in October 2018, authenticated by Prof. Junping Hu, and deposited in the Herbarium of the School of Pharmacy, Xinjiang Medical University (voucher No. 201810). This number identifies the botanical voucher, not an extract manufacturing batch or date. Powdered plant material was stored in sealed, light-protected containers under dry conditions at 4 °C until use. 

The preparation had previously been characterized in our laboratory by HPLC using an Agilent C18 column (4.6 × 250 mm, 5 μm), an acetonitrile/0.1% phosphoric acid gradient, a flow rate of 1.0 mL/min, a column temperature of 35 °C, and detection at 254 nm, with echinacoside and acteoside as reference compounds. The two quantified constituents together accounted for 60.6% of the preparation; the remaining 39.4% was not comprehensively characterized at the individual-compound level. Related CPhGs and *C. tubulosa* preparations have been studied by Wang et al. [16] and Liao et al. [17].

#### 2.1.3. Instruments

We utilized a Heraeus Fresco 17 centrifuge and FC automated microplate reader (Thermo Fisher Scientific, Waltham, MA, USA); a GL-88B vortex mixer (Haimen Qilinbeier Instrument Manufacturing Co., Ltd., Haimen, China); a CRYOSTAR NX50 cryostat (Thermo Fisher Scientific, Waltham, MA, USA); a NIKON ECLIPSE E100 upright optical microscope (Nikon Corporation, Tokyo, Japan); and a ZR-07 grip strength meter for mice and rats (Shanghai Puxin Instrument Technology Co., Ltd., Shanghai, China).

### 2.2. GEO Datasets

#### 2.2.1. GEO Dataset Retrieval and Selection

To explore aberrant pathways in chemotherapy-related skeletal muscle injury at the public transcriptomic level and provide a rationale for selecting protein detection indices in subsequent animal experiments, we searched the Gene Expression Omnibus (GEO) database for mouse skeletal muscle expression profiling datasets related to chemotherapy, skeletal muscle injury, or cancer cachexia. GSE80473 was selected as the primary analysis dataset, comprising RNA-seq data from FOLFIRI-treated normal mouse quadriceps femoris, divided into the Vehicle control group (*n* = 4) and the FOLFIRI treatment group (*n* = 4). Since the FOLFIRI regimen includes 5-FU, leucovorin, and CPT-11, this dataset was used to analyze transcriptomic alterations in chemotherapy-related skeletal muscle injury. Two additional datasets were included as external contextual references: GSE121972 represented C26 cancer cachexia, comparing expression differences between control and tumor-bearing groups in extensor digitorum longus (EDL) and soleus (SOL) muscles (6 control and 6 tumor samples for each muscle); GSE77745 served as an auxiliary external drug-context dataset for chemotherapy-induced myotoxicity, comparing control (PBS; *n* = 5) and doxorubicin-treated (*n* = 5) tibialis anterior samples. External contextual datasets were used solely to assess whether relevant pathways exhibited similar abnormalities in other skeletal muscle wasting contexts and were not merged with the primary dataset for analysis.

#### 2.2.2. Data Preprocessing and Differential Expression Analysis

For GSE80473, we downloaded the supplementary count matrix and the author-provided edgeR statistical results table from GEO. Local edgeR re-analysis was performed on the count matrix: low-expression genes were filtered, TMM normalization was applied, and quasi-likelihood F-tests were used to compare differential expression between FOLFIRI and Vehicle groups. GSE121972 and GSE77745, both microarray datasets, were processed using GEOquery to obtain expression matrices and platform annotations. Probes lacking gene symbol annotations were removed. For probes annotated with multiple gene symbols, the first symbol was retained; when multiple probes corresponded to the same gene, the probe with the highest overall variance was retained. Based on expression value distributions, log2 transformation was applied as needed, followed by differential expression analysis using limma. The threshold for differential gene identification was uniformly set at FDR < 0.05 and |log2FC| > 1.

#### 2.2.3. Quality Control, Enrichment Analysis, and Pathway Focus

Quality control was performed for each dataset using expression distribution boxplots, principal component analysis (PCA), and sample correlation heatmaps. Differential expression results were visualized with volcano plots, and heatmaps of top genes from the primary dataset were generated. GO and KEGG overrepresentation analyses were restricted to datasets with qualifying differentially expressed genes, with background genes defined as all genes successfully annotated and included in the differential analysis. Gene Set Enrichment Analysis (GSEA) was performed using full gene ranking: for edgeR results, the ranking statistic was sign(logFC) × −log10(*p* value); for limma results, the t-statistic was used. Analysis focused on pathways related to muscle contraction, fatty acid oxidation, mitochondrial energy metabolism, FoxO signaling, the ubiquitin–proteasome system, inflammatory response, and extracellular matrix remodeling. GO Biological Process gene sets were constructed using GO.db 3.22.0 and org. Mm.eg.db 3.22.0. GSEA used fgseaSimple with 10,000 permutations, minimum and maximum gene-set sizes of 10 and 500, respectively, and random seed 20260702. Benjamini–Hochberg correction was applied; FDR < 0.05 defined statistical significance. Signals not meeting this threshold were treated as exploratory.

### 2.3. Molecular Docking

To explore possible interactions between major characterized constituents (acteoside and echinacoside) and selected proteins (IL-6, MuRF1, and AKT1), blind docking was performed. Acteoside (PubChem CID 5281800) and echinacoside (CID 5281771) were obtained from PubChem. Receptor structures were human IL-6 (PDB 1ALU, chain A, 1.90 Å), the MuRF1 B-box2 domain (PDB 3DDT, chains A–C, 1.90 Å), and AKT1 (PDB 6HHG, chain A, 2.30 Å). Receptor and ligand preparation used the automated CB-Dock2 workflow, including hydrogen addition, ligand preparation, receptor cleaning, and removal of co-crystallized waters and hetero groups. Alternative protonation and tautomeric states were not manually enumerated. CB-Dock2 uses AutoDock Vina v1.1.2 with automatic cavity identification and docking-box placement. The pose with the lowest Vina score was selected for visualization using the built-in visualization function of the CB-Dock2 web server.

### 2.4. In Vivo Experiments

#### 2.4.1. Model Establishment, Grouping, and Drug Administration

Thirty 8-week-old BALB/c mice were randomly selected and received intraperitoneal injections of 5-FU (20 mg/kg) for 5 consecutive days, while the remaining 10 mice (normal group) received equivalent volumes of saline. The 30 mice were randomly divided into a model group, a low-dose-CPhG group (250 mg/kg), and a high-dose-CPhG group (500 mg/kg), with 10 mice per group. Following model establishment, treatment was administered by gavage for 14 consecutive days, while the normal and model groups received saline. Body weight, mental status, activity levels, and fur appearance were monitored throughout the experiment. General conditions were summarized weekly and reported descriptively without a formal scoring system. Food intake was monitored throughout the experiment. After the final administration, mice were deprived of food and water overnight and anesthetized the following morning with intraperitoneal sodium pentobarbital (50 mg/kg). Blood was collected via orbital puncture, and liver, adipose tissue, and skeletal muscle were collected during the same morning period. Terminal body weight (g) was recorded.

CPhGs were administered by gavage in the morning, and behavioral assessments were performed in the afternoon; all groups followed the same schedule. 5-FU injections were restricted to the induction phase. The doses were informed by previous preclinical studies of Cistanche-derived phenylethanoid glycosides, including a phenylethanoid-rich C. deserticola extract tested at comparable doses [18]. Differences in species and preparation preclude assuming equivalent potency or safety. Humane endpoints were predefined as >20% baseline body-weight loss, inability to eat or drink independently, persistent hunched posture, marked inactivity with reduced responsiveness, severe dehydration/diarrhea/infection, or a moribund state. No spontaneous deaths, humane-endpoint euthanasia, or exclusions were reported. 

#### 2.4.2. General Condition Observation, Body Weight, Exhaustive Swimming Time Measurement, and Forelimb Grip Strength Measurement

During the intervention period, mental status and spontaneous activity were recorded daily, and body weight was measured on days 1, 6, 13, and 20. Exhaustive swimming time was recorded on days 1, 6, 13, and 20. Forelimb grip strength was also measured on days 1, 6, 13, and 20, and the results were recorded in Newtons (N). The rectangular swimming container measured approximately 40 × 20 × 30 cm (length × width × height). Water was maintained within 25–30 °C using the same preparation procedure for all groups; a fixed temperature was not continuously recorded. Mice underwent 3 days of unloaded adaptive training (15 min) before formal testing. During testing, mice carried a lead wire weighing 8% of their body weight. Exhaustion was defined as head submersion >10 cm for ≥10 s. General-condition observations were qualitative and were not subjected to statistical analysis.

#### 2.4.3. Biochemical Index Detection

Collected blood samples were allowed to stand for 2 h, then centrifuged at 4 °C and 4000 rpm for 15 min to collect serum. Following the instructions of biochemical kits, serum levels of TNF-α, IL-6, CK, CK-MB, LDH, and LDH1 were measured to assess inflammatory status and non-specific tissue injury.

#### 2.4.4. Skeletal Muscle Histological Staining

Transverse samples from the mid-portion of the gastrocnemius muscle from each group were fixed in 4% paraformaldehyde and subjected to sequential gradient ethanol dehydration, xylene clearing, and paraffin embedding to prepare 5 μm thick sections. Periodic acid–Schiff (PAS) and Masson’s trichrome staining were performed. Histopathological examination was conducted under light microscopy, and PAS-positive area and collagen-positive area (collagen volume fraction, CVF) were quantified as percentages of the analyzed tissue area using Image J 1.54f. For each parameter, one section and one randomly selected field at 20× magnification were analyzed per mouse (*n* = 10/group). Each data point represents one animal. Image acquisition settings were identical across groups. Randomly coded slides were assessed by blinded investigators; image acquisition and analysis were performed by different individuals. Background-based thresholds were predefined by a blinded analyst and applied uniformly across groups. The same sampling and thresholding principles were used for adipose and liver analyses. PAS-positive area was interpreted as carbohydrate-rich staining, not a direct biochemical measurement of glycogen. 

#### 2.4.5. Epididymal Adipose Tissue Histological Staining

Epididymal adipose tissues from each group were fixed in 4% paraformaldehyde and subjected to sequential gradient ethanol dehydration, xylene clearing, and paraffin embedding to prepare 5 μm thick sections. Hematoxylin–eosin (H&E) staining, Masson’s trichrome staining, and adiponectin immunohistochemical staining were performed. Histopathological examination was conducted under light microscopy, and collagen-positive area and adiponectin-positive area were quantified using ImageJ, with one section and one randomly selected 20× field per mouse (*n* = 10/group). 

#### 2.4.6. Hepatic Tissue Histological Staining

Liver samples from the same anatomical location in the left lobe were fixed in 4% paraformaldehyde. One portion underwent gradient ethanol dehydration, xylene clearing, and paraffin embedding to prepare 5 μm thick sections for PAS staining. Another portion was embedded in OCT and cryosectioned (8–10 μm) for Oil Red O staining. Staining was performed according to the respective kit instructions, and histopathological examination was conducted under light microscopy. PAS-positive and Oil Red O-positive areas were quantified using ImageJ with one section and one randomly selected 20× field per mouse (*n* = 10/group), under the blinded and uniform acquisition/thresholding procedures described above.

#### 2.4.7. Western Blot Detection of Protein Expression in Skeletal Muscle

Skeletal muscle tissues from each group were lysed with RIPA buffer to extract total protein, and protein concentration was determined by BCA assay. Protein samples were subjected to sodium dodecyl sulfate–polyacrylamide gel electrophoresis, transferred to PVDF membranes, and blocked with 5% non-fat milk solution. Diluted primary antibodies against MuRF1, Atrogin-1, PGC-1α, Akt, p-Akt, FoxO3a, p-FoxO3a, and GAPDH were applied and incubated overnight at 4 °C. HRP-conjugated IgG secondary antibody was applied and incubated for 1 h at room temperature, followed by development and exposure. Quantitative analysis was performed using Image-Pro Plus 6.0 software. GAPDH served as the internal reference, and relative protein expression was calculated as the ratio of target band to internal reference band gray values. Phosphorylation was expressed as p-Akt/Akt and p-FoxO3a/FoxO3a ratios. Three independent mice per group, each from a different cage, were randomly selected for Western blotting at the experimental-design stage before results were available; operators and analysts were blinded. The same animals and tissue lysates used for Western blot analysis were the same. Other samples were allocated to other assays rather than excluded on the basis of results. In a pilot comparison of GAPDH, β-actin, and β-tubulin, GAPDH showed the lowest variability in total-protein-normalized signal across groups, whereas β-actin varied after 5-FU exposure. 

### 2.5. Statistical Analysis

Data were processed using SPSS 27.0 and GraphPad Prism 11. Experimental data are expressed as mean ± SD. The animal was the experimental unit: *n* = 10/group for body weight, swimming endurance, forelimb grip strength, serum assays, and histology, and *n* = 3 independent mice/group for Western blotting. Body weight, swimming endurance, and forelimb grip strength were analyzed using two-way repeated-measure ANOVA with group as the between-subject factor, time as the within-subject factor, and a group × time interaction. Sphericity was not assumed, and the Geisser–Greenhouse correction was applied. Tukey-adjusted comparisons were made among groups within each time point. Partial η^2^ was reported for omnibus effects. 

Residual-versus-fitted, absolute-residual, and Q–Q plots were examined. Body-weight residuals showed mild lower-tail deviation. Swimming-endurance diagnostics showed a large negative residual and increased dispersion; the corresponding original record was checked and the observation retained as biologically valid. Thus, normality and homoscedasticity were not assumed to be fully satisfied for swimming endurance. No longitudinal observations were missing, and no data were removed solely because they were statistically extreme. Endpoint comparisons used one-way ANOVA. *p* < 0.05 was considered statistically significant; values below the software reporting limit are given as *p* < 0.0001.

## 3. Results

### 3.1. Public GEO Transcriptome Analysis Provides Exploratory Context for Skeletal Muscle Injury-Related Indices

This study employed GSE80473 as the primary analysis dataset and included GSE121972 (cancer cachexia skeletal muscle) and GSE77745 (doxorubicin chemotherapy-induced myotoxicity) as external contextual sets. By analyzing chemotherapy- and cachexia-related skeletal muscle injury models, pathway-level patterns were explored to contextualize hypothesis-driven readouts for subsequent animal experiments. Sample expression distribution, PCA, and correlation analysis was used for quality assessment; Figure 1A shows the primary-dataset PCA. Additional quality-control, differential-expression, enrichment, GSEA, and key-gene analyses for the included GEO datasets are provided in Supplementary Figures S1–S52.

**Figure 1:**
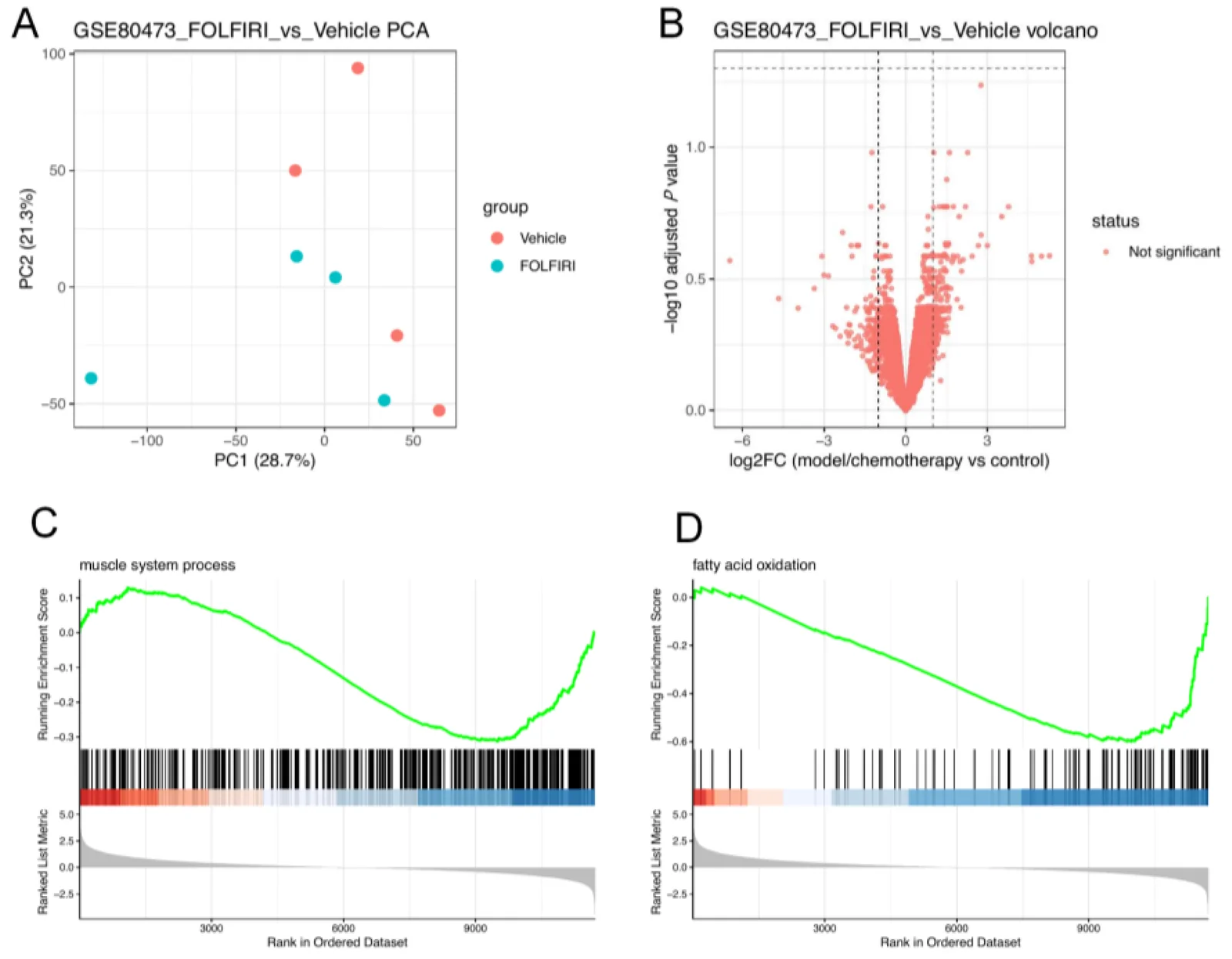
Public GEO transcriptome analysis. (**A**) Principal component analysis (PCA) of GSE80473, with Vehicle and FOLFIRI samples indicated by different colors. (**B**) Volcano plot of differential expression between FOLFIRI and Vehicle groups. Vertical dashed lines indicate the |log2FC| threshold, and the horizontal dashed line indicates the adjusted *p*-value threshold. Red points indicate genes that did not meet the predefined significance criteria. (**C**,**D**) GSEA plots for muscle system process and fatty acid oxidation, respectively. The green curve represents the running enrichment score, black vertical bars indicate the positions of genes in the ranked gene list, the red-to-blue bar represents the direction of the ranked metric, and the gray distribution represents the ranked-list metric.

Local edgeR re-analysis of the GSE80473 count matrix included 12,513 genes. Comparing the FOLFIRI group to the Vehicle group, no genes met the strict significance threshold (FDR < 0.05 and |log2FC| > 1; minimum FDR = 0.0581, Figure 1B). Given the limited sample size, gene set enrichment analysis (GSEA) using full gene ranking was performed to assess pathway-level changes. Exploratory GSEA examined GO terms related to fatty acid catabolism, fatty acid oxidation, fatty acid metabolism, precursor metabolite and energy generation, oxidation of organic compounds to produce energy, muscle system processes, muscle contraction, and striated muscle contraction in FOLFIRI-treated skeletal muscle (Figure 1C,D), providing hypotheses about skeletal muscle contractile function and energy metabolism. 

The two external contextual datasets were analyzed separately. In GSE121972, 20,353 genes were analyzed in both EDL and SOL, yielding 787 (390 upregulated, 397 downregulated) and 558 (249 upregulated, 309 downregulated) differentially expressed genes, respectively. GO and GSEA showed that EDL was enriched in muscle system processes, myocyte differentiation, striated muscle contraction, oxidation of organic compounds to produce energy, regulation of inflammatory response, and extracellular matrix organization, while SOL was enriched in mitochondrial respiratory chain complex assembly, mitochondrial translation, oxidative phosphorylation, cytokine-mediated signaling, acute inflammatory response, extracellular matrix organization, and collagen fibril organization. These exploratory results suggest alterations in muscle function, inflammation, mitochondrial energy metabolism, and ECM/collagen remodeling pathways in the context of cachexia-related skeletal muscle wasting. GSE77745 included 20,116 genes, with only one gene meeting the screening threshold, suggesting weak differential signals in this acute doxorubicin model under current thresholds; therefore, it served only as auxiliary reference and was not included in the main pathway conclusions. 

At the individual gene level, Akt1, Foxo3, Trim63, Fbxo32, Ppargc1a, Col1a1, Col3a1, Mstn, Nfkb1, Stat3, Myog, and Myod1 were all detectable in GSE80473 (Il6 and Tnf were not detected using exact gene symbols). Among these, Trim63, Fbxo32, and Ppargc1a showed downward trends or nominal *p*-value signals in the FOLFIRI group but did not pass FDR correction and thus were not interpreted as significantly differential genes. In GSE121972, Akt1, Foxo3, Trim63, Fbxo32, Ppargc1a, Mstn, Stat3, and Myog in EDL and Foxo3, Trim63, Fbxo32, Stat3, and Myog in SOL exhibited significant alterations, suggesting aberrant FoxO/UPS and inflammation-STAT-related molecules in cachexia-related skeletal muscle wasting.

In summary, GEO transcriptome analysis provides exploratory context for transcriptional changes in muscle contraction, fatty acid oxidation, and energy metabolism in chemotherapy-related skeletal muscle injury. The external cachexia dataset suggests alterations related to FoxO/UPS, inflammation/STAT, oxidative phosphorylation, and ECM/collagen remodeling. Akt/FoxO3a, MuRF1, Atrogin-1, and PGC-1α were hypothesis-driven readouts selected for their biological relevance together with this pathway-level context, not unbiased CPhG targets identified by transcriptomics.

### 3.2. Molecular Docking Analysis of Major Characterized Constituents from CPhGs with Selected Proteins

**Figure 2:**
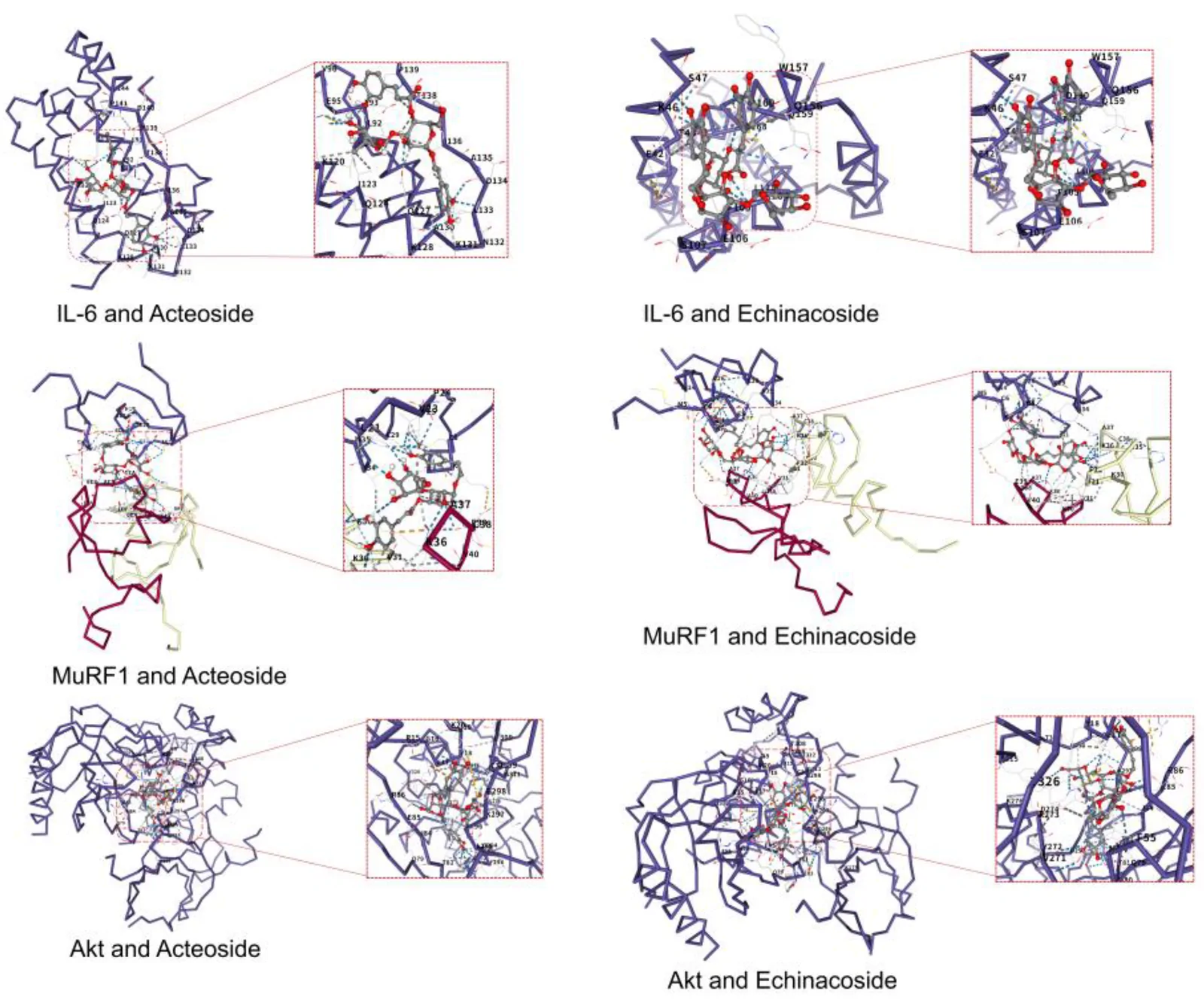
Molecular docking poses of acteoside and echinacoside with IL-6, MuRF1, and AKT1. MuRF1 docking used the B-box2 domain. Poses are exploratory predictions and do not demonstrate target engagement.

Molecular docking scores and poses are shown in Table 1 and Figure 2. Vina scores are computational scoring values and are not experimentally measured binding affinities. No fixed score threshold was used to infer strong binding or target engagement.

Acteoside yielded Vina scores of −7.7, −8.8, and −10.5 kcal/mol for IL-6, MuRF1, and AKT1, respectively; the corresponding echinacoside scores were −7.1, −7.9, and −9.9 kcal/mol. These scores describe the selected computational poses. Scores across different proteins cannot establish relative experimental affinity, and the predicted poses do not indicate activation or inhibition of the proteins or identify the constituents responsible for the in vivo effects.

### 3.3. In Vivo Experimental Results

#### 3.3.1. Effects of CPhGs on Activity Status, Body Weight, Exhaustive Swimming Time and Forelimb Grip Strength in Chemotherapy Mice

**Figure 3:**
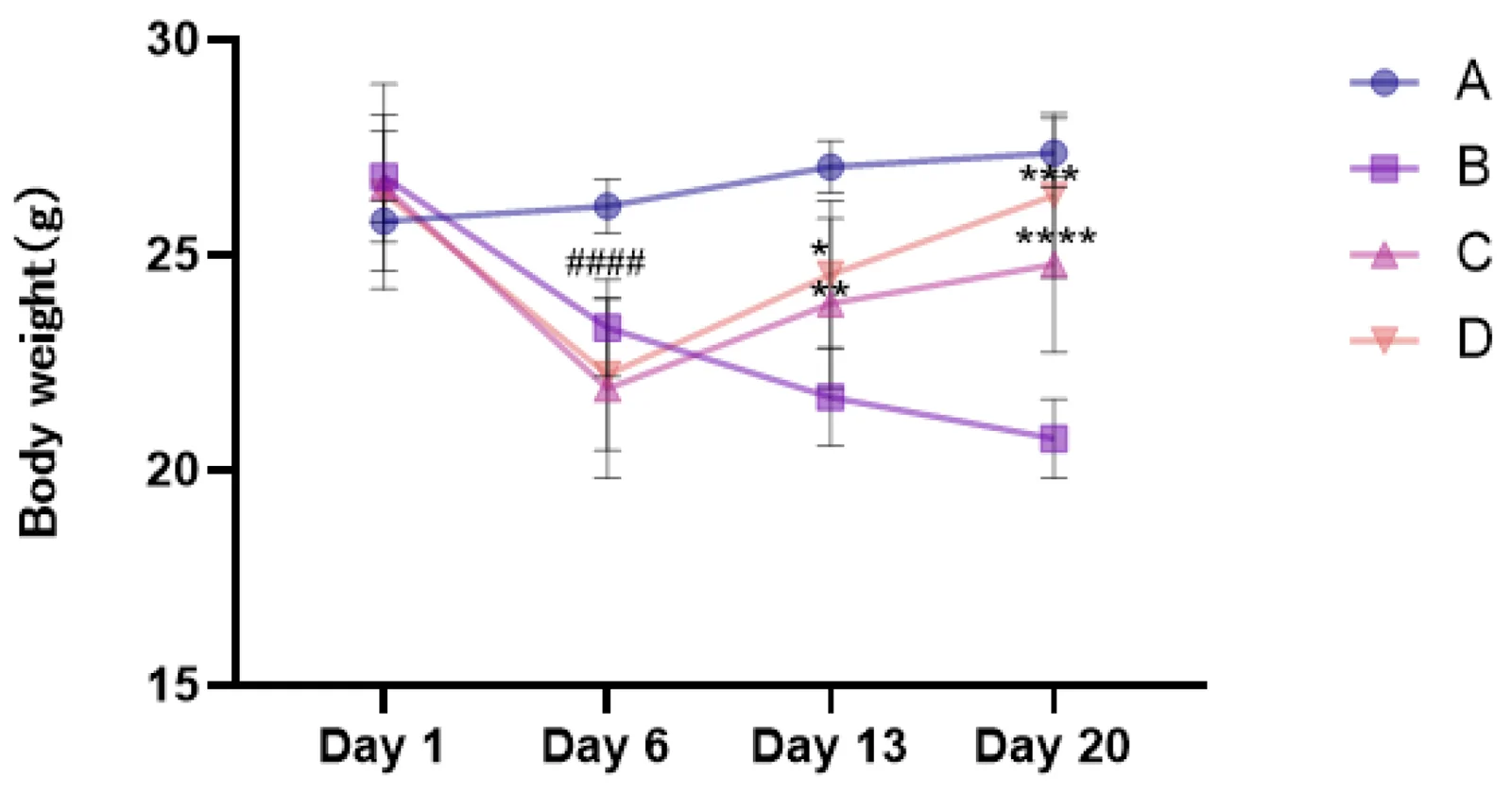
Changes in body weight of mice in each group at different stages. A: normal group; B: 5-FU model group; C: low-dose-CPhG group; D: high-dose-CPhG group. ^####^ *p* < 0.0001 vs. normal group; * *p* < 0.05, ** *p* < 0.01, *** *p* < 0.001, and **** *p* < 0.0001 vs. the model group. Data are mean ± SD (*n* = 10 mice/group). Two-way repeated-measure ANOVA with Geisser–Greenhouse correction and Tukey-adjusted comparisons within each time point was used.

**Figure 4:**
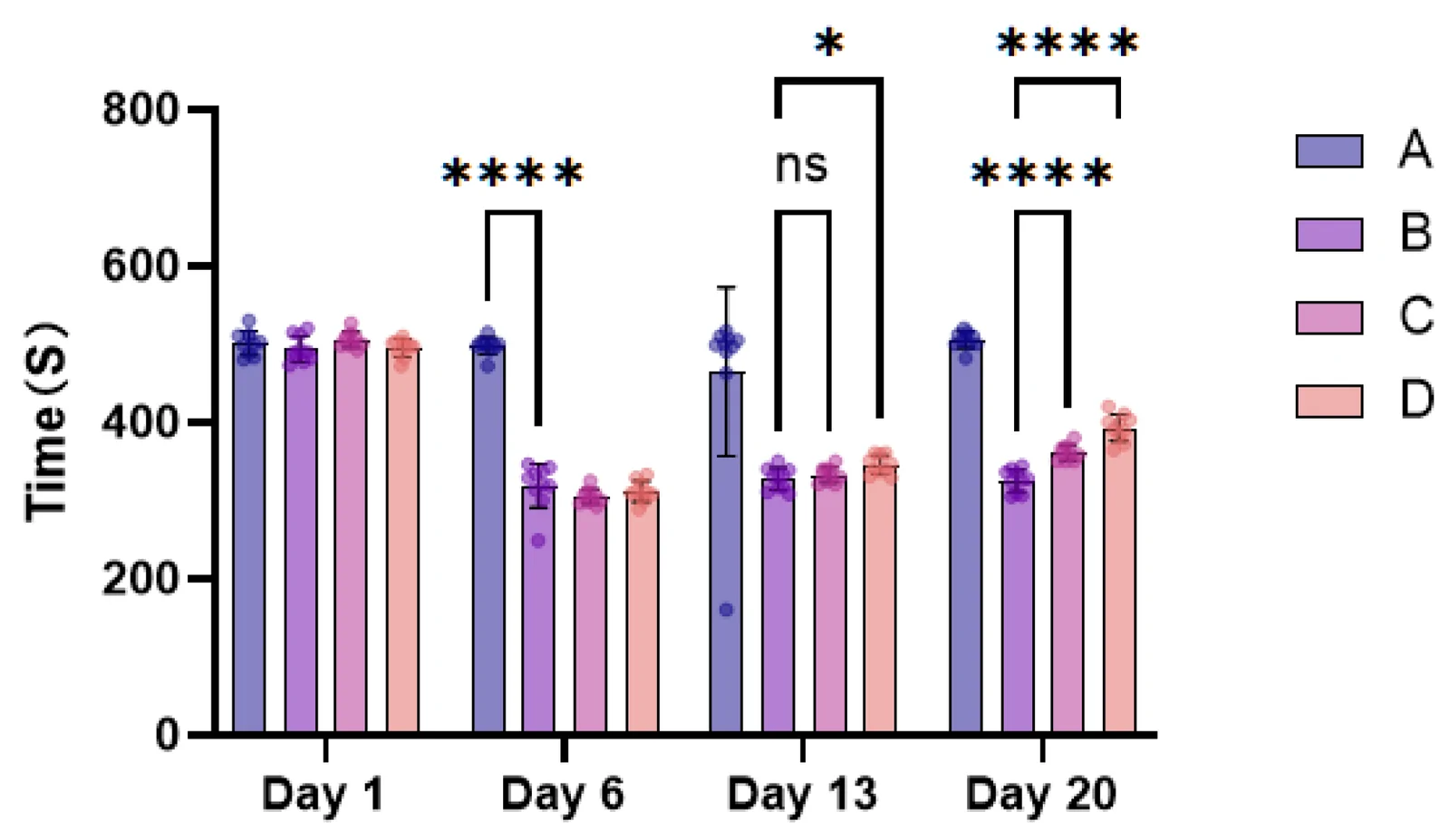
Changes in exhaustive swimming time in each group of mice at different stages (s, mean ± SD, *n =* 10). A: normal group; B: 5-FU model group; C: low-dose-CPhG group; D: high-dose-CPhG group. ns, not significant; * *p* < 0.05, **** *p* < 0.0001. Two-way repeated-measure ANOVA with Geisser–Greenhouse correction and Tukey-adjusted comparisons within each time point; brackets indicate the compared groups.

Changes in general condition, body weight, and exhaustive swimming time across groups are shown in Figure 3 and Figure 4. At baseline, no significant differences in body weight or exhaustive swimming time were observed among groups, indicating comparable starting conditions. During model establishment, model-group mice gradually developed signs of systemic illness including lethargy, hunched posture, reduced food intake, and disheveled fur. Following chemotherapy, model-group mice exhibited marked body weight loss and significantly shortened exhaustive swimming time compared to the normal group (*p* < 0.0001), with no spontaneous recovery during the subsequent observation period, consistent with 5-FU-induced systemic toxicity and a fatigue-like phenotype characterized by body weight loss and diminished exercise endurance. General-condition and food-intake descriptions here are qualitative observations. Longitudinal food-intake data for all experimental groups are provided in Supplementary Figure S53.

After CPhG intervention, mental status, spontaneous activity, and fur appearance improved to varying degrees in all treatment groups. Regarding body weight, mice in both low- and high-dose groups showed significant recovery compared to the model group during the mid-to-late treatment period (Figure 3), with the high-dose group approaching normal-group levels by the experimental endpoint. For exercise endurance, the high-dose group showed earlier onset of improvement, significantly prolonging exhaustive swimming time by mid-treatment (*p* < 0.01). By treatment completion, both low- and high-dose groups exhibited significantly prolonged exhaustive swimming time compared to the model group (*p* < 0.001 or *p* < 0.0001), with generally greater effects at the higher dose. These results suggest attenuation of 5-FU-induced weight loss and reduced exercise endurance. 

Exhaustive swimming time results at different stages are shown in Figure 4. At baseline, no significant differences in exhaustive swimming time were observed among groups; the normal group maintained stable swimming times throughout the experimental period. Following chemotherapy, model-group mice showed drastically shortened exhaustive swimming time compared to the normal group (*p* < 0.0001), with no spontaneous recovery after drug discontinuation, indicating marked impairment of exercise endurance by 5-FU chemotherapy. After CPhG intervention, exhaustive swimming time gradually increased. At mid-treatment (day 13), the high-dose group showed significantly prolonged exhaustive swimming time compared to the model group (*p* < 0.05), while the low-dose group showed improvement without reaching statistical significance. By treatment endpoint (day 20 in the longitudinal analysis; see the timing verification in Figure 4), both low- and high-dose groups exhibited significantly prolonged exhaustive swimming time compared to the model group (*p* < 0.0001), with the high-dose group showing greater improvement descriptively. However, by the experimental endpoint, exhaustive swimming time in treatment groups had not fully recovered to normal-group levels, indicating partial rather than complete recovery under the tested conditions. 

**Figure 5:**
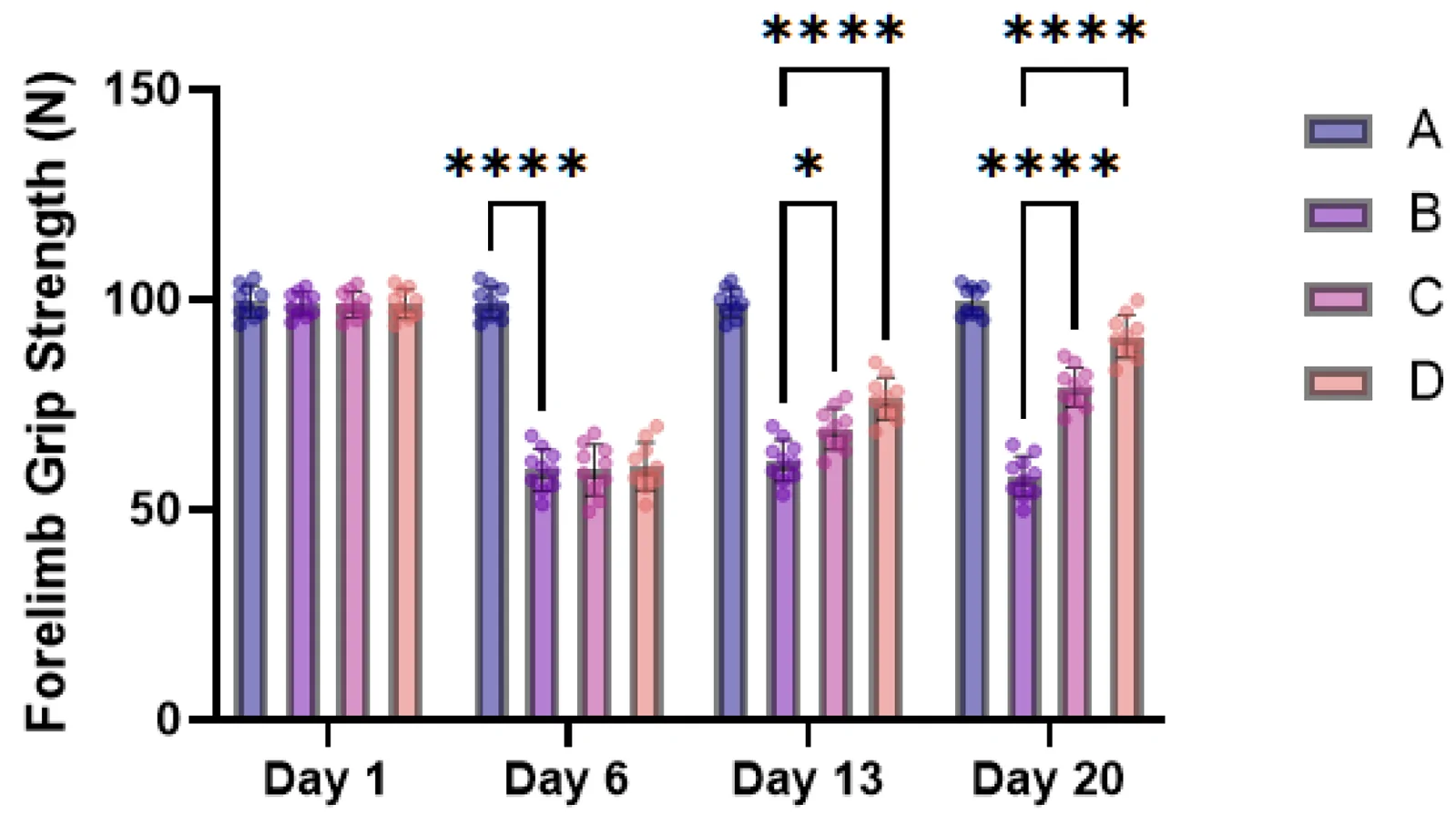
Changes in forelimb grip strength of mice in each group at different stages. A: normal group; B: 5-FU model group; C: low-dose-CPhG group; D: high-dose-CPhG group. Brackets indicate selected pairwise comparisons; * *p* < 0.05 and **** *p* < 0.0001. Data are presented as mean ± SD (*n =* 10 mice/group). Two-way repeated-measure ANOVA with Geisser–Greenhouse correction followed by Tukey-adjusted multiple comparisons within each time point was used.

Forelimb grip strength results are shown in Figure 5. At baseline, no significant differences in forelimb grip strength were observed among the four groups, indicating comparable baseline muscle function. Following 5-FU administration, forelimb grip strength in the model group was significantly reduced compared with the normal group on Day 6 (*p* < 0.0001), indicating impaired muscle function after chemotherapy. After CPhG intervention, forelimb grip strength gradually improved. On Day 13, both the low-dose- and high-dose-CPhG groups showed significantly greater grip strength than the model group (*p* < 0.05 and *p* < 0.0001, respectively). By Day 20, forelimb grip strength was significantly increased in both treatment groups compared with the model group (both *p* < 0.0001), with a generally greater improvement in the high-dose group. These findings suggest that CPhGs attenuated the decline in forelimb muscle function associated with 5-FU treatment.

#### 3.3.2. Comparison of Serum TNF-α, IL-6, CK, CK-MB, LDH, and LDH1 Levels

**Figure 6:**
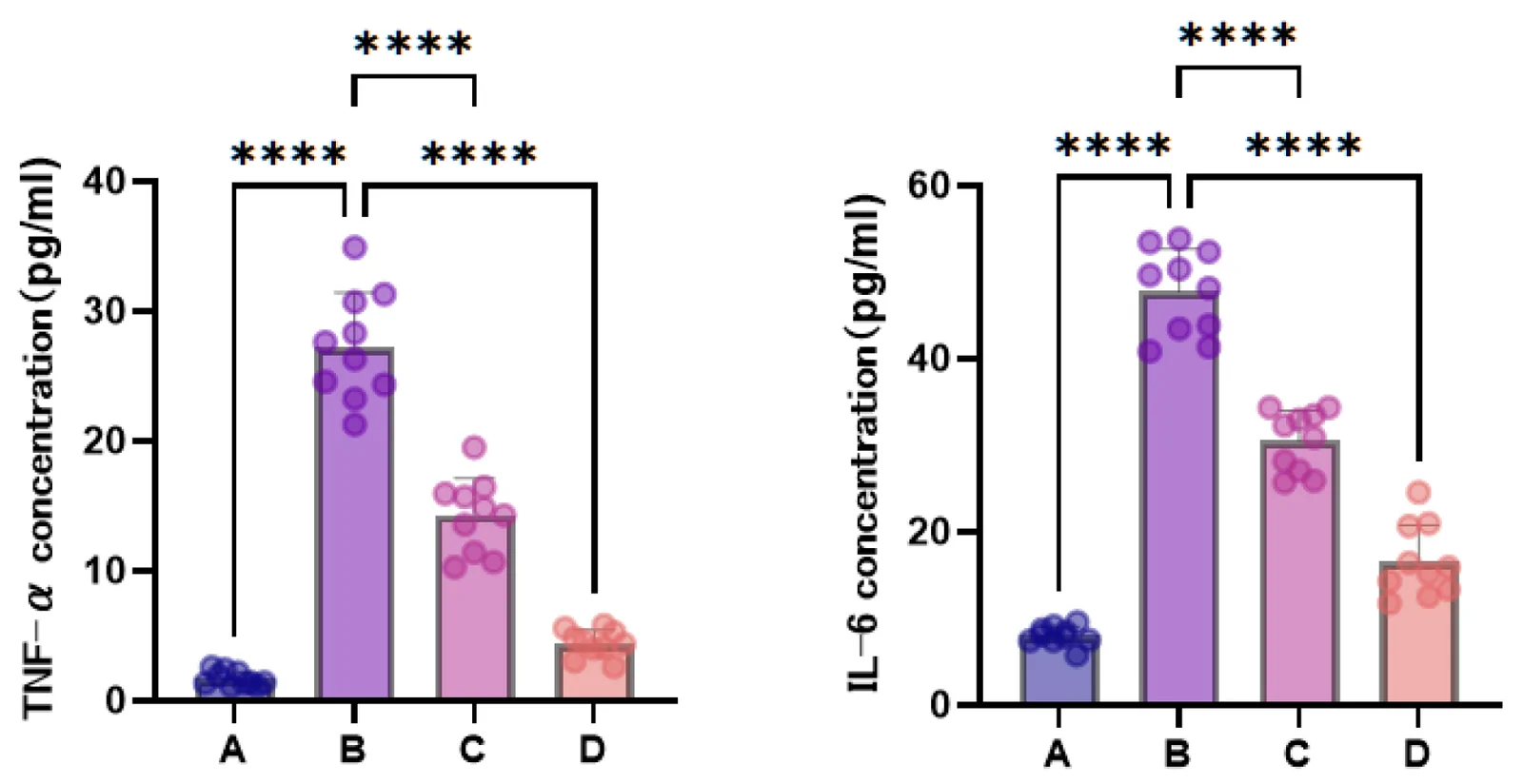
Serum TNF-α and IL-6 levels in different groups of mice (mean ± SD, *n =* 10). A: normal group; B: 5-FU model group; C: low-dose-CPhG group; D: high-dose-CPhG group. **** *p* < 0.0001. Each point represents one mouse; brackets indicate the compared groups.

Serum TNF-α and IL-6 results are shown in Figure 6. Compared to the normal group, model-group mice showed markedly elevated serum TNF-α and IL-6 levels (*p* < 0.0001), indicating significant systemic inflammatory response induced by 5-FU chemotherapy. After CPhG intervention, both low- and high-dose groups exhibited significantly reduced serum TNF-α and IL-6 levels compared to the model group (*p* < 0.0001), with the high-dose group showing more pronounced reduction approaching normal-group levels, suggesting that CPhGs were associated with lower circulating inflammatory cytokine levels, with generally greater effects at the higher dose.

**Figure 7:**
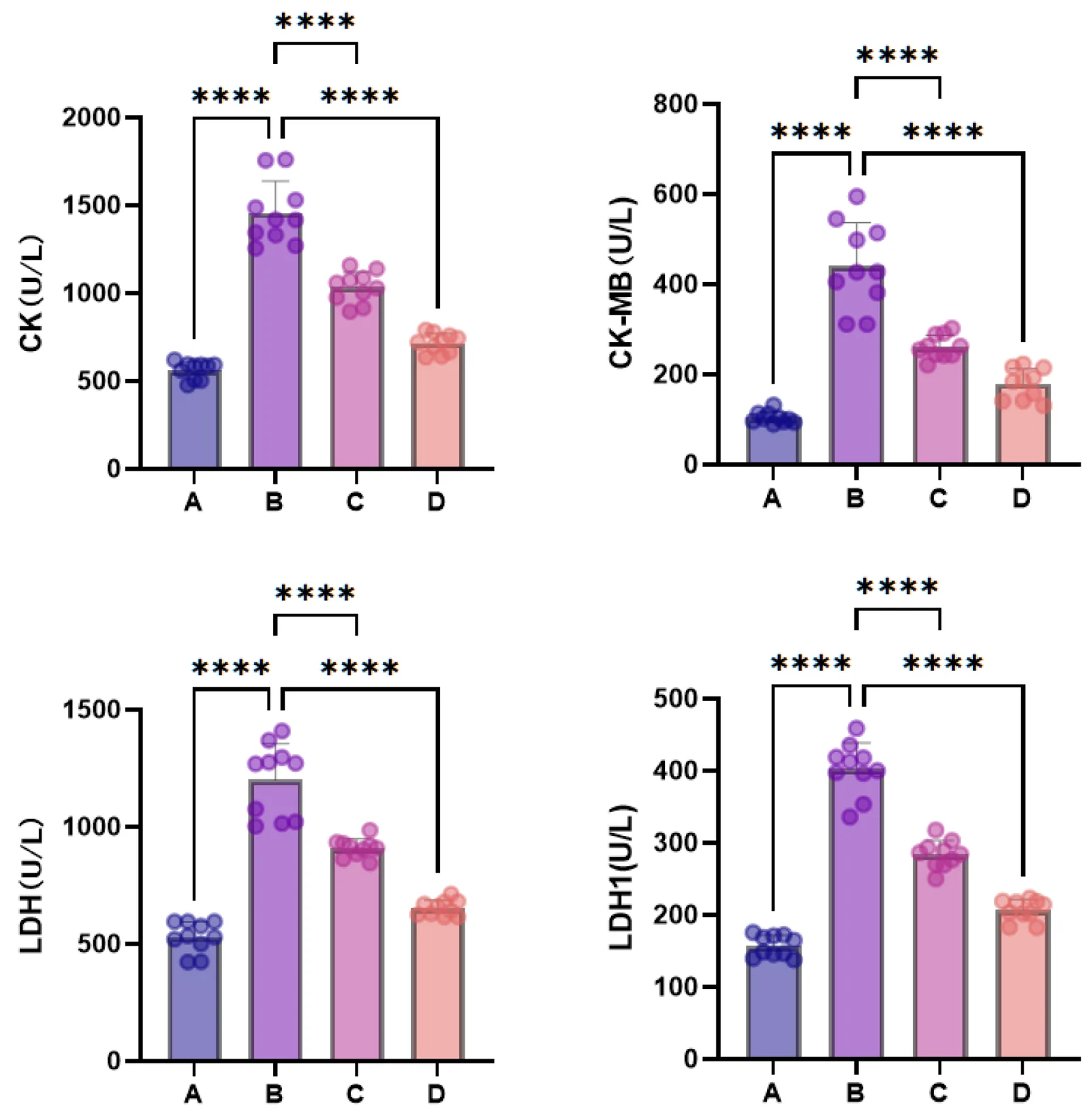
Serum CK, CK-MB, LDH, and LDH1 levels in different groups of mice (mean ± SD, *n =* 10). A: normal group; B: 5-FU model group; C: low-dose-CPhG group; D: high-dose-CPhG group. **** *p* < 0.0001. Each point represents one mouse; brackets indicate the compared groups.

Serum CK, CK-MB, LDH, and LDH1 results are shown in Figure 7. Compared to the normal group, model-group mice showed markedly elevated levels of all four indices (*p* < 0.0001), indicating non-specific tissue injury caused by 5-FU chemotherapy. After CPhG intervention, both low- and high-dose groups showed varying degrees of reduction in serum CK, CK-MB, LDH, and LDH1 levels compared to the model group (*p* < 0.0001), with the high-dose group showing more pronounced reduction approaching normal-group levels, suggesting that CPhGs were associated with lower non-specific tissue-injury markers. 

#### 3.3.3. Effects of CPhGs on Skeletal Muscle Histopathological Changes in Chemotherapy Mice

**Figure 8:**
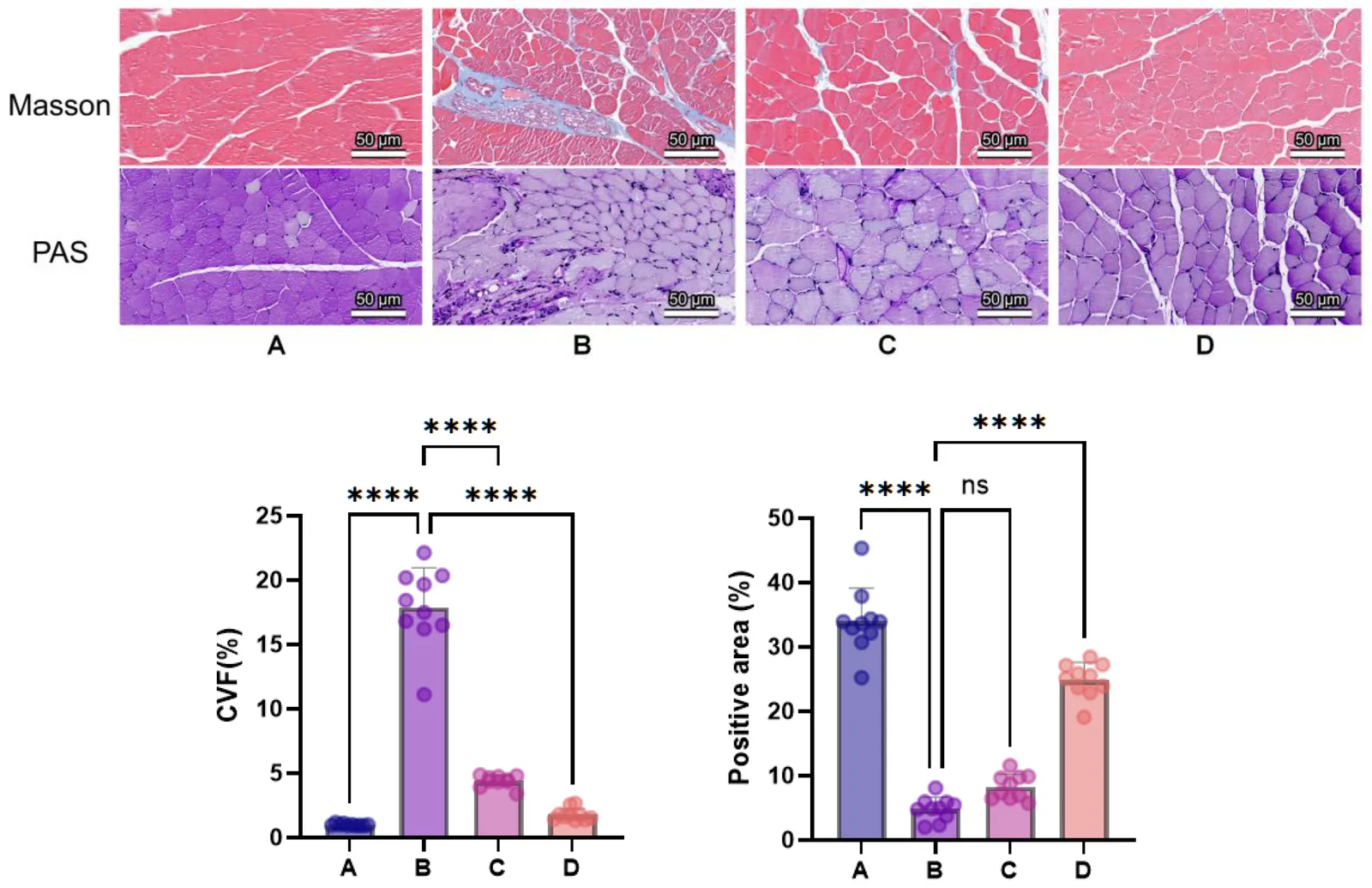
PAS and Masson staining of skeletal muscle tissue in mice of each group (original magnification: 20×; scale bar: 50 μm). (**A**): normal group; (**B**): 5-FU model group; (**C**): low-dose-CPhG group; (**D**): high-dose-CPhG group. ns, not significant; **** *p* < 0.0001. Quantitative data are mean ± SD (*n =* 10 mice/group); each point represents one animal, sampled using one section and one 20× field per parameter. Brackets indicate the compared groups.

As shown in Figure 8, PAS staining revealed that normal-group muscle fibers displayed regular morphology with uniform, abundant purplish-red PAS-positive carbohydrate-rich material in the sarcoplasm, showing uniform and intense staining. Compared to the normal group, the model group showed markedly weakened purplish-red staining appearing pale purple, with altered muscle fiber morphology, disorganized arrangement, and noticeably increased inflammation. Compared to the model group, treatment groups showed varying degrees of improvement, with deepened purplish-red color in the sarcoplasm and improved muscle fiber morphology. Masson staining showed that normal-group muscle tissue had intact morphology with only minimal blue collagen fiber deposition. Compared to the normal group, the model group showed widened endomysial spaces with prominent blue-stained collagen fibers surrounding muscle fibers (*p* < 0.0001), accompanied by sparse inflammatory cell infiltration and severe pathological damage. Compared to the model group, treatment groups showed improvement in both endomysial spaces and collagen fiber deposition around muscle fibers, with significant differences (*p* < 0.0001). The PAS-positive area showed no significant difference between the low-dose and model groups, whereas the high-dose group showed an increase (Figure 8). Muscle mass and myofiber cross-sectional area were not measured; morphology alone does not quantify muscle atrophy.

#### 3.3.4. Effects of CPhGs on Adipose Tissue Histopathological Changes in Chemotherapy Mice

**Figure 9:**
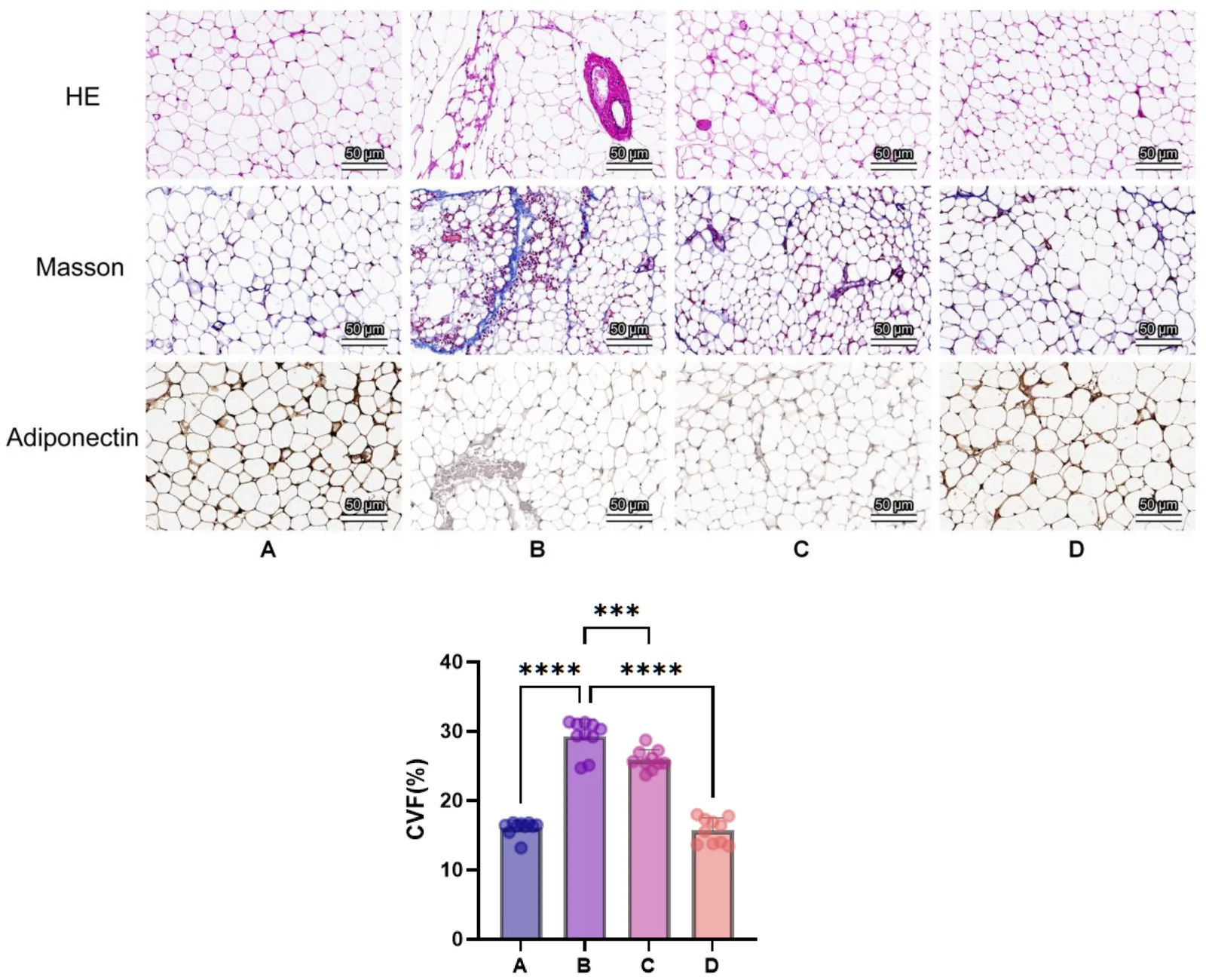
H&E, Masson, and adiponectin immunohistochemical staining of epididymal adipose tissue in mice of each group (original magnification: 20×; scale bar: 50 μm). (**A**): normal group; (**B**): 5-FU model group; (**C**): low-dose-CPhG group; (**D**): high-dose-CPhG group. *** *p* < 0.001, **** *p* < 0.0001. Rows show H&E, Masson, and adiponectin staining from top to bottom; the quantitative panel shows CVF. Quantitative data are mean ± SD (*n =* 10 mice/group); each point represents one animal, sampled using one section and one 20× field per parameter. Brackets indicate the compared groups.

As shown in Figure 9, H&E staining revealed that normal-group cells showed uniform size, regular morphology, and clear cell membranes. Compared to the normal group, model-group adipocytes appeared smaller, or displayed irregular size and morphology; some adipocytes were lysed with visible inflammatory-appearing foci. Compared to the model group, treatment groups showed less marked morphological alterations and fewer inflammatory foci.

Masson staining showed that normal-group adipocytes had only minimal blue collagen around vessels or in septa. Compared to the normal group, adipose tissue stroma showed prominent blue collagen deposition indicating fibrosis (*p* < 0.0001), with severe focal fibrosis. Compared to the model group, treatment groups showed reduced collagen deposition and decreased focal fibrosis (*p* < 0.0001).

Immunohistochemistry results showed that normal-group adipocyte cytoplasm exhibited strong positive cell expression with high proportion, appearing as deep brown staining. Compared to the normal group, the model group showed weaker adiponectin staining and fewer apparently positive areas. Compared to the model group, treatment groups showed varying degrees of recovery in positive cell proportion. These descriptions are qualitative. 

#### 3.3.5. Effects of CPhGs on Hepatic Tissue Histopathological Changes in Chemotherapy Mice

**Figure 10:**
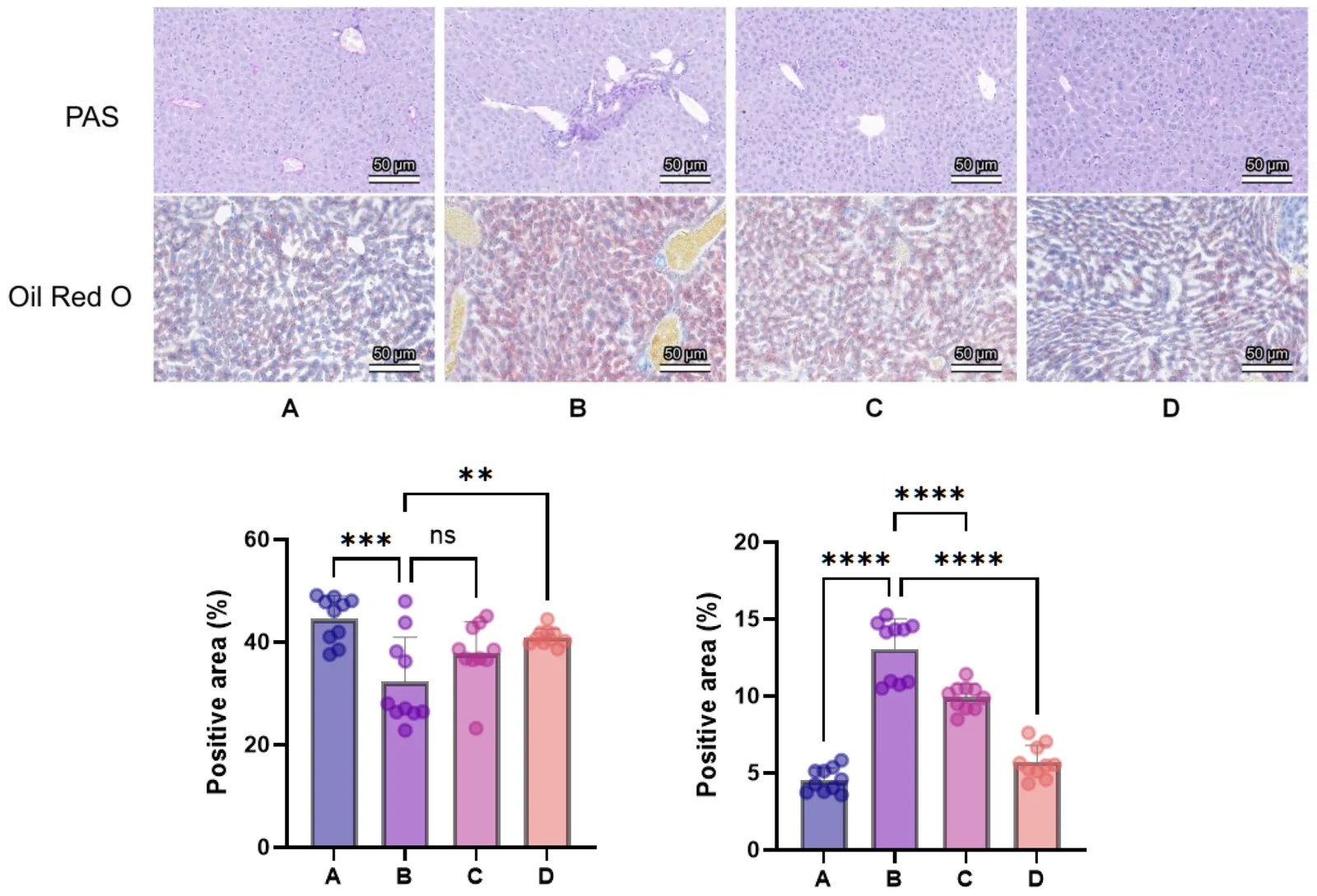
PAS and Oil Red O staining of liver tissue in mice of each group (original magnification: 20×; scale bar: 50 μm). (**A**): normal group; (**B**): 5-FU model group; (**C**): low-dose-CPhG group; (**D**): high-dose-CPhG group. ns, not significant; ** *p* < 0.01, *** *p* < 0.001, **** *p* < 0.0001. Quantitative data are mean ± SD (*n =* 10 mice/group); each point represents one animal, sampled using one section and one 20× field per parameter. Brackets indicate the compared groups.

As shown in Figure 10, PAS staining showed that the normal group exhibited large areas of intense purplish-red PAS-positive staining, reflecting abundant PAS-positive carbohydrate-rich material. Compared to the normal group, the model group showed markedly weakened or pale PAS staining, with only limited regions retaining PAS-positive material (*p* < 0.001). Compared to the model group, treatment groups showed varying degrees of recovery in PAS-positive purplish-red staining (*p* < 0.01). These PAS-positive signals were interpreted as carbohydrate-rich material rather than as a direct measurement of tissue glycogen.

Oil Red O staining showed that normal-group hepatocytes contained only minimal, scattered fine red lipid droplets. Compared to the normal group, the model group showed abundant, diffuse red-stained lipid droplets, which coalesced into larger areas in more severely affected regions (*p* < 0.0001). Compared to the model group, treatment groups showed varying degrees of reduction in the number and extent of red lipid droplets (*p* < 0.0001).

#### 3.3.6. Effects of CPhGs on Related Proteins in Muscle Tissue of Chemotherapy Mice

**Figure 11:**
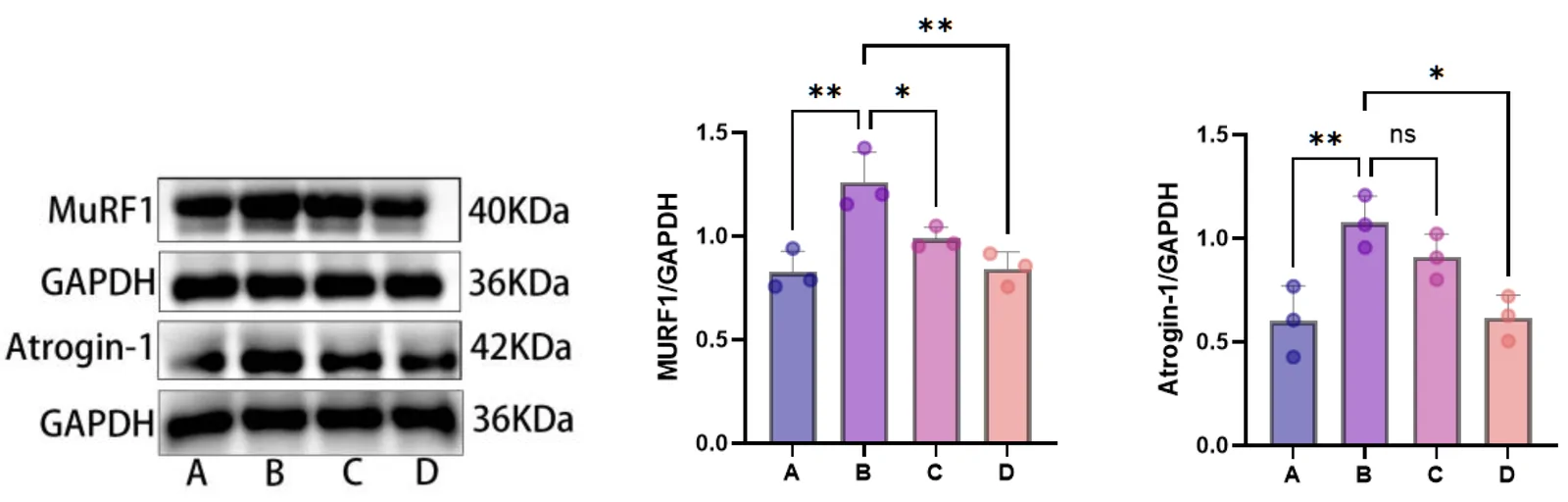
Electrophoresis blots of MuRF1 and Atrogin-1 proteins in mouse skeletal muscle tissue. A: normal group; B: 5-FU model group; C: low-dose-CPhG group; D: high-dose-CPhG group. ns, not significant; * *p* < 0.05, ** *p* < 0.01. All samples were resolved on the same gel, transferred simultaneously, and developed in parallel, ensuring direct comparability of band intensities across lanes. Representative blots and mean ± SD are shown for three independent mice/group, each from a different cage; each point represents one mouse. Figure 11 and Figure 12 used the same animals and lysates. Brackets indicate compared groups.

Western blot was used to detect expression levels of ubiquitin E3 ligases MuRF1 and Atrogin-1 in skeletal muscle tissue of each group, with GAPDH as the internal reference protein. Results are shown in Figure 11. The biological sample size was *n =* 3 independent mice/group; the same animals and lysates were used for the results shown in Figure 11.

Compared to the normal control group, 5-FU model-group mice showed significantly elevated relative expression of MuRF1 and Atrogin-1 proteins in skeletal muscle (*p* < 0.05), consistent with altered expression of UPS-associated atrophy markers; protein degradation rates and muscle mass were not directly measured.

Compared to the 5-FU model group, both low-dose (250 mg/kg) and high-dose (500 mg/kg) CPhG groups showed reduced MuRF1 expression, whereas the displayed Atrogin-1 comparison was significant only for the high-dose group (Figure 11). These results suggest lower expression of atrophy-associated proteins, with generally greater effects at the higher dose.

**Figure 12:**
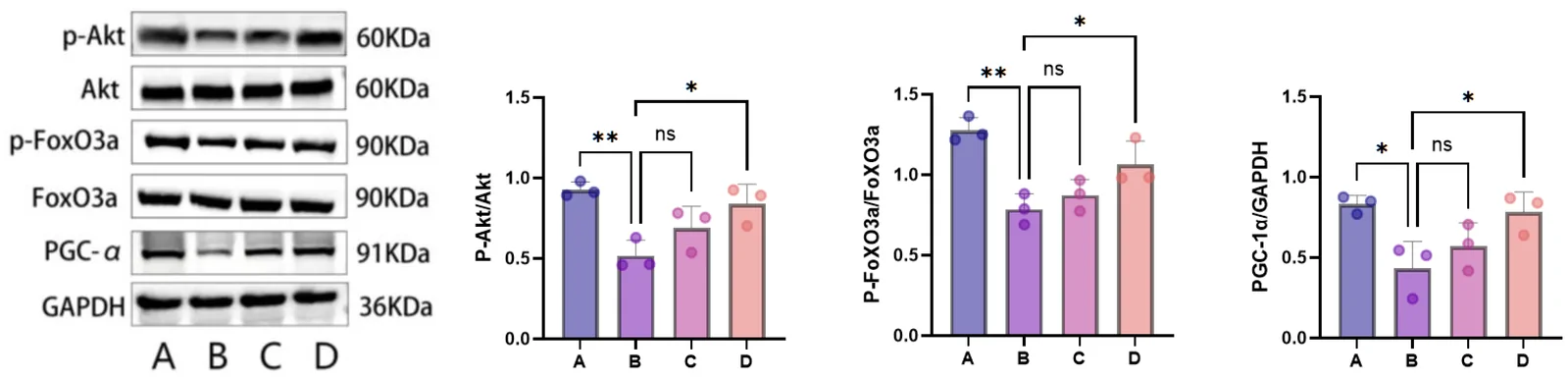
Electrophoresis blots of PGC-1α, Akt, p-Akt, FoxO3a, and p-FoxO3a proteins in mouse skeletal muscle tissue. A: normal group; B: 5-FU model group; C: low-dose-CPhG group; D: high-dose-CPhG group. ns, not significant; * *p* < 0.05, ** *p* < 0.01. All samples were resolved on the same gel, transferred simultaneously, and developed in parallel, ensuring direct comparability of band intensities across lanes. Representative blots and mean ± SD are shown for three independent mice/group, each from a different cage; each point represents one mouse. Figure 11 and Figure 12 use the same animals and lysates. Brackets indicate compared groups.

To further explore molecular changes associated with CPhG treatment, we examined expression levels of Akt/FoxO3a signaling pathway-related proteins (Akt, p-Akt, FoxO3a, p-FoxO3a) and PGC-1α in skeletal muscle tissue of each group, with GAPDH as the internal reference protein. Results are shown in Figure 12. Total Akt and total FoxO3a protein expression levels in skeletal muscle showed no significant differences among groups, while the observed group differences primarily concerned phosphorylation ratios. Compared to the normal group, model group mice showed significantly decreased p-Akt/Akt and p-FoxO3a/FoxO3a ratios (*p* < 0.01), and markedly downregulated PGC-1α protein expression (*p* < 0.05) in skeletal muscle, consistent with altered Akt/FoxO3a phosphorylation and lower expression of an energy-metabolism-associated protein. After CPhG intervention, the low-dose group showed some degree of recovery in these indices, though differences compared to the model group did not reach statistical significance. The high-dose group showed significantly elevated p-Akt/Akt and p-FoxO3a/FoxO3a ratios and PGC-1α protein expression levels compared to the model group (*p* < 0.05). These results associate CPhG treatment with changes in Akt/FoxO3a phosphorylation and PGC-1α expression. They do not establish pathway causality, mitochondrial functional recovery, or a dose-dependent relationship.

## 4. Discussion

Chemotherapy-induced fatigue, systemic toxicity, and skeletal muscle abnormalities represent major constraints on quality of life and chemotherapy tolerance in cancer patients. To thoroughly investigate the pathological mechanisms and identify effective therapeutic interventions, establishing well-characterized animal models that reproduce relevant features of chemotherapy toxicity is essential. Clinical chemotherapy regimens commonly employ anthracycline antineoplastic antibiotics (doxorubicin), antimetabolites (5-FU), alkaloids (paclitaxel), alkylating agents (cyclophosphamide), cytotoxic agents (oxaliplatin, cisplatin), or combination protocols (CAF regimen: cyclophosphamide, doxorubicin, 5-FU; platinum–taxane combinations, etc.). This segment of experiments used tumor-free animal models to avoid confounding effects between tumor presence and drug antitumor activity [19]. Among these agents, 5-FU is a widely used antimetabolite chemotherapy drug that can induce systemic toxicity and impair physical performance. Exposure of tumor-free mice to 5-FU provides a useful experimental model for evaluating chemotherapy-induced fatigue-like behavior and systemic tissue injury, although it does not reproduce the tumor–host interactions characteristic of cancer cachexia or the full clinical syndrome of cancer-related fatigue. This study employed 5-FU to induce a chemotherapy mouse model, used GEO transcriptomic analysis to provide external pathway context, and systematically evaluated the effects of CPhGs across behavioral, serum biochemical, histopathological, and skeletal muscle signaling pathway levels, while preliminarily investigating the associated molecular alterations. Molecular docking was further used as an exploratory approach to predict possible interactions between major characterized CPhG constituents and selected proteins.

The role of GEO public transcriptome analysis in this study was to provide exploratory biological context and a rationale for subsequent index selection. Since the included GEO datasets did not contain CPhG-treated samples, the related results were not used to identify direct CPhG targets or as direct pharmacological evidence. Their value lies primarily in highlighting biological processes related to muscle contraction, fatty acid oxidation, mitochondrial energy metabolism, FoxO/UPS, and inflammation/STAT pathways from FOLFIRI/chemotherapy and cancer cachexia skeletal muscle injury backgrounds, thereby providing external rationale for subsequently selecting protein detection indices such as Akt/FoxO3a, MuRF1, Atrogin-1, and PGC-1α. The primary FOLFIRI dataset did not contain genes meeting the prespecified FDR and fold-change criteria; therefore, the transcriptomic findings were interpreted mainly at the pathway level and together with the established biological functions of the selected markers.

Body weight and exercise endurance serve as the most intuitive indicators reflecting overall physiological status and chemotherapy toxicity. Model-group mice exhibited progressive body weight loss from early model establishment, accompanied by signs of systemic illness, including hunched posture, reduced mobility, and disheveled fur. Simultaneously, weight-loaded exhaustive swimming time was significantly shortened, indicating markedly impaired exercise endurance. Forelimb grip strength was also markedly reduced following 5-FU exposure, indicating impaired skeletal muscle functional performance in addition to reduced exercise endurance. Chemotherapy-related systemic illness, including reduced food intake and metabolic disturbance, may contribute to changes in body weight and physical performance [18]. After CPhG intervention, the trend of body weight loss was alleviated, and exhaustive swimming time was significantly prolonged compared to the model group. Forelimb grip strength also progressively improved after CPhG treatment, with significant increases observed in both treatment groups during the intervention period and generally greater improvement in the high-dose group. Notably, the high-dose group (500 mg/kg) showed no statistical difference in body weight compared to the normal group at the experimental endpoint (*p* > 0.05), consistent with attenuation of 5-FU-induced body weight loss and impaired exercise endurance. These findings suggest that CPhGs may improve the overall fatigue-like phenotype induced by 5-FU. Food intake was monitored during the experiment; however, changes in appetite may also contribute to the observed effects on body weight and physical performance.

Regarding skeletal muscle injury assessment, CK, CK-MB, LDH, and LDH1 are circulating indicators of tissue injury rather than skeletal-muscle-specific biomarkers. In particular, CK-MB and LDH1 may also reflect cardiac involvement [19]. In this study, serum levels of CK, CK-MB, LDH, and LDH1 were markedly elevated in model-group mice compared to the normal group, indicating increased systemic tissue injury following 5-FU exposure. After CPhG intervention, these indices decreased to varying degrees, with the high-dose group approaching normal levels, consistent with attenuation of chemotherapy-associated tissue injury. Regarding tissue morphology, PAS staining reflects PAS-positive carbohydrate-rich material rather than providing a direct biochemical measurement of muscle glycogen, whereas Masson staining assesses collagen fiber deposition and tissue fibrosis degree [20,21]. Model-group mice showed markedly reduced sarcoplasmic PAS-positive staining, disorganized muscle fiber arrangement, and extensive intermuscular collagen deposition, indicating marked histological alterations in skeletal muscle after 5-FU exposure. After CPhG intervention, PAS-positive staining increased and collagen deposition decreased markedly, suggesting that it alleviates chemotherapy-induced skeletal muscle damage at the histological level.

At the molecular mechanism level, the aforementioned GEO results suggested that FoxO/UPS and energy metabolism-related pathways may participate in chemotherapy- or cachexia-related skeletal muscle injury; therefore, this study further examined Akt/FoxO3a pathway, MuRF1/Atrogin-1, and PGC-1α protein expression in animal experiments. The ubiquitin-proteasome system (UPS) plays an important role in skeletal muscle protein turnover. MuRF1 and Atrogin-1, as skeletal muscle-specific E3 ubiquitin ligases, mediate ubiquitination and degradation of myofibrillar proteins and are significantly upregulated in various pathological muscle atrophy models, serving as recognized molecular markers of muscle atrophy [22]. Western blot results in this study showed markedly elevated MuRF1 and Atrogin-1 protein expression in model group mouse skeletal muscle, consistent with atrophy-associated molecular alterations. After CPhG intervention, expression of both proteins was markedly downregulated, suggesting that CPhG treatment was associated with modulation of muscle protein-turnover-related signaling. MuRF1 and Atrogin-1 expression is negatively regulated by the PI3K/Akt signaling pathway. Upon phosphorylation and activation, Akt phosphorylates and inactivates FoxO3a, preventing its nuclear entry to initiate transcription of atrophy-related genes. This study further found that p-Akt/Akt and p-FoxO3a/FoxO3a ratios were markedly decreased in model-group mouse skeletal muscle, consistent with altered Akt/FoxO3a phosphorylation status. Simultaneously, model-group PGC-1α protein expression was downregulated, indicating alteration of a mitochondrial-biogenesis-associated molecular marker, consistent with declined exercise endurance and glucose–lipid metabolic dysfunction phenotypes in mice. High-dose CPhG intervention significantly upregulated p-Akt/Akt and p-FoxO3a/FoxO3a ratios and PGC-1α protein expression, indicating that the beneficial effects of CPhGs were associated with modulation of Akt/FoxO3a signaling and restoration of PGC-1α expression. These molecular changes were consistent with the improvements in exercise endurance and skeletal muscle histological features observed after CPhG treatment.

Additionally, this study found that 5-FU chemotherapy also caused marked damage to adipose tissue and liver. Model-group mouse adipocytes showed smaller and irregular morphology, inflammatory-appearing foci, obvious interstitial fibrosis, and weaker adiponectin staining, consistent with adipose tissue injury associated with 5-FU-induced systemic toxicity. The liver, as the central organ for lipid metabolism and glycogen reserve, can be assessed for lipid deposition and PAS-positive carbohydrate-rich material through Oil Red O and PAS staining, respectively. Model-group livers showed diffuse lipid droplet deposition and markedly reduced PAS-positive staining, indicating altered hepatic lipid accumulation and carbohydrate-rich staining after 5-FU exposure. After CPhG intervention, these pathological changes in adipose tissue and liver were alleviated to varying degrees, suggesting multi-organ-protective effects, consistent with previously reported anti-inflammatory, antioxidant, and metabolic regulatory activities of CPhGs [23,24,25]. Taken together, the histological findings suggest that the protective effects of CPhGs in this model were not confined to skeletal muscle but were accompanied by improvements in adipose tissue and hepatic morphology.

This study employed blind docking technology via the CB-Dock2 platform to further explore possible interactions between acteoside and echinacoside and selected proteins at the molecular level. The predicted Vina scores and binding poses suggested potential interactions of both compounds with Akt, MuRF1, and IL-6. These computational findings provide exploratory structural information regarding possible compound–protein interactions, rather than direct evidence of target engagement or functional activation/inhibition of these proteins. Accordingly, the molecular docking analysis should be regarded as hypothesis-generating and complementary to the in vivo findings. Importantly, the biological effects observed in the animal experiments should be attributed to the complete CPhG preparation; echinacoside and acteoside are major characterized constituents that may contribute to the overall activity, but their individual contributions require further investigation.

## 5. Conclusions

In summary, GEO public transcriptome analysis provided disease pathway-level clues for subsequent index selection in this study but was not interpreted as direct evidence of CPhG targets or pharmacological effects; the observed effects of CPhGs are primarily supported by the 5-FU mouse experimental results in this study. CPhGs were associated with protective effects against 5-FU chemotherapy-induced systemic toxicity at multiple levels, including attenuation of body weight loss, improvement of exercise endurance, alleviation of skeletal muscle histological abnormalities, modulation of Akt/FoxO3a signaling and restoration of PGC-1α expression, reduction of MuRF1 and Atrogin-1 expression, improvement of adipose tissue histological alterations and fibrosis, and attenuation of hepatic pathological changes, supporting its potential value for further investigation in chemotherapy-induced fatigue and systemic toxicity. This study has certain limitations: GEO analysis provides contextual pathway information rather than direct CPhG target identification; direct muscle morphometric measurements and pathway-blockade experiments were not performed; the specific contributions of individual CPhG constituents and their molecular targets require further elucidation in subsequent research.

## Figures and Tables

**Table 1:** AutoDock Vina scores of major characterized constituents with selected proteins.

Active Ingredient	Protein	Vina Score (kcal/mol)
Acteoside	IL-6	−7.7
Acteoside	MuRF1	−8.8
Acteoside	AKT1	−10.5
Echinacoside	IL-6	−7.1
Echinacoside	MuRF1	−7.9
Echinacoside	AKT1	−9.9

## Data Availability

Publicly available datasets analyzed in this study are available from the Gene Expression Omnibus under accession numbers GSE80473, GSE121972, and GSE77745. Other data supporting the findings are available from the corresponding authors upon reasonable request.

## Supplementary Materials

The following supporting information can be downloaded at https://www.mdpi.com/article/10.3390/biomedicines14102236/s1: Figures S1–S53, complete GEO public transcriptomic analyses of GSE121972, GSE77745, and GSE80473, including quality-control analyses, differential-expression analyses, enrichment analyses, GSEA plots, and key-gene expression analyses; Figure S53, longitudinal food-intake data of mice in each experimental group.
