# Supplementary material for: Total Phenylethanoid Glycosides from Cistanche tubulosa Alleviate 5-Fluorouracil-Induced Fatigue-Like Behavior and Skeletal Muscle Injury in Mice
Source: Biomedicines. 2026 Oct 2;14(10):2236. doi: 10.3390/biomedicines14102236 (PMC13701381; doi:10.3390/biomedicines14102236)
Supplement: Supplementary file 1 [file biomedicines-14-02236-s001.zip › biomedicines-4584762-supplementary.pdf]

# Complete Results of the GEO Public Transcriptomic Analysis (Including All Figures)

Note: This section systematically presents the results of the GEO public transcriptomic analysis and provides a rationale for selecting Akt/FoxO3a/MuRF1/Atrogin-1/PGC-1α and other markers for subsequent animal experiments. The GEO datasets did not include samples treated with total phenylethanoid glycosides from Cistanche deserticola (CPhGs); therefore, these data cannot be used as evidence of direct targets or therapeutic efficacy of CPhGs.

## 1 Dataset inclusion and analytical positioning

GEO datasets were screened at three levels: 5-FU/FOLFIRI chemotherapy-associated skeletal muscle injury, skeletal muscle wasting in cancer cachexia, and chemotherapy-induced myotoxicity. GSE80473 was included as the primary analysis dataset, GSE121972 as the validation dataset for skeletal muscle in cancer cachexia, and GSE77745 as an auxiliary validation dataset for chemotherapy-induced myotoxicity. GSE80473 was the dataset most closely related to chemotherapy-associated skeletal muscle injury, although its sample size was small. GSE121972 showed stronger differential expression signals and was suitable for validating abnormalities in FoxO/UPS, inflammation/STAT signaling, mitochondrial energy metabolism, and ECM remodeling during cachexia-associated skeletal muscle wasting. GSE77745 was an acute doxorubicin myotoxicity model and provided a lower level of evidence than the other two datasets.

| Dataset | Tissue | Model/treatment | Sample size | Groups | Role in analysis |
|---|---|---|---|---|---|
| GSE80473 | Quadriceps muscle | Male CD2F1 mice without tumors were treated with FOLFIRI or vehicle for up to 5 weeks; FOLFIRI comprised 5-FU, leucovorin, and CPT-11. | 8 samples; Vehicle, n = 4; FOLFIRI, n = 4 | Vehicle versus FOLFIRI | Included; primary analysis dataset |
| GSE121972 | Skeletal muscles: m. extensor digitorum longus (EDL) and m. soleus (SOL); heart samples were also included. | C26 tumor cachexia model in male CD2F1 mice; some animals underwent whole-body vibration training (WBV). | 58 samples: EDL, 23; SOL, 23; heart, 12. The principal validation comparison used Control (n = 6) versus Tumour bearing (n = 6) within EDL and SOL, excluding the WBV groups; heart samples were excluded from the primary skeletal muscle validation. | Control, Control + WBV, Tumour bearing, and Tumour bearing + WBV; the validation comparison was Control versus Tumour bearing, analyzed separately for EDL and SOL. | Included; cancer cachexia validation dataset |
| GSE77745 | Tibialis anterior muscle | Intraperitoneal doxorubicin induced chemotherapy-associated muscle atrophy; an sAcvR2B-Fc pretreatment group was also included. | 15 samples; Control (PBS), n = 5; Doxorubicin, n = 5; Doxorubicin + sAcvR2B-Fc, n = 5 | Control (PBS) versus Doxorubicin; the Doxorubicin + sAcvR2B-Fc group was not included in the primary validation comparison. | Included; auxiliary chemotherapy myotoxicity validation dataset |

## 2 Overall differential expression results

Differential expression analysis was performed for each dataset using the prespecified comparisons. The local edgeR reanalysis of GSE80473 included 12,513 genes and identified no genes meeting the stringent thresholds of FDR < 0.05 and |log2FC| > 1; the minimum FDR was 0.0581. This finding indicated limited statistical power for

detecting individual differentially expressed genes in the primary dataset. Accordingly, GSE80473 was more suitable for evaluating pathway-level changes using GSEA based on the ranked list of all genes. The depositor-provided edgeR table for GSE80473 contained 32 genes meeting the stringent criteria. However, because this table was provided as a supplementary depositor result in GEO, it was used only for sensitivity assessment and not as the principal conclusion of the locally reproducible analysis. The EDL and SOL validation analyses of GSE121972 identified 787 and 558 stringent differentially expressed genes, respectively, indicating more stable transcriptomic alterations in the cancer cachexia skeletal muscle wasting model. Only one stringent differentially expressed gene was identified in GSE77745, which was therefore used as auxiliary evidence for chemotherapy-induced myotoxicity.

| Analysis | Genes tested | Stringent DEGs | Upregulated | Downregulated | Minimum FDR |
|---|---|---|---|---|---|
| GSE80473 local edgeR | 12513 | 0 | 0 | 0 | 0.05814 |
| GSE80473 depositor edgeR | 12896 | 32 | 24 | 8 | 9.52e-04 |
| GSE77745 limma | 20116 | 1 | 1 | 0 | 0.01895 |
| GSE121972 EDL limma | 20353 | 787 | 390 | 397 | 5.44e-08 |
| GSE121972 SOL limma | 20353 | 558 | 249 | 309 | 1.83e-08 |

## 3 Results of the primary dataset GSE80473

GSE80473 comprised RNA-seq data from the quadriceps muscles of non-tumor-bearing mice treated with FOLFIRI, a regimen containing 5-FU, leucovorin, and CPT-11. The quality-control plots showed that the samples were generally suitable for downstream analysis, although the within-group dispersion observed in the PCA suggested that the small sample size and interindividual variation might affect the detection of differential expression at the single-gene level. Local edgeR analysis of the supplementary count matrix deposited in GEO identified no significant genes at the stringent thresholds, although the volcano plot showed nominal P-value signals for several genes. The top-ranked genes included Exoc3l4, Hamp2, Klf10, Scd2, Fhl3, Lrp2bp, Rpl13, Prkcz, Fam196b, and Ccl8. Exoc3l4 had the lowest FDR, but it did not reach 0.05.

| Gene | logFC | logCPM | P value | FDR |
|---|---|---|---|---|
| Exoc3l4 | 2.767 | 2.715 | 4.65e-06 | 0.05814 |
| Hamp2 | 2.279 | 1.287 | 1.84e-05 | 0.1048 |
| Klf10 | 1.031 | 5.536 | 2.90e-05 | 0.1048 |
| Scd2 | 1.609 | 6.534 | 3.60e-05 | 0.1048 |
| Fhl3 | -1.24 | 6.67 | 4.19e-05 | 0.1048 |
| Lrp2bp | 1.51 | 4.857 | 6.36e-05 | 0.1327 |
| Rpl13 | 1.385 | 4.667 | 1.04e-04 | 0.1682 |
| Prkcz | 1.37 | 2.074 | 1.36e-04 | 0.1682 |
| Fam196b | 2.199 | 0.8197 | 1.65e-04 | 0.1682 |
| Ccl8 | 3.781 | 1.954 | 1.70e-04 | 0.1682 |

GSEA based on the ranked list of all genes in GSE80473 showed significant alterations in multiple metabolic and muscle function-related terms after FOLFIRI treatment, including fatty acid oxidation (NES = -2.41, FDR = 5.38e-08); fatty acid catabolic process (NES = -2.40, FDR = 5.38e-08); lipid oxidation (NES = -2.31, FDR = 5.38e-08); fatty acid metabolic process (NES = -1.89, FDR = 3.98e-07); fatty acid beta-oxidation (NES = -2.25, FDR = 1.79e-05); muscle system process (NES = -1.49, FDR = 0.0024); regulation of muscle system process (NES = -1.59, FDR = 0.0041); striated muscle cell development (NES = -1.58, FDR = 0.0056).

At the KEGG level, metabolism-related pathways were also altered, including Fatty acid degradation (NES = -2.33, FDR = 5.06e-05); Regulation of lipolysis in adipocytes (NES = -2.21, FDR = 9.76e-05); Propanoate metabolism (NES = -2.26, FDR = 1.25e-04); cGMP-PKG signaling pathway (NES = -1.86, FDR = 2.52e-04); Valine, leucine and isoleucine degradation (NES = -2.13, FDR = 3.47e-04); Fatty acid metabolism (NES = -1.68, FDR = 0.0220).

Collectively, GSE80473 supported the presence of altered transcriptional programs related to fatty acid oxidation, energy metabolism, and muscle contraction in FOLFIRI/chemotherapy-associated skeletal muscle injury.

However, it did not support describing any individual key gene as significantly differentially expressed after FDR correction.

# 4 Sensitivity assessment using the depositor-provided edgeR table for GSE80473

The depositor-provided edgeR statistics table in the GEO supplementary files included 12,896 genes and identified 32 stringent differentially expressed genes, of which 24 were upregulated and 8 were downregulated. Because the number of stringent differentially expressed genes differed from that obtained by the local edgeR reanalysis of the count matrix, this result was considered only as a sensitivity assessment. Enrichment results from the depositor-provided table were concentrated in terms related to plasma lipoprotein particle organization, protein-lipid complex remodeling, and humoral immune responses, suggesting that FOLFIRI treatment may also be accompanied by alterations in lipid transport and immune-related processes.

| Gene | logFC | P value | FDR |
|---|---|---|---|
| Dnah5 | 8.147 | 1.48e-07 | 9.52e-04 |
| Exoc3l4 | 2.76 | 1.26e-07 | 9.52e-04 |
| Slc4a1 | 3.6 | 1.55e-06 | 0.004997 |
| Fam107a | -2.301 | 1.29e-06 | 0.004997 |
| Mup6 | 4.6 | 4.99e-06 | 0.01126 |
| Scd2 | 1.606 | 5.24e-06 | 0.01126 |
| Fga | 4.886 | 7.28e-06 | 0.01282 |
| Chac1 | -1.787 | 7.95e-06 | 0.01282 |
| Ccl8 | 3.659 | 1.00e-05 | 0.01306 |
| Apoa1 | 4.55 | 1.01e-05 | 0.01306 |

Representative GO BP terms enriched among differentially expressed genes in the depositor-provided edgeR table included plasma lipoprotein particle organization (Count = 5, FDR = 1.18e-05); protein-lipid complex organization (Count = 5, FDR = 1.18e-05); protein-lipid complex remodeling (Count = 4, FDR = 1.76e-05); plasma lipoprotein particle remodeling (Count = 4, FDR = 1.76e-05); protein-containing complex remodeling (Count = 4, FDR = 1.76e-05); antimicrobial humoral response (Count = 5, FDR = 1.76e-05); humoral immune response (Count = 6, FDR = 1.76e-05); plasma lipoprotein particle assembly (Count = 4, FDR = 1.91e-05).

# 5 Results of the cancer cachexia validation dataset GSE121972

## 5.1 Results for EDL skeletal muscle

The GSE121972 EDL comparison included Control and Tumour bearing samples. Differential expression analysis tested 20,353 genes and identified 787 stringent differentially expressed genes, including 390 upregulated and 397 downregulated genes. The top differentially expressed genes included Pgm5, Kcnb1, C4a, Osmr, Pld5, Cebpd, Ddit4, Slc10a6, Tmem176b, and Serpina3n, reflecting transcriptional alterations related to muscle structure, inflammatory responses, metabolic stress, and cachexia.

| Gene | logFC | P value | FDR |
|---|---|---|---|
| Pgm5 | 2.237 | 7.86e-12 | 5.44e-08 |
| Kcnb1 | -1.584 | 1.17e-11 | 5.44e-08 |
| C4a | 2.579 | 1.21e-11 | 5.44e-08 |
| Osmr | 1.852 | 1.24e-11 | 5.44e-08 |
| Pld5 | -2.313 | 1.34e-11 | 5.44e-08 |
| Cebpd | 3.026 | 2.18e-11 | 6.73e-08 |
| Ddit4 | 3.425 | 2.55e-11 | 6.73e-08 |
| Slc10a6 | 2.19 | 2.89e-11 | 6.73e-08 |
| Tmem176b | 1.521 | 2.98e-11 | 6.73e-08 |
| Serpina3n | 5.355 | 3.46e-11 | 7.03e-08 |

GO BP enrichment analysis of the stringent differentially expressed genes in EDL showed significant enrichment of muscle function- and metabolism-related terms, including muscle system process (Count = 50, FDR = 1.25e-06); purine ribonucleotide catabolic process (Count = 27, FDR = 1.25e-06); ribonucleotide catabolic process (Count = 27, FDR = 3.03e-06); purine nucleotide catabolic process (Count = 27, FDR = 3.90e-06); muscle cell differentiation (Count = 49, FDR = 4.36e-06); cellular response to interferon-beta (Count = 14, FDR = 5.22e-06);

purine-containing compound catabolic process (Count = 28, FDR = 5.22e-06); purine nucleoside diphosphate metabolic process (Count = 23, FDR = 5.22e-06).

EDL GSEA further showed coordinated alterations in pathways related to oxidative phosphorylation, muscle contraction, and inflammatory regulation, including oxidative phosphorylation (NES = -2.91, FDR = 2.11e-08); energy derivation by oxidation of organic compounds (NES = -2.11, FDR = 2.11e-08); generation of precursor metabolites and energy (NES = -2.06, FDR = 2.11e-08); regulation of inflammatory response (NES = 1.65, FDR = 7.78e-05); acute inflammatory response (NES = 1.71, FDR = 0.0083); muscle cell proliferation (NES = 1.49, FDR = 0.0127); negative regulation of inflammatory response (NES = 1.58, FDR = 0.0128); positive regulation of smooth muscle cell proliferation (NES = 1.70, FDR = 0.0144).

### 5.2 Results for SOL skeletal muscle

The GSE121972 SOL comparison also tested 20,353 genes and identified 558 stringent differentially expressed genes, including 249 upregulated and 309 downregulated genes. The top differentially expressed genes included Kera, Ifi205, Angptl2, Serpina3n, Lcn2, Nrep, Enc1, AI607873, Il4ra, and Tet1. The SOL results indicated an enhanced inflammatory response in cancer cachexia, together with marked alterations in the mitochondrial respiratory chain, oxidative phosphorylation, and ECM/collagen remodeling pathways.

| Gene | logFC | P value | FDR |
|---|---|---|---|
| Kera | -4.43 | 8.99e-13 | 1.83e-08 |
| Ifi205 | 2.34 | 4.04e-12 | 4.12e-08 |
| Angptl2 | -1.846 | 6.65e-12 | 4.51e-08 |
| Serpina3n | 4.379 | 1.02e-11 | 5.21e-08 |
| Lcn2 | 4.322 | 1.64e-11 | 6.68e-08 |
| Nrep | -3.112 | 2.80e-11 | 8.46e-08 |
| Enc1 | 2.005 | 2.91e-11 | 8.46e-08 |
| AI607873 | 1.881 | 4.37e-11 | 1.11e-07 |
| Il4ra | 1.471 | 6.69e-11 | 1.51e-07 |
| Tet1 | -2.411 | 8.15e-11 | 1.66e-07 |

Representative SOL GSEA terms most closely related to the mechanism examined in this study included mitochondrial respiratory chain complex assembly (NES = -2.50, FDR = 6.32e-08); generation of precursor metabolites and energy (NES = -1.75, FDR = 1.49e-06); extracellular matrix organization (NES = -1.88, FDR = 1.91e-06); energy derivation by oxidation of organic compounds (NES = -1.78, FDR = 2.15e-06); mitochondrial translation (NES = -2.11, FDR = 3.03e-06); proton motive force-driven mitochondrial ATP synthesis (NES = -2.26, FDR = 3.12e-05); oxidative phosphorylation (NES = -2.06, FDR = 3.56e-05); mitochondrial respiratory chain complex I assembly (NES = -2.28, FDR = 4.85e-05); mitochondrial ATP synthesis coupled electron transport (NES = -2.20, FDR = 4.85e-05); collagen fibril organization (NES = -2.13, FDR = 8.12e-05).

In the SOL KEGG GSEA, energy metabolism-related pathways were also markedly altered, including Thermogenesis (NES = -2.20, FDR = 1.76e-08); Diabetic cardiomyopathy (NES = -1.90, FDR = 1.14e-05); Citrate cycle (TCA cycle) (NES = -2.13, FDR = 7.36e-05); Oxidative phosphorylation (NES = -1.95, FDR = 9.92e-05).

## 6 Results of the auxiliary chemotherapy myotoxicity validation dataset GSE77745

GSE77745 was an acute doxorubicin-induced tibialis anterior muscle injury model. Among 20,116 genes tested, only Rfx5 met the thresholds of FDR < 0.05 and |log2FC| > 1, indicating that the stringent differential expression signal was weak and should not be used as the basis for the main conclusions. Its GO enrichment was driven by a single gene and mainly involved terms related to the MHC class II biosynthetic process. GSEA based on the ranked list of all genes showed alterations in mitochondrial translation, mitochondrial gene expression, ECM organization, and the regulation of cell migration, providing auxiliary evidence in the context of chemotherapy-induced myotoxicity.

| Gene | logFC | P value | FDR |
|---|---|---|---|
| Rfx5 | 2.4 | 9.42e-07 | 0.01895 |

| | | | |
|---|---|---|---|
| Taar7a | -2.591 | 8.61e-06 | 0.08664 |
| Dnaic1 | 4.237 | 2.14e-05 | 0.1118 |
| Top1mt | -1.296 | 2.22e-05 | 0.1118 |
| Plch2 | 3.067 | 3.32e-05 | 0.1336 |
| Clec4g | -3.22 | 4.64e-05 | 0.1556 |
| Nfasc | -3.099 | 1.07e-04 | 0.3067 |
| Cd44 | -2.809 | 1.72e-04 | 0.4026 |
| Sigirr | -1.536 | 2.30e-04 | 0.4026 |
| 1700027A07Rik | 2.812 | 2.48e-04 | 0.4026 |

Representative GSE77745 GSEA terms included mitochondrial translation (NES = 2.30, FDR = 2.49e-06); mitochondrial gene expression (NES = 2.14, FDR = 3.70e-05); negative regulation of cell migration (NES = -1.76, FDR = 9.50e-04); extracellular structure organization (NES = -1.70, FDR = 0.0041); extracellular matrix organization (NES = -1.69, FDR = 0.0041); negative regulation of blood vessel endothelial cell migration (NES = -2.13, FDR = 0.0286); smooth muscle cell differentiation (NES = -1.87, FDR = 0.0297); negative regulation of cell migration involved in sprouting angiogenesis (NES = -2.09, FDR = 0.0348).

## 7 Key gene analysis

To inform the selection of markers for subsequent wet-laboratory experiments, we focused on Akt1, Foxo3, Trim63, Fbxo32, Ppargc1a, Il6, Tnf, Col1a1, Col3a1, Mstn, Nfkb1, Stat3, Myog, and Myod1. Akt1, Foxo3, Trim63, Fbxo32, and Ppargc1a were detected in the primary GSE80473 dataset. However, Trim63, Fbxo32, and Ppargc1a showed only trends or nominal P-value signals and none remained significant after FDR correction; Il6 and Tnf were not detected using exact gene-symbol matching. Therefore, GSE80473 could not be used to demonstrate significant changes in these genes and instead served as a source of pathway-level evidence and candidate markers. In the GSE121972 validation dataset, Akt1, Foxo3, Trim63, Fbxo32, Ppargc1a, Mstn, Stat3, and Myog were significantly altered in EDL, whereas Foxo3, Trim63, Fbxo32, Stat3, and Myog were significantly altered in SOL. These findings supported abnormalities in molecules related to FoxO/UPS, inflammation/STAT signaling, and muscle differentiation during cachexia-associated skeletal muscle wasting.

| **Dataset** | **Gene** | **Status** | **logFC** | **P value** | **FDR** |
|---|---|---|---|---|---|
| GSE80473_local | Akt1 | present | 0.07471 | 0.6291 | 0.8907 |
| GSE80473_local | Foxo3 | present | -0.3361 | 0.1568 | 0.6433 |
| GSE80473_local | Trim63 | present | -0.6748 | 0.00281 | 0.3112 |
| GSE80473_local | Fbxo32 | present | -0.6174 | 0.02065 | 0.452 |
| GSE80473_local | Ppargc1a | present | -0.7475 | 0.04122 | 0.5031 |
| GSE80473_local | Il6 | missing | | | |
| GSE80473_local | Tnf | missing | | | |
| GSE80473_local | Stat3 | present | -0.1756 | 0.4831 | 0.8316 |
| GSE80473_local | Myog | present | 0.3807 | 0.08225 | 0.5579 |
| GSE80473_local | Myod1 | present | 0.5393 | 0.02903 | 0.4739 |
| GSE80473_depositor | Akt1 | present | 0.07383 | 0.7865 | 0.9995 |
| GSE80473_depositor | Foxo3 | present | -0.3337 | 0.4474 | 0.9995 |
| GSE80473_depositor | Trim63 | present | -0.6765 | 0.02502 | 0.9774 |
| GSE80473_depositor | Fbxo32 | present | -0.6191 | 0.07134 | 0.9995 |
| GSE80473_depositor | Ppargc1a | present | -0.7464 | 0.03507 | 0.9995 |
| GSE80473_depositor | Il6 | missing | | | |
| GSE80473_depositor | Tnf | missing | | | |
| GSE80473_depositor | Stat3 | present | -0.1766 | 0.6401 | 0.9995 |
| GSE80473_depositor | Myog | present | 0.3795 | 0.2843 | 0.9995 |
| GSE80473_depositor | Myod1 | present | 0.5383 | 0.09095 | 0.9995 |
| GSE77745 | Akt1 | present | 0.7682 | 0.5195 | 0.9021 |
| GSE77745 | Foxo3 | present | -0.1526 | 0.8924 | 1 |
| GSE77745 | Trim63 | present | -0.4178 | 0.09983 | 0.8006 |
| GSE77745 | Fbxo32 | present | -0.7378 | 0.2328 | 0.8006 |
| GSE77745 | Ppargc1a | present | 0.3513 | 0.2817 | 0.8006 |
| GSE77745 | Il6 | present | 0.434 | 0.4892 | 0.8848 |
| GSE77745 | Tnf | present | -0.9508 | 0.2155 | 0.8006 |
| GSE77745 | Stat3 | present | -0.1878 | 0.3872 | 0.8232 |
| GSE77745 | Myog | present | 0.02938 | 0.9385 | 1 |
| GSE77745 | Myod1 | present | 0.0238 | 0.9511 | 1 |
| GSE121972_EDL | Akt1 | present | 1.186 | 2.93e-06 | 9.03e-05 |
| GSE121972_EDL | Foxo3 | present | 1.208 | 9.22e-08 | 1.07e-05 |

| | | | | | |
|---|---|---|---|---|---|
| GSE121972_EDL | Trim63 | present | 2.658 | 9.65e-10 | 5.61e-07 |
| GSE121972_EDL | Fbxo32 | present | 2.211 | 1.42e-09 | 7.40e-07 |
| GSE121972_EDL | Ppargc1a | present | 0.7071 | 0.005336 | 0.0218 |
| GSE121972_EDL | Il6 | present | 0.01353 | 0.913 | 0.9538 |
| GSE121972_EDL | Tnf | present | -0.3438 | 0.005306 | 0.02172 |
| GSE121972_EDL | Stat3 | present | 1.385 | 7.06e-10 | 4.79e-07 |
| GSE121972_EDL | Myog | present | 1.044 | 0.001026 | 0.00583 |
| GSE121972_EDL | Myod1 | present | -0.9409 | 0.002748 | 0.01276 |
| GSE121972_SOL | Akt1 | present | 0.1908 | 0.1057 | 0.2609 |
| GSE121972_SOL | Foxo3 | present | 1.038 | 9.13e-08 | 1.28e-05 |
| GSE121972_SOL | Trim63 | present | 1.544 | 3.72e-08 | 7.24e-06 |
| GSE121972_SOL | Fbxo32 | present | 0.6325 | 6.82e-05 | 0.001175 |
| GSE121972_SOL | Ppargc1a | present | 0.5975 | 0.1052 | 0.2601 |
| GSE121972_SOL | Il6 | present | 0.1553 | 0.2504 | 0.4614 |
| GSE121972_SOL | Tnf | present | -0.8482 | 0.002586 | 0.01692 |
| GSE121972_SOL | Stat3 | present | 0.9076 | 2.12e-07 | 2.27e-05 |
| GSE121972_SOL | Myog | present | 1.34 | 6.50e-04 | 0.005999 |
| GSE121972_SOL | Myod1 | present | -0.5523 | 0.008753 | 0.04247 |

## 8 Summary of the GEO results

In summary, the GEO public transcriptomic analysis indicated altered transcriptional programs related to fatty acid oxidation, energy metabolism, and muscle contraction in FOLFIRI/chemotherapy-associated skeletal muscle injury. The cancer cachexia validation dataset further showed abnormalities in FoxO/UPS, inflammation/STAT signaling, mitochondrial oxidative phosphorylation, and ECM/collagen remodeling pathways. These findings provided public-data support for the subsequent assessment of Akt/FoxO3a/MuRF1/Atrogin-1/PGC-1α. The interpretive boundary must be strictly defined: the GEO analysis was used to explore altered pathways and candidate markers in chemotherapy- or cachexia-associated skeletal muscle injury. It was not used to demonstrate the direct efficacy of CPhGs or to predict their direct drug targets.

# 9 Appendix of all figures

This appendix contains all 52 GEO analysis figures from the scheduler/results/figures directory. The figures are ordered by dataset and filename and include quality-control plots, differential expression plots, enrichment plots, GSEA plots, key gene expression plots, and the depositor-provided edgeR sensitivity analysis plots for GSE80473.

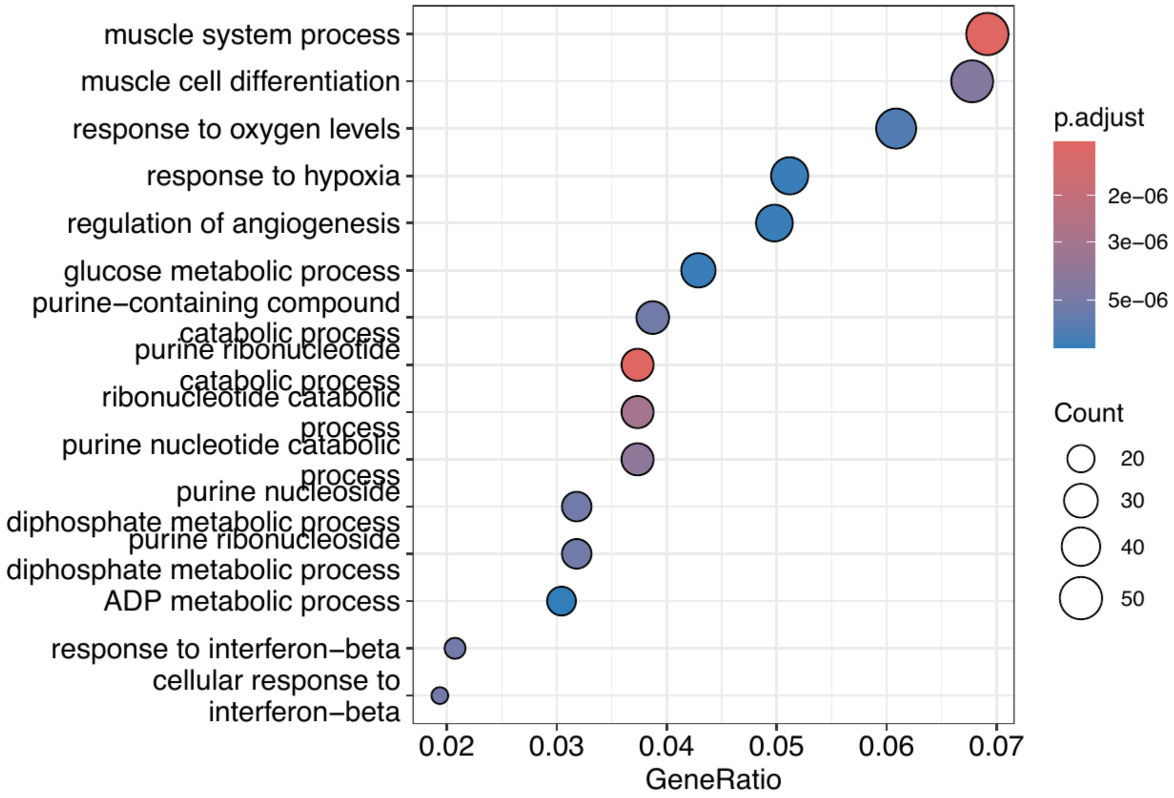


**Figure S1**. GSE121972 EDL Tumour bearing versus Control: GO Biological Process enrichment dot plot.

Original figure file: GSE121972_EDL_Tumour_vs_Control_GO_BP_dotplot.pdf

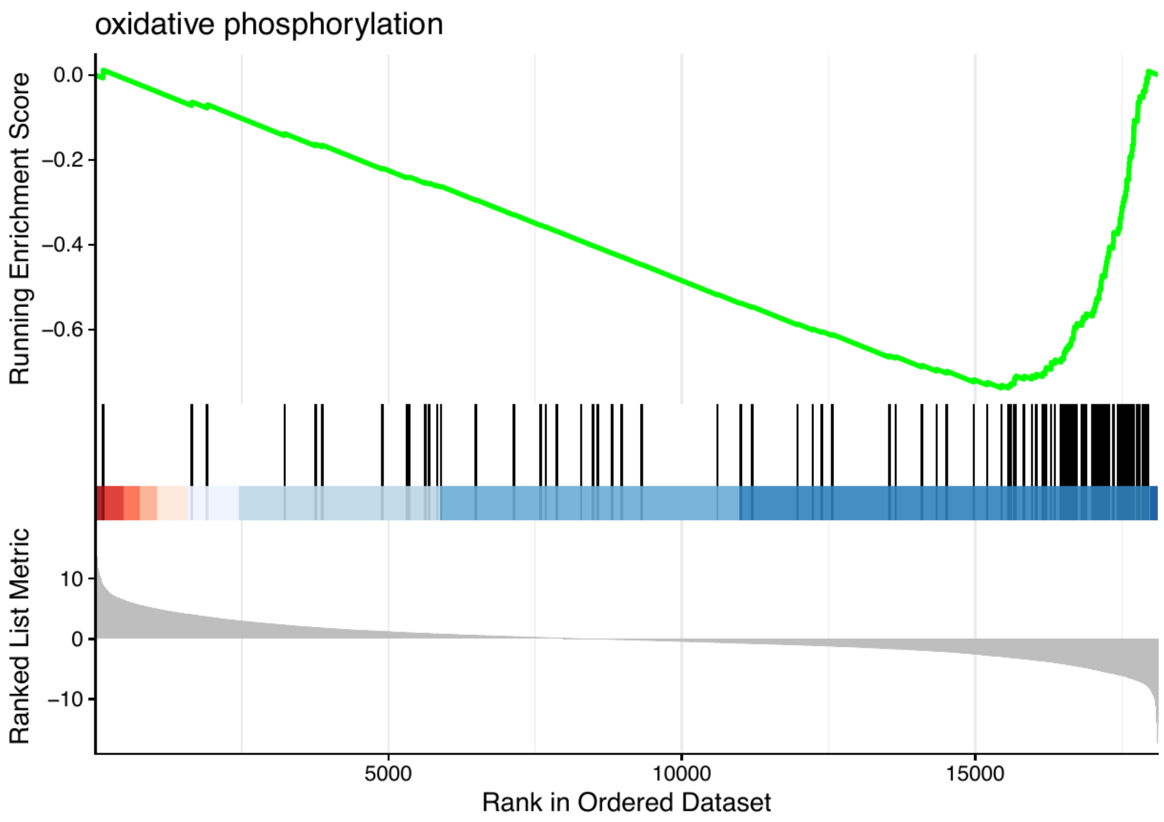


**Figure S2.** GSE121972 EDL Tumour bearing versus Control: GSEA curves for energy metabolism-related GO BP terms.

Original figure file: GSE121972_EDL_Tumour_vs_Control_GSEA_GO_BP_energy.pdf

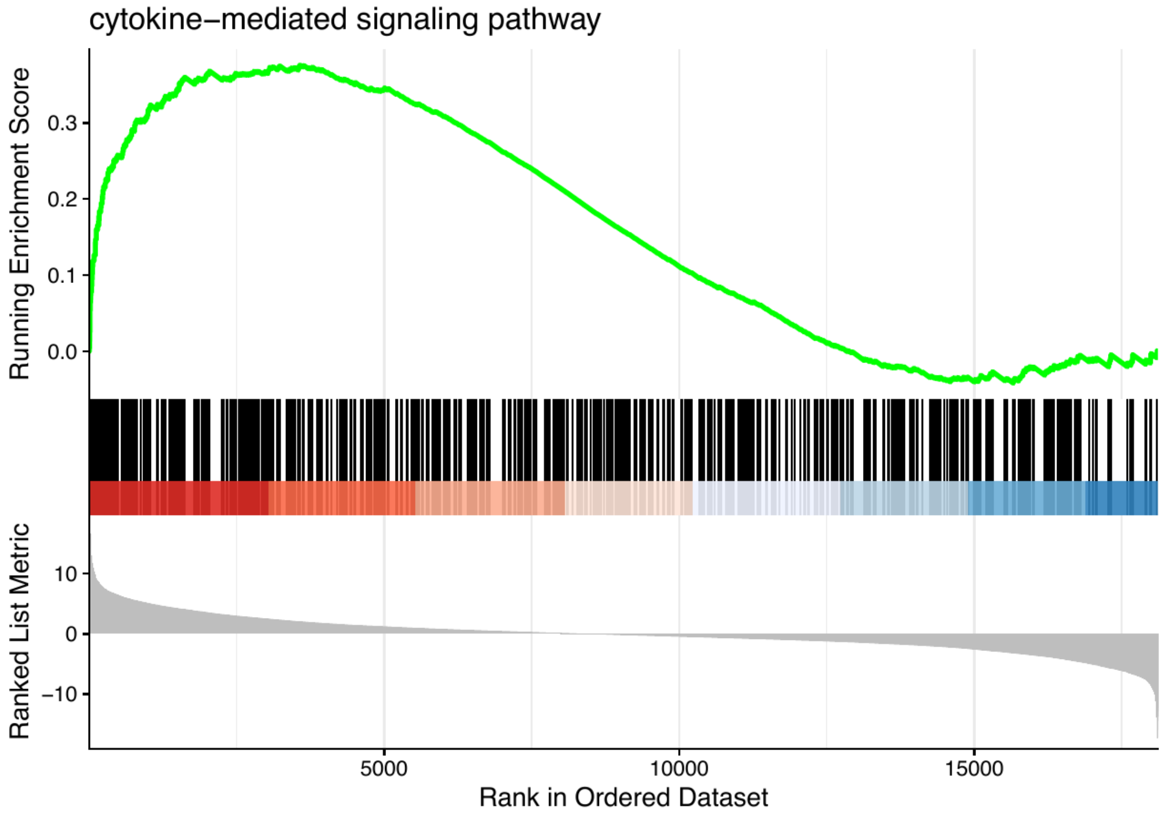


**Figure S3.** GSE121972 EDL Tumour bearing versus Control: GSEA curves for inflammatory response-related GO BP terms.

Original figure file: GSE121972_EDL_Tumour_vs_Control_GSEA_GO_BP_inflammatory.pdf

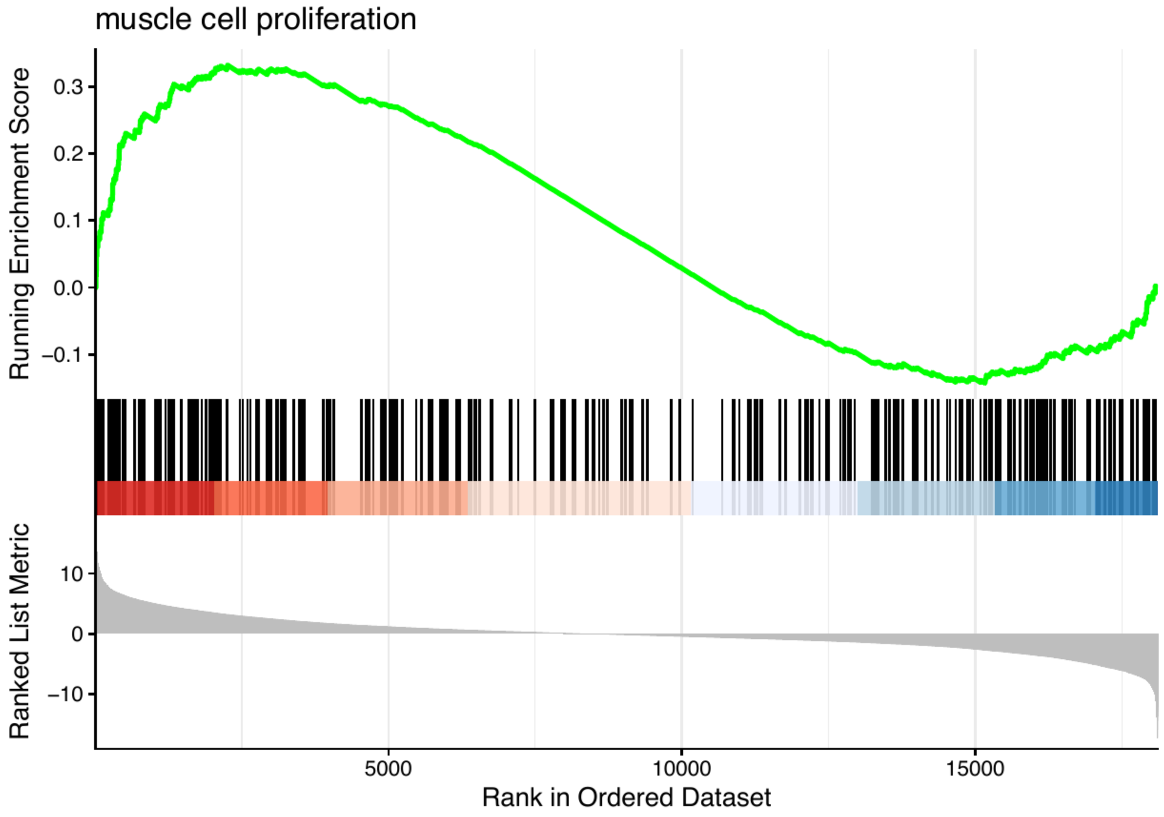


**Figure S4.** GSE121972 EDL Tumour bearing versus Control: GSEA curves for muscle function-related GO BP terms.

Original figure file: GSE121972_EDL_Tumour_vs_Control_GSEA_GO_BP_muscle.pdf

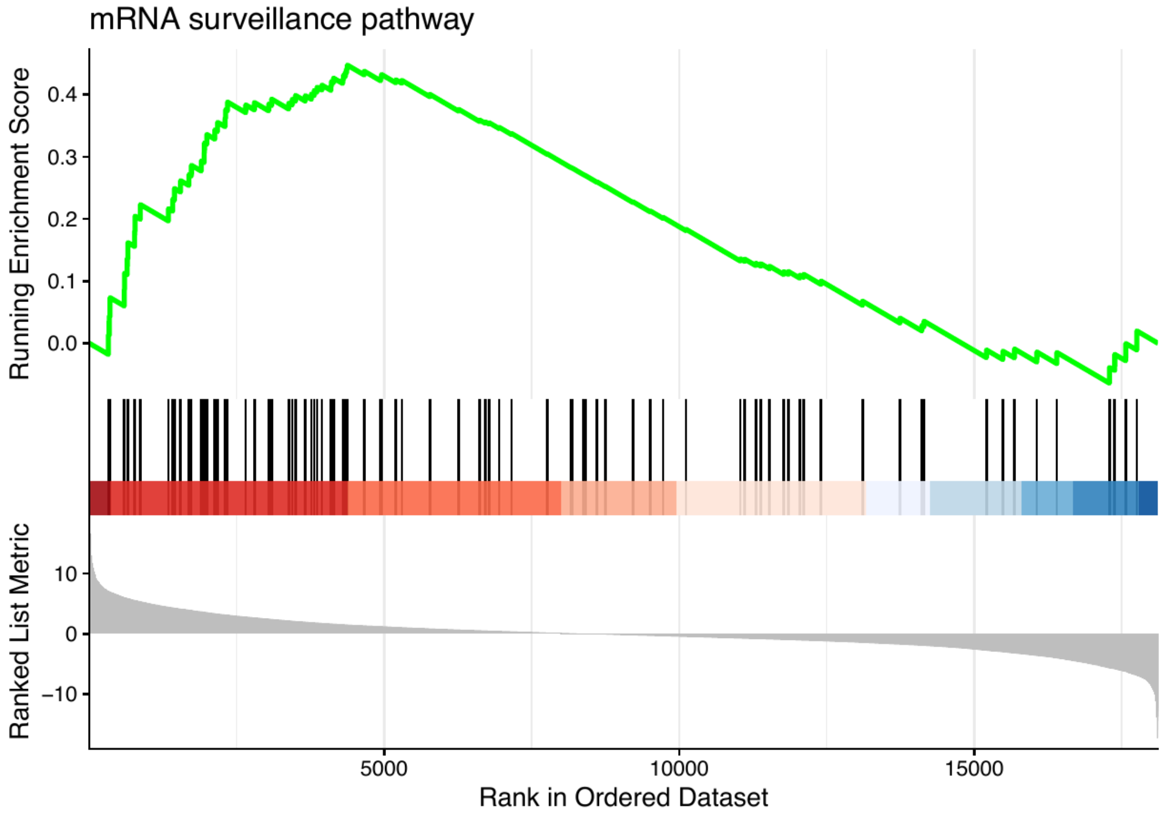


Figure S5. GSE121972 EDL Tumour bearing versus Control: GSEA curves for KEGG cytokine-related pathways.

Original figure file: GSE121972_EDL_Tumour_vs_Control_GSEA_KEGG_cytokine.pdf

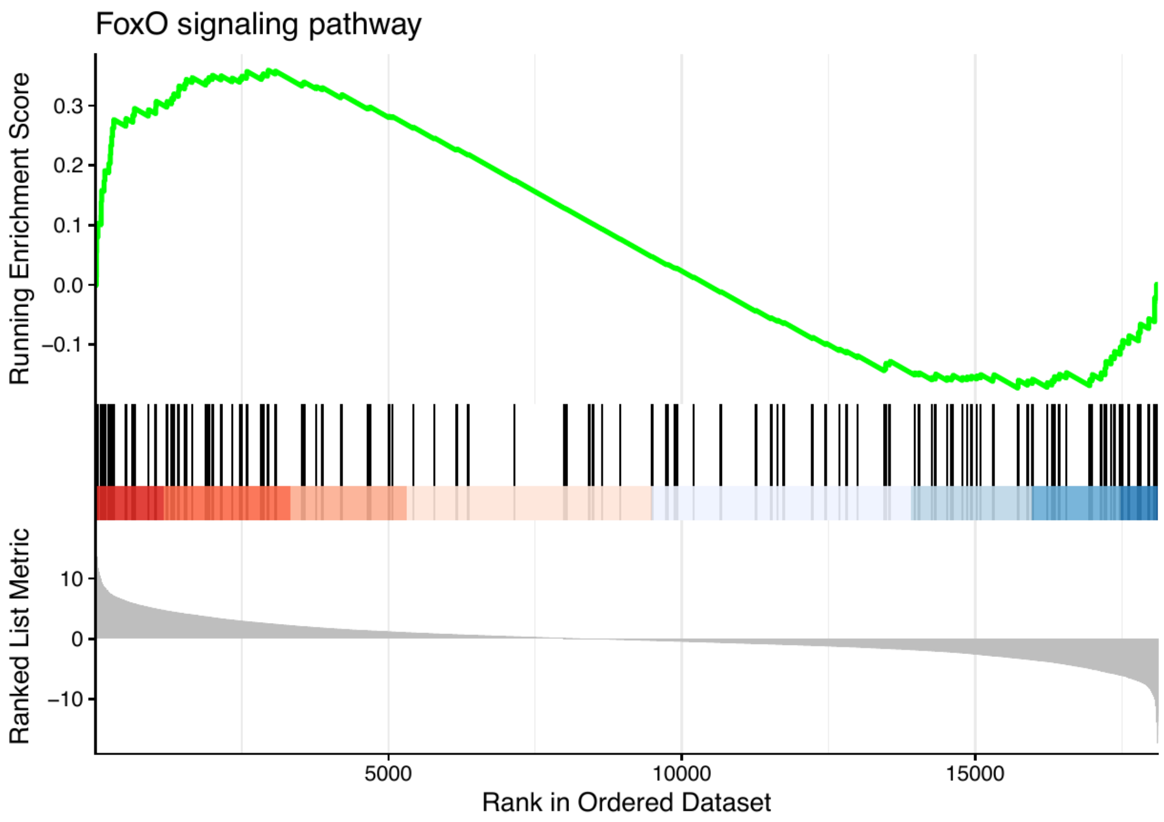


**Figure S6.** GSE121972 EDL Tumour bearing versus Control: GSEA curve for the KEGG FoxO signaling pathway.

Original figure file: GSE121972_EDL_Tumour_vs_Control_GSEA_KEGG_foxo.pdf

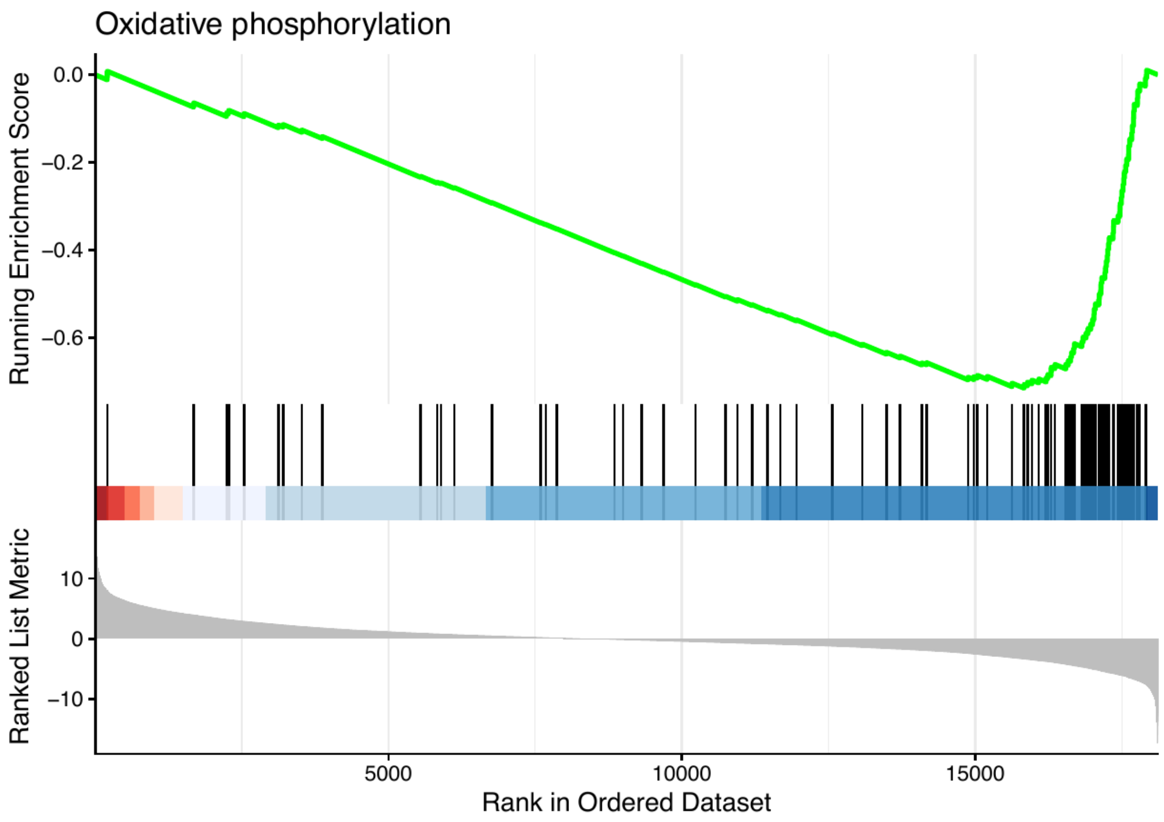


**Figure S7.** GSE121972 EDL Tumour bearing versus Control: GSEA curve for the KEGG oxidative phosphorylation pathway.

Original figure file: GSE121972_EDL_Tumour_vs_Control_GSEA_KEGG_oxidative_phosphorylation.pdf

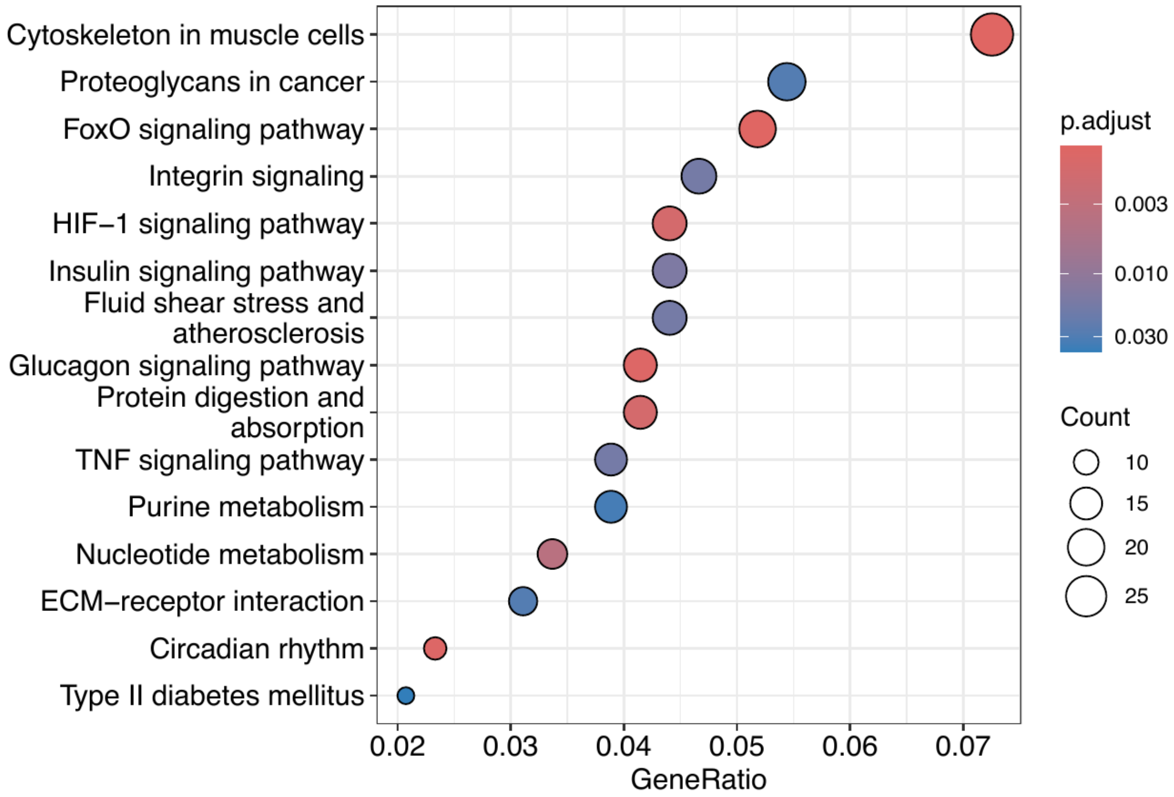


**Figure S8.** GSE121972 EDL Tumour bearing versus Control: KEGG enrichment dot plot.

Original figure file: GSE121972_EDL_Tumour_vs_Control_KEGG_dotplot.pdf

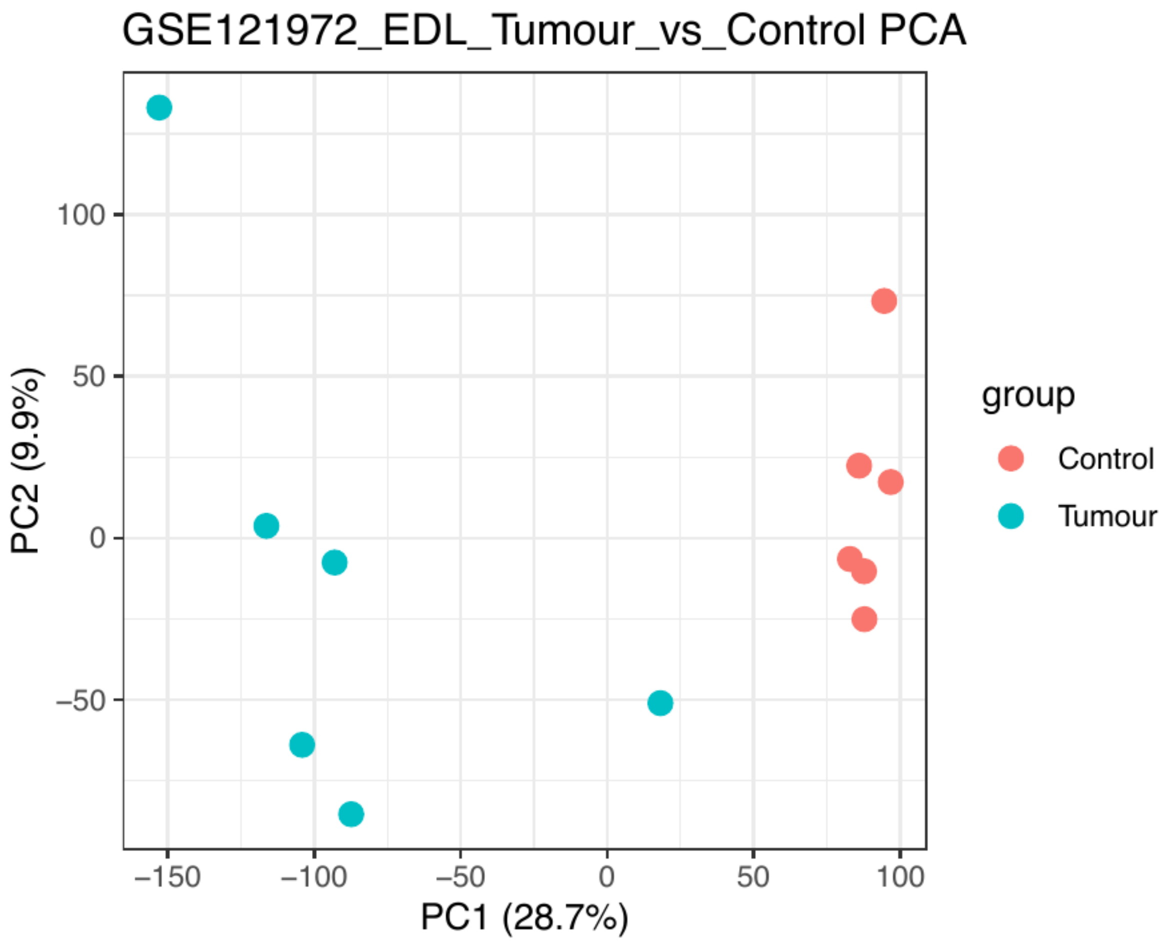


**Figure S9.** GSE121972 EDL Tumour bearing versus Control: principal component analysis plot.

Original figure file: GSE121972_EDL_Tumour_vs_Control_QC_PCA.pdf

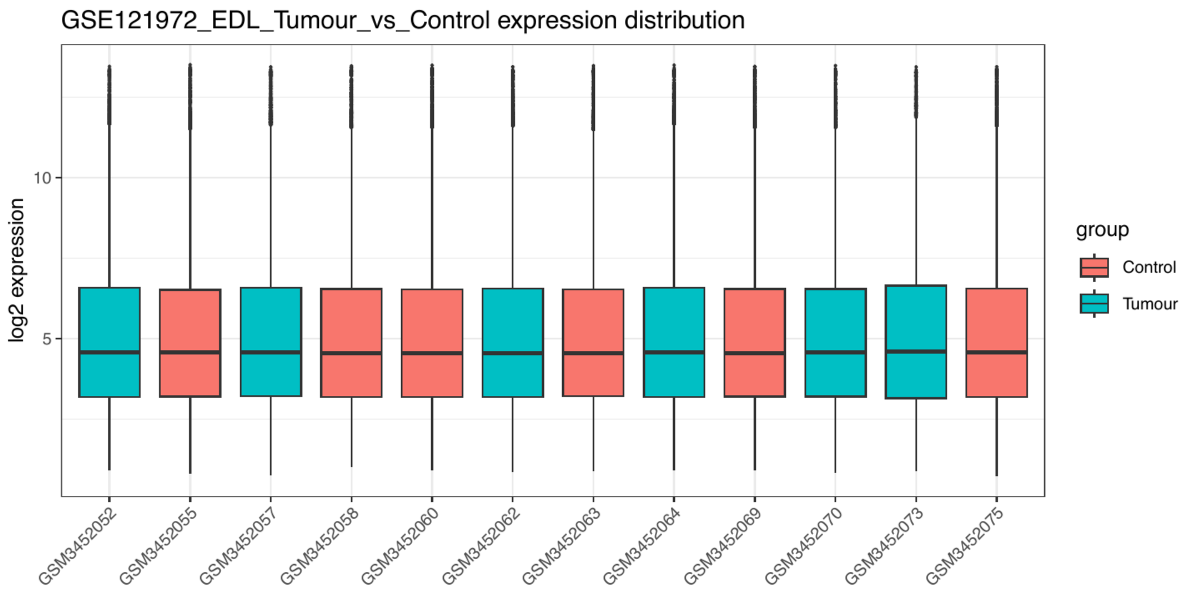


**Figure S10.** GSE121972 EDL Tumour bearing versus Control: expression distribution boxplot.

Original figure file: GSE121972_EDL_Tumour_vs_Control_QC_boxplot.pdf

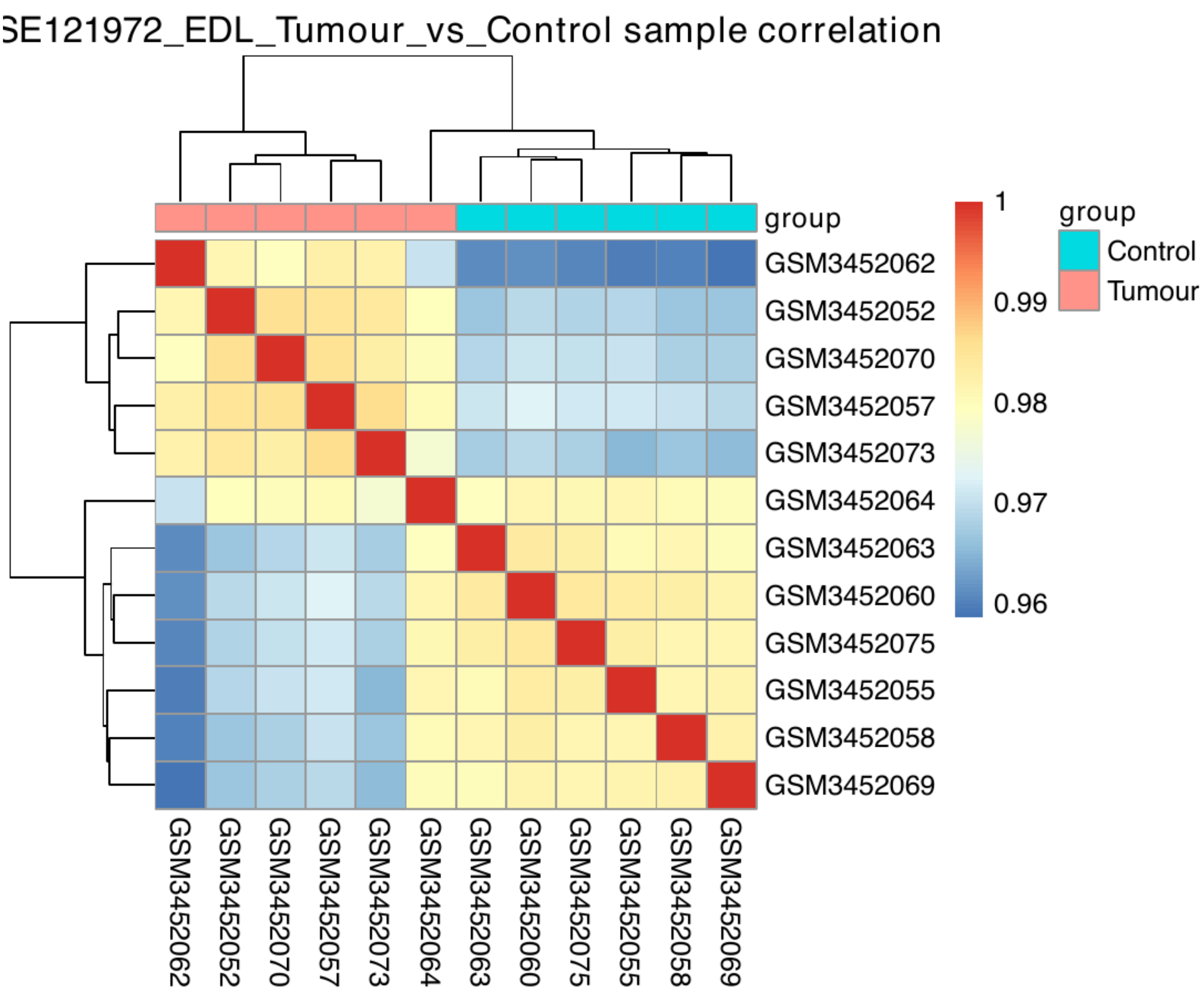


**Figure S11.** GSE121972 EDL Tumour bearing versus Control: sample correlation heatmap.

Original figure file: GSE121972_EDL_Tumour_vs_Control_QC_sample_correlation.pdf

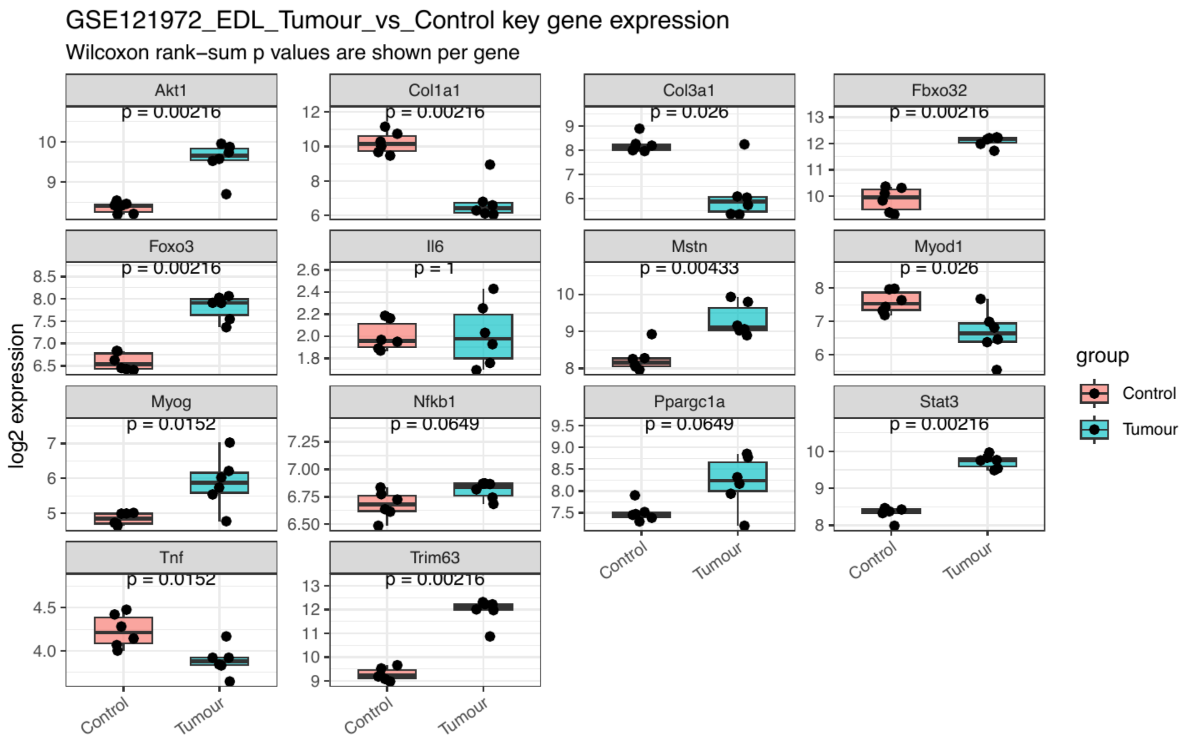


**Figure S12.** GSE121972 EDL Tumour bearing versus Control: key gene expression plot.

Original figure file: GSE121972_EDL_Tumour_vs_Control_key_gene_expression.pdf

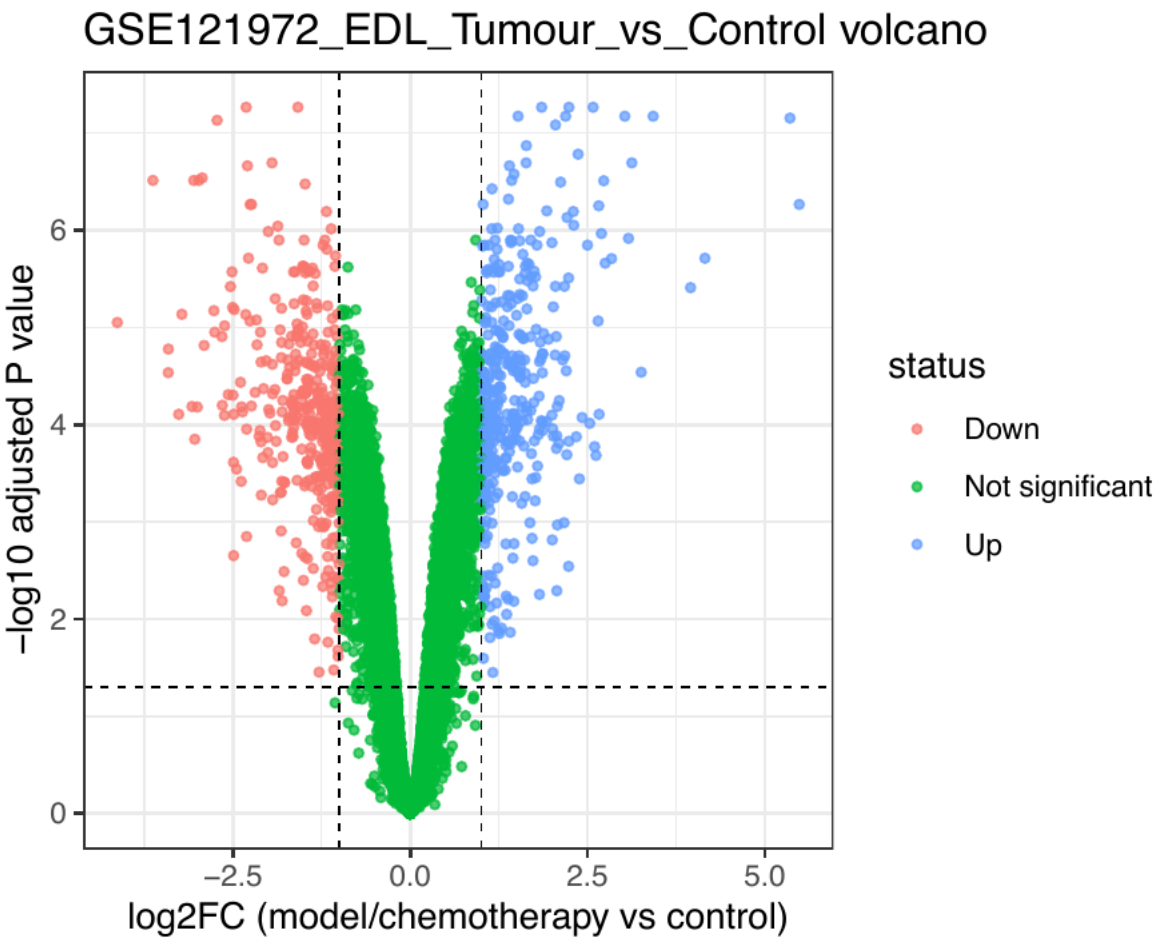


**Figure S13.** GSE121972 EDL Tumour bearing versus Control: differential expression volcano plot.

Original figure file: GSE121972_EDL_Tumour_vs_Control_volcano.pdf

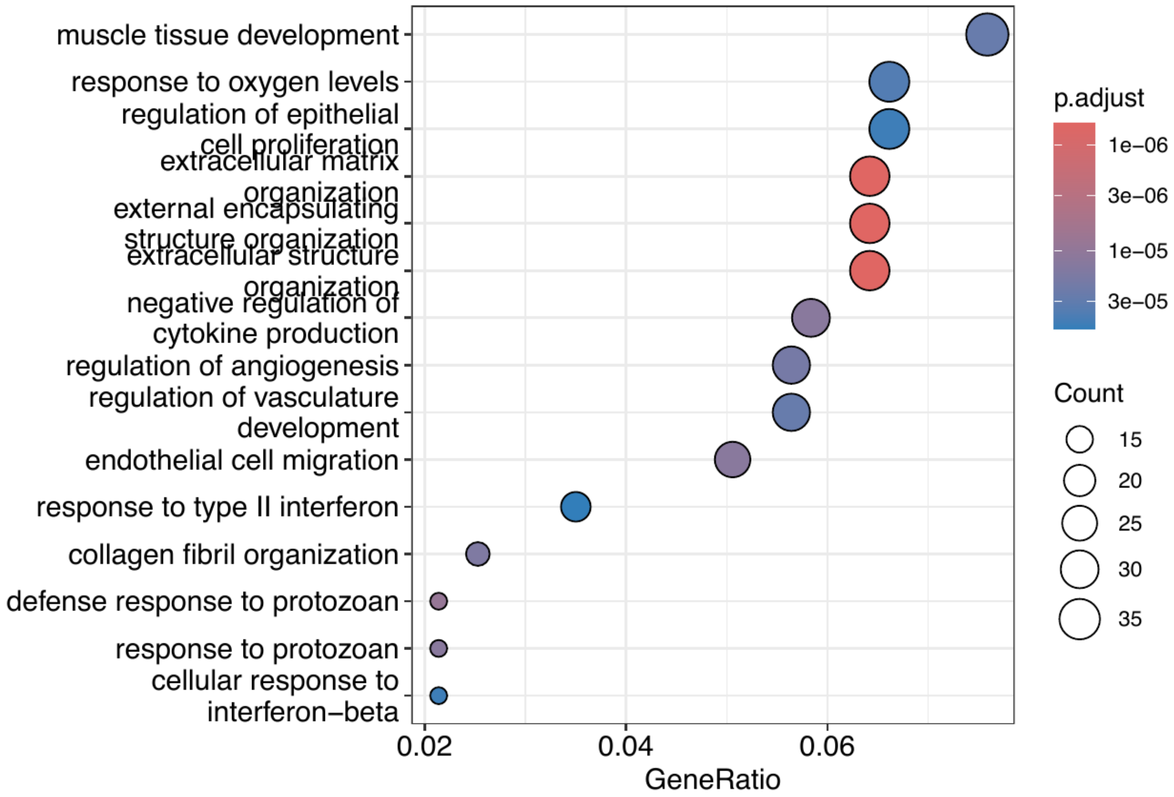


**Figure S14.** GSE121972 SOL Tumour bearing versus Control: GO Biological Process enrichment dot plot.

Original figure file: GSE121972_SOL_Tumour_vs_Control_GO_BP_dotplot.pdf

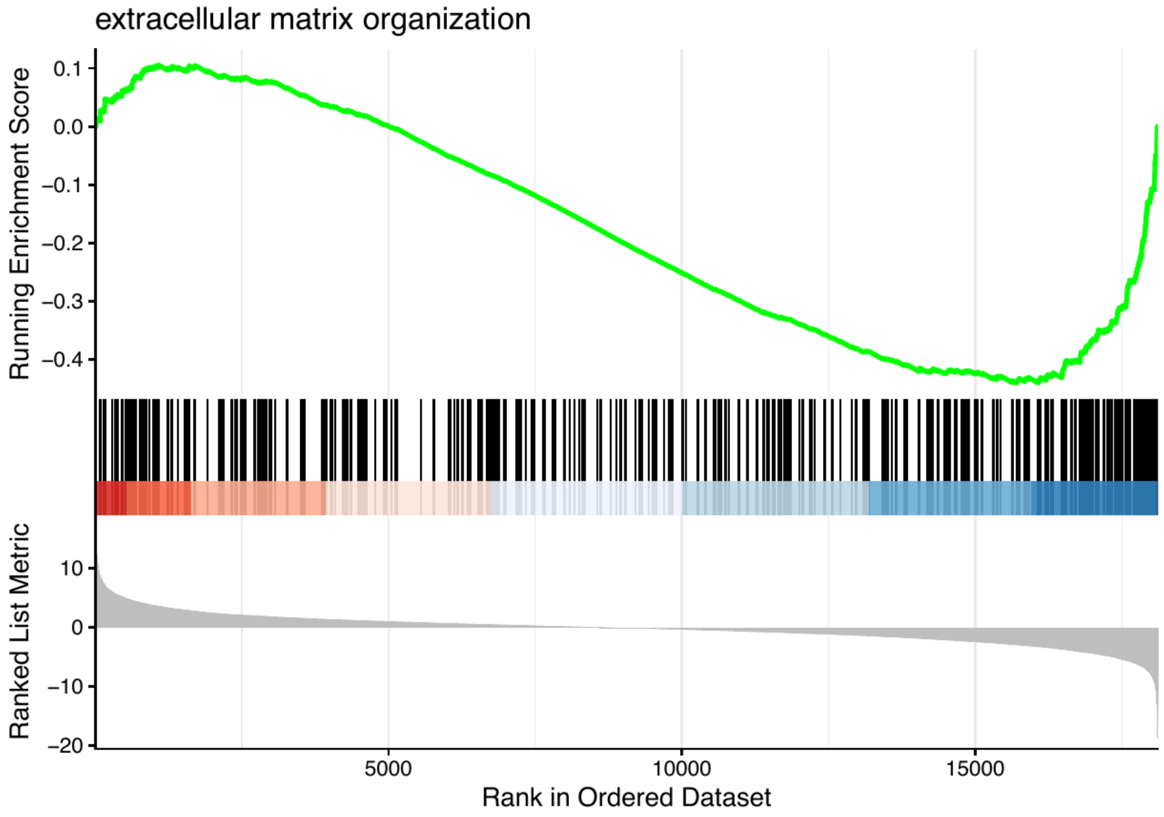


**Figure S15.** GSE121972 SOL Tumour bearing versus Control: GSEA curves for extracellular matrix-related GO BP terms.

Original figure file: GSE121972_SOL_Tumour_vs_Control_GSEA_GO_BP_ecm.pdf

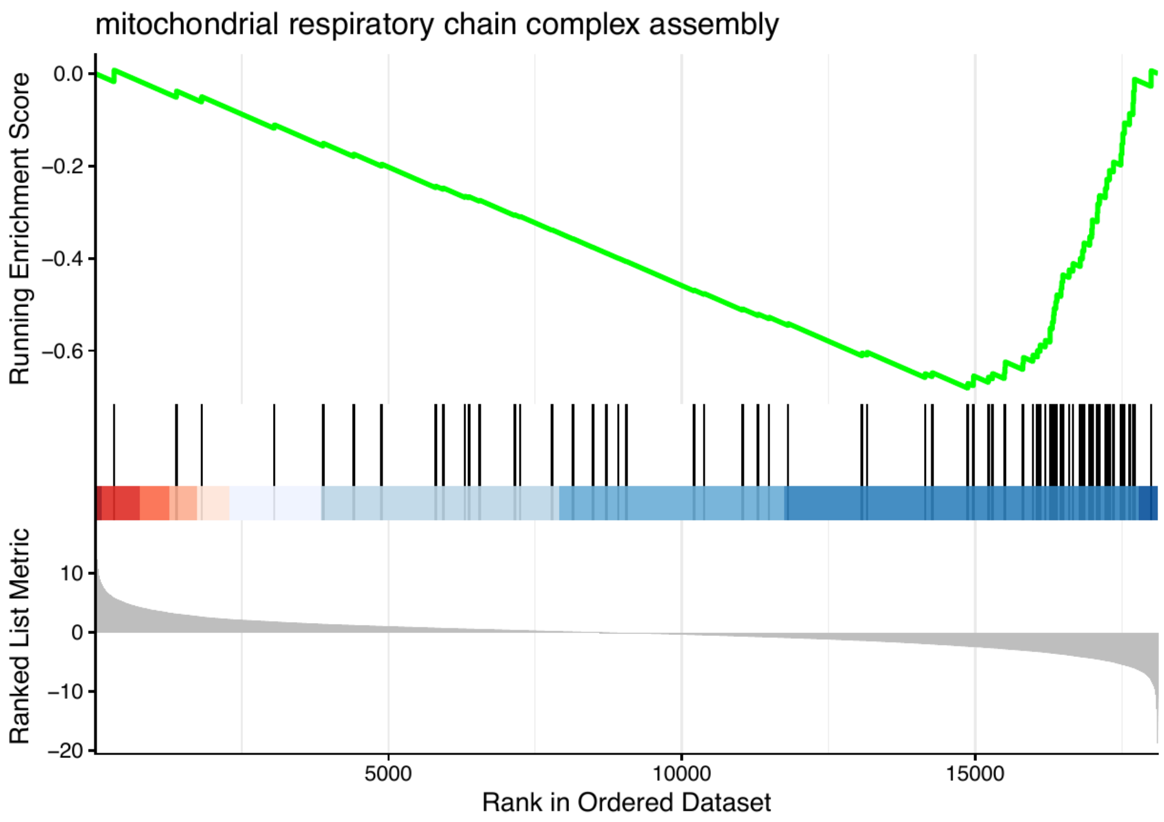


**Figure S16.** GSE121972 SOL Tumour bearing versus Control: GSEA curves for energy metabolism-related GO BP terms.

Original figure file: GSE121972_SOL_Tumour_vs_Control_GSEA_GO_BP_energy.pdf

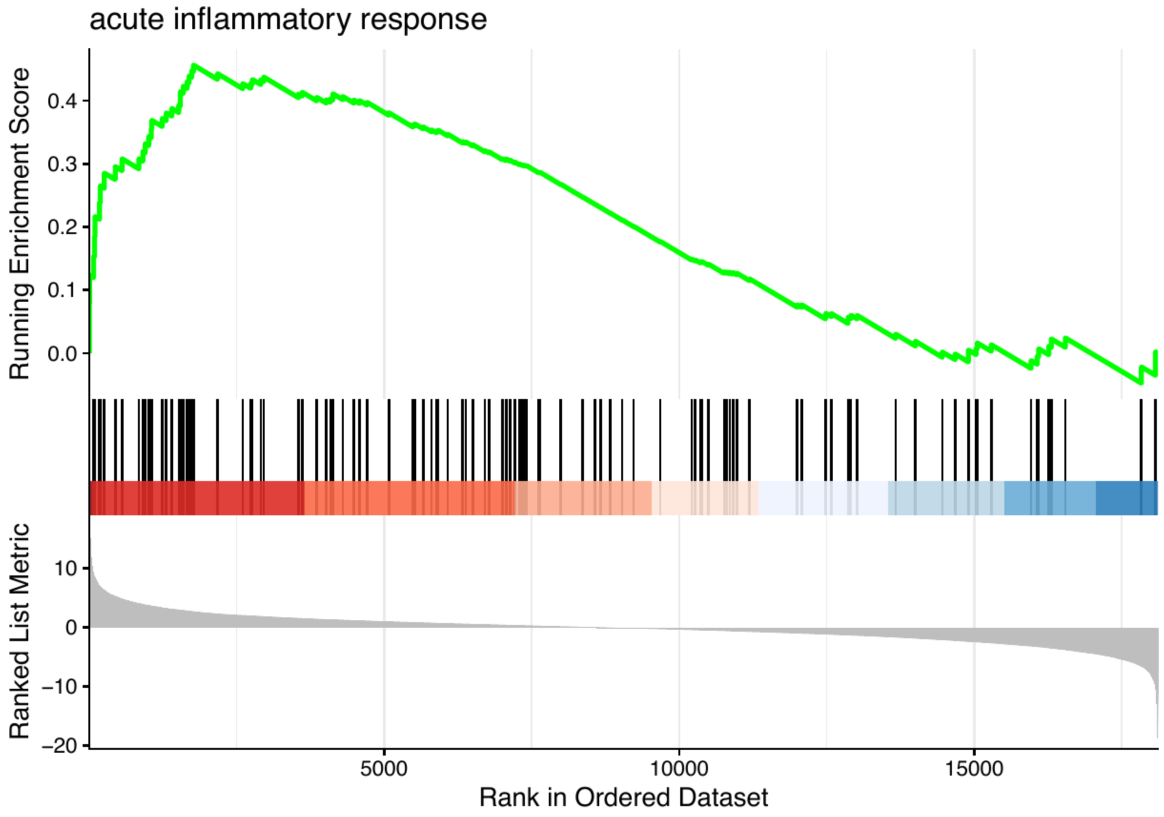


**Figure S17.** GSE121972 SOL Tumour bearing versus Control: GSEA curves for inflammatory response-related GO BP terms.

Original figure file: GSE121972_SOL_Tumour_vs_Control_GSEA_GO_BP_inflammatory.pdf

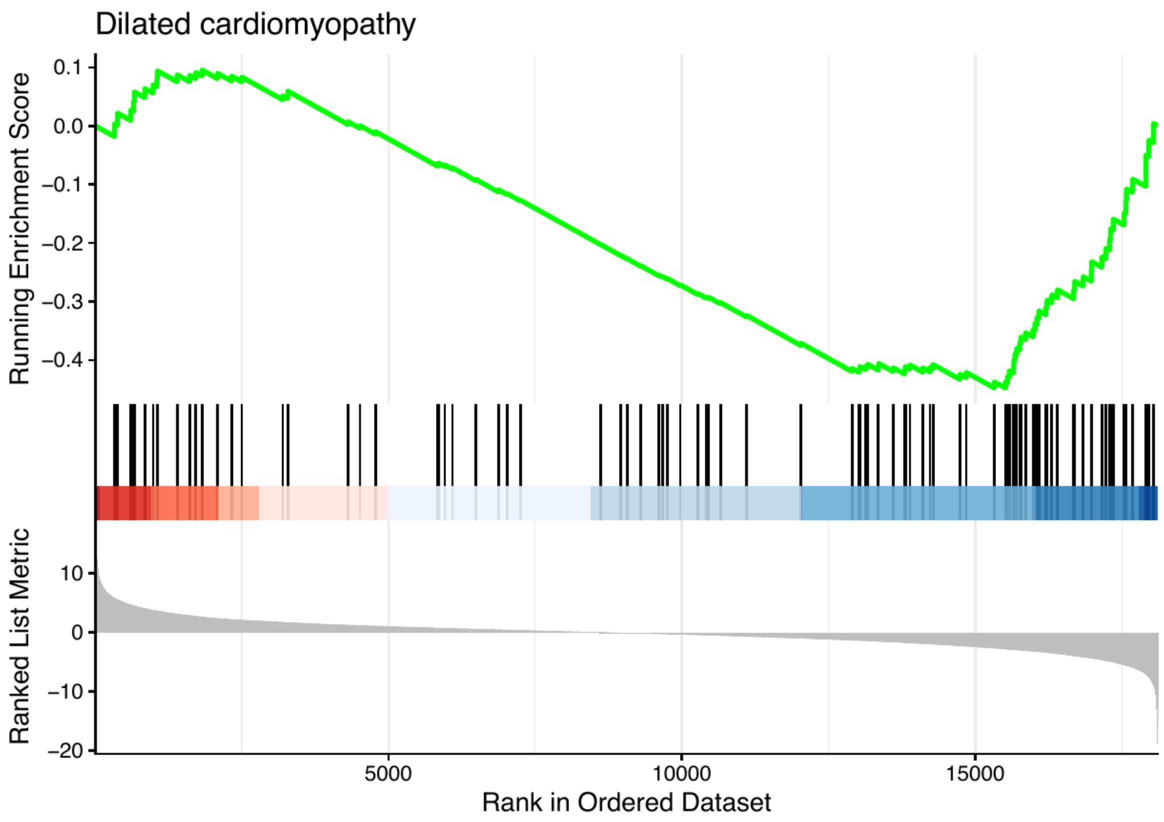


**Figure S18.** GSE121972 SOL Tumour bearing versus Control: GSEA curves for KEGG cytokine-related pathways.

Original figure file: GSE121972_SOL_Tumour_vs_Control_GSEA_KEGG_cytokine.pdf

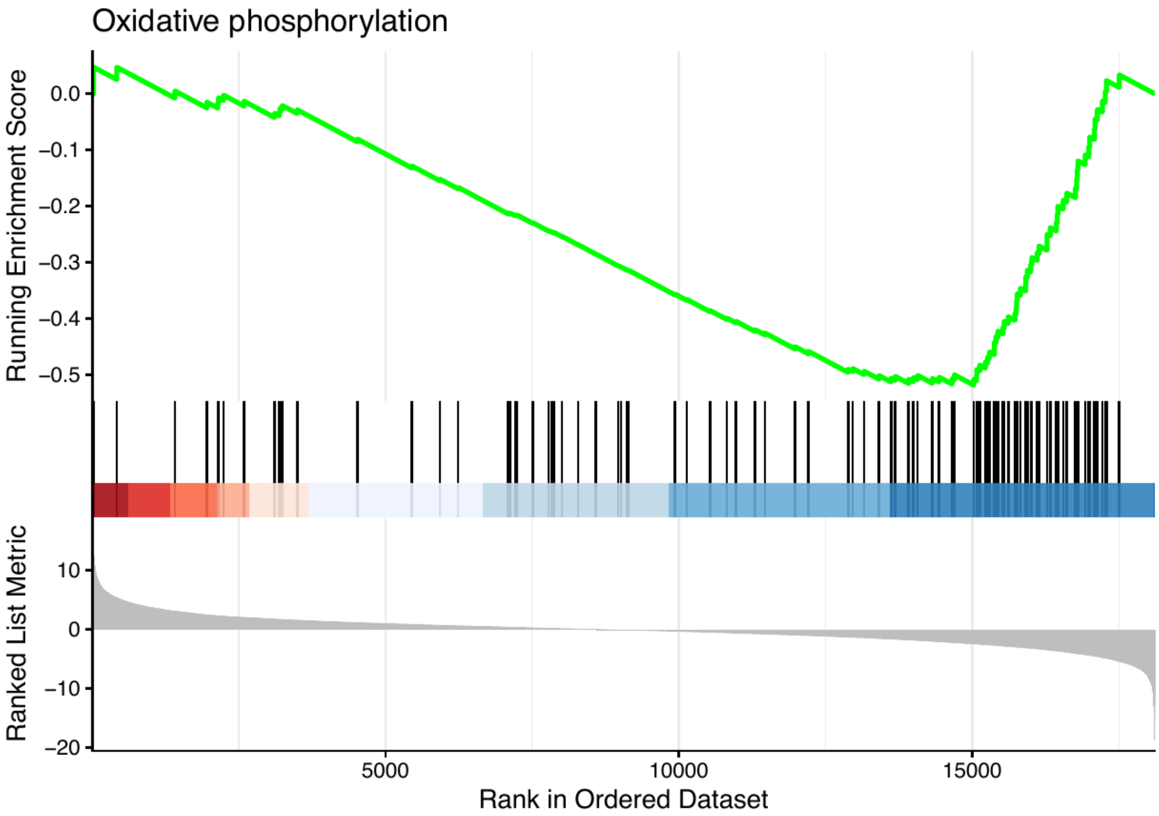


**Figure S19.** GSE121972 SOL Tumour bearing versus Control: GSEA curve for the KEGG oxidative phosphorylation pathway.

Original figure file: GSE121972_SOL_Tumour_vs_Control_GSEA_KEGG_oxidative_phosphorylation.pdf

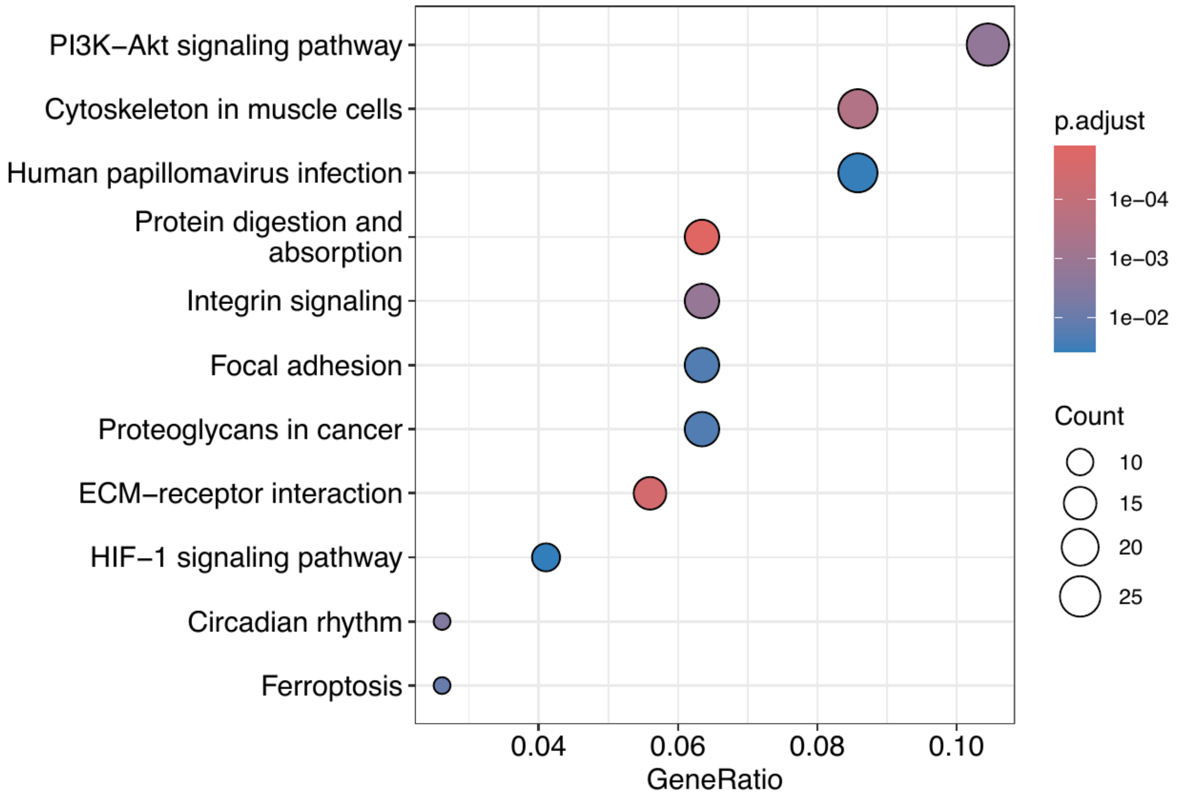


**Figure S20.** GSE121972 SOL Tumour bearing versus Control: KEGG enrichment dot plot.

Original figure file: GSE121972_SOL_Tumour_vs_Control_KEGG_dotplot.pdf

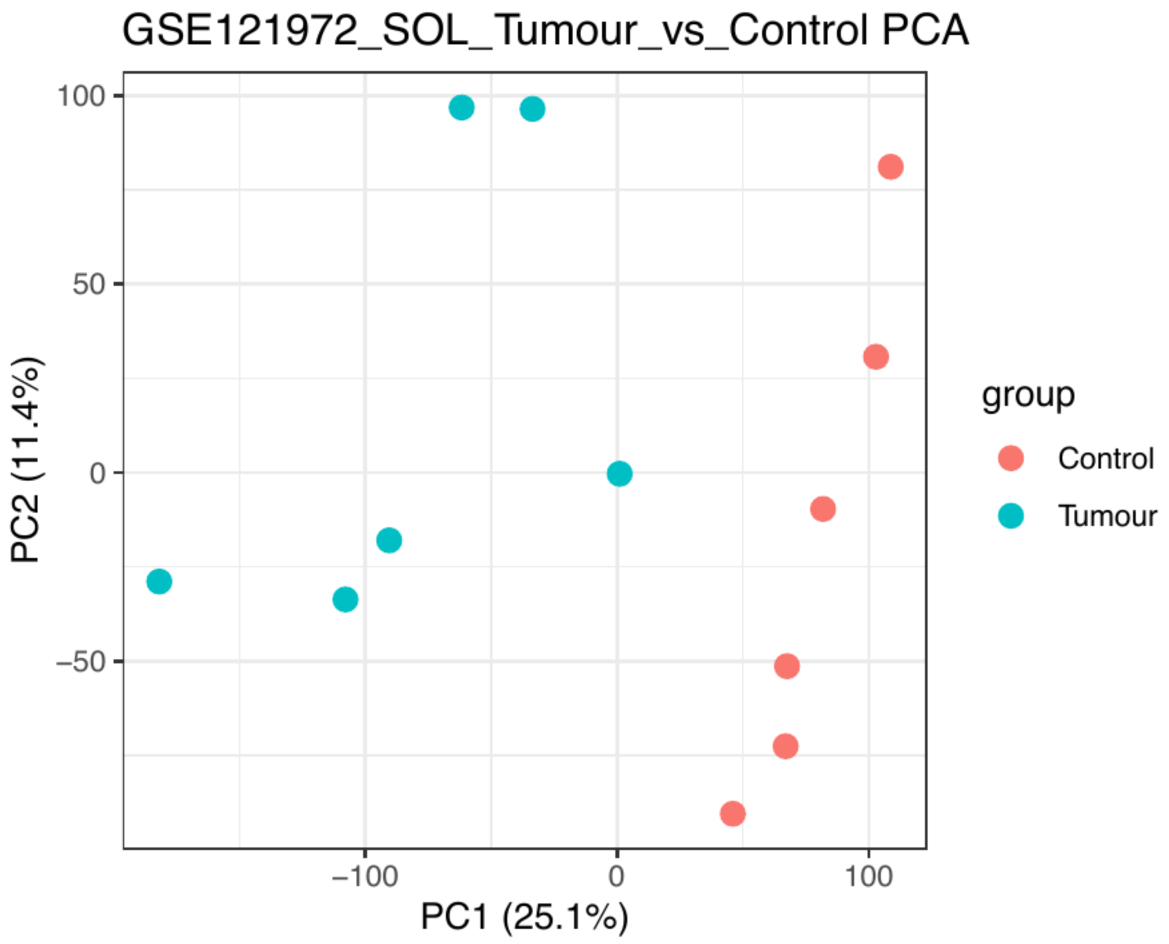


**Figure S21.** GSE121972 SOL Tumour bearing versus Control: principal component analysis plot.

Original figure file: GSE121972_SOL_Tumour_vs_Control_QC_PCA.pdf

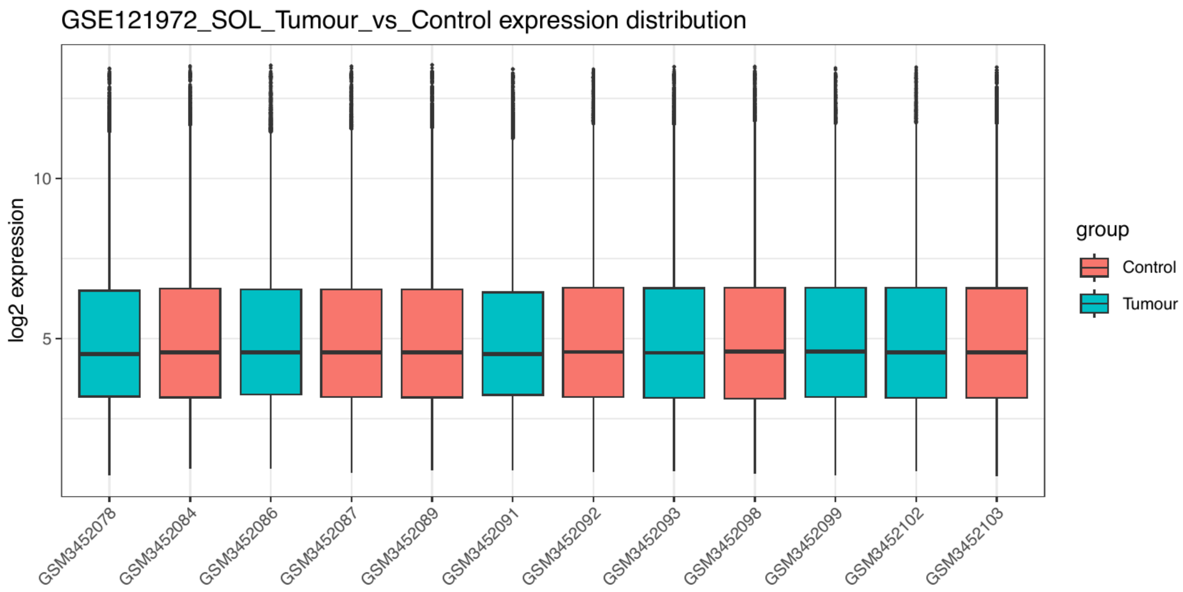


**Figure S22.** GSE121972 SOL Tumour bearing versus Control: expression distribution boxplot.

Original figure file: GSE121972_SOL_Tumour_vs_Control_QC_boxplot.pdf

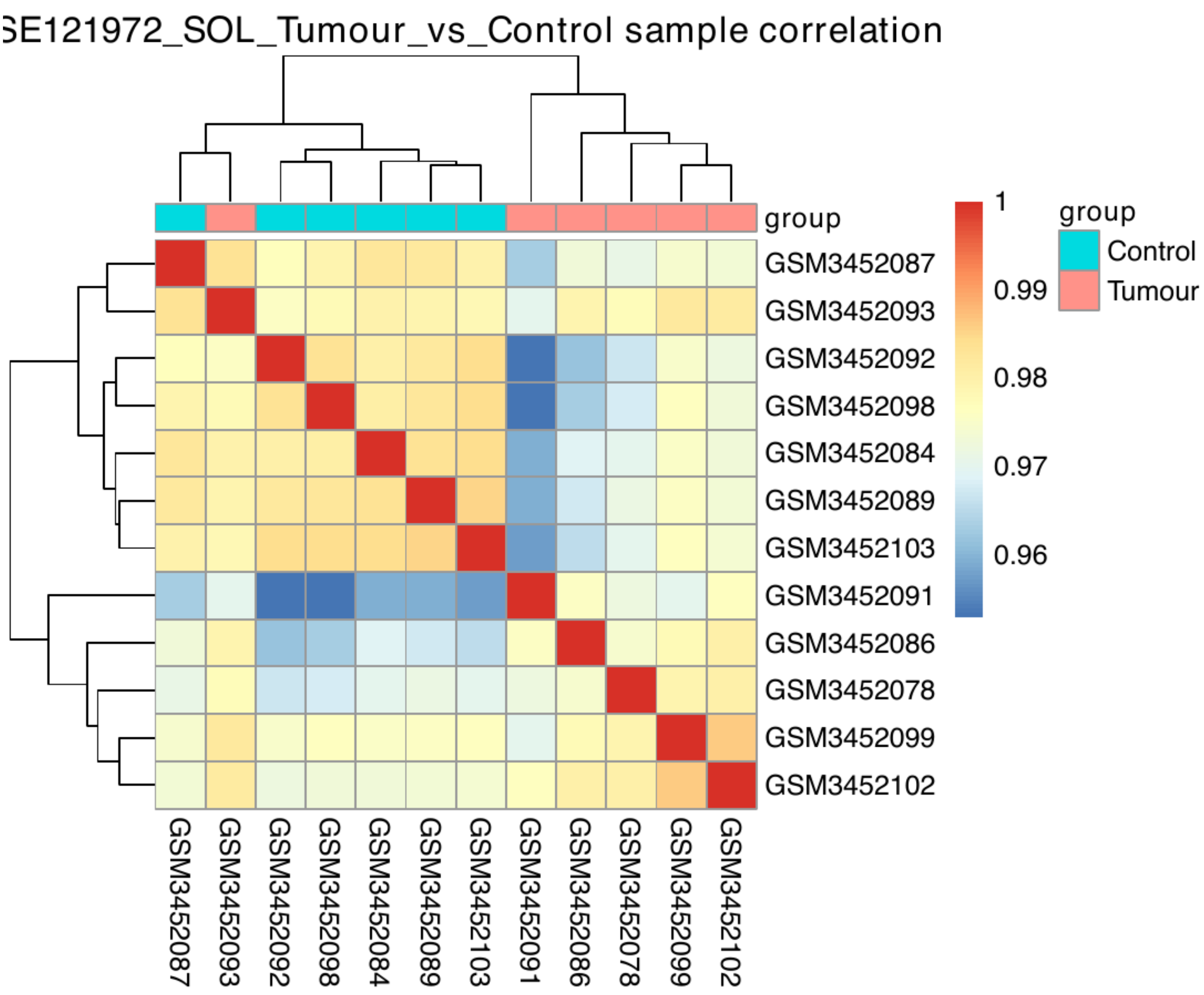


**Figure S23.** GSE121972 SOL Tumour bearing versus Control: sample correlation heatmap.

Original figure file: GSE121972_SOL_Tumour_vs_Control_QC_sample_correlation.pdf

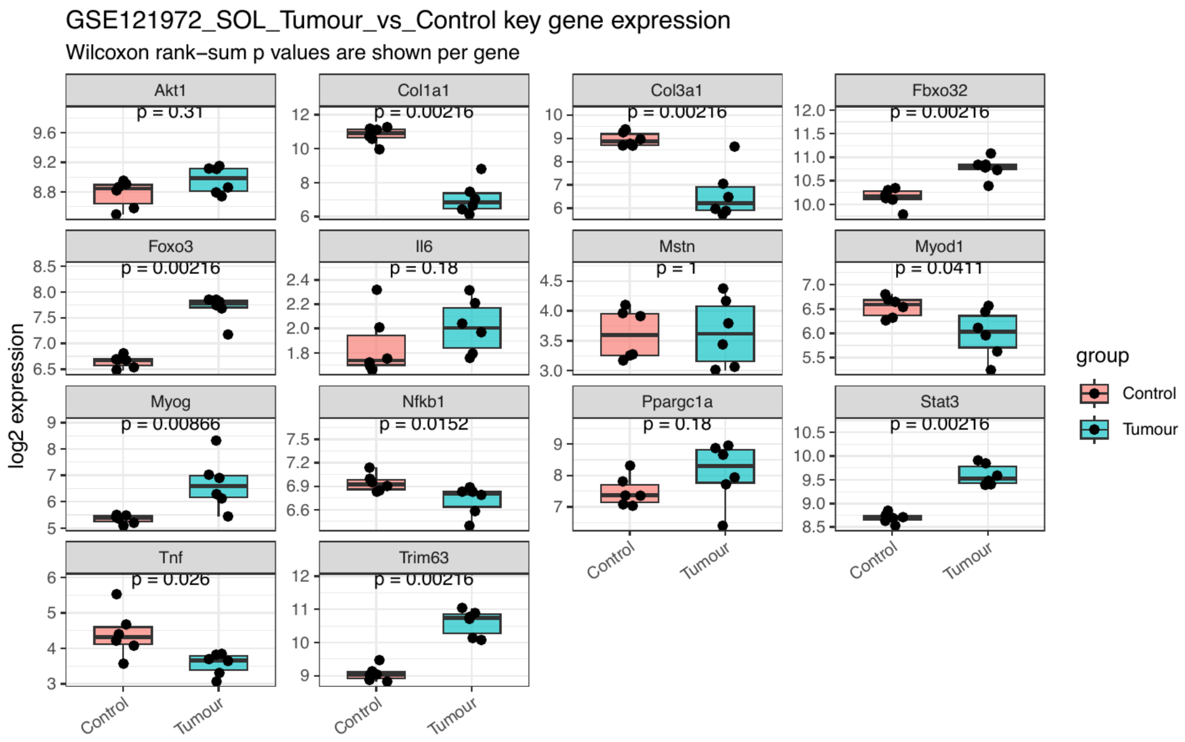


**Figure S24.** GSE121972 SOL Tumour bearing versus Control: key gene expression plot.

Original figure file: GSE121972_SOL_Tumour_vs_Control_key_gene_expression.pdf

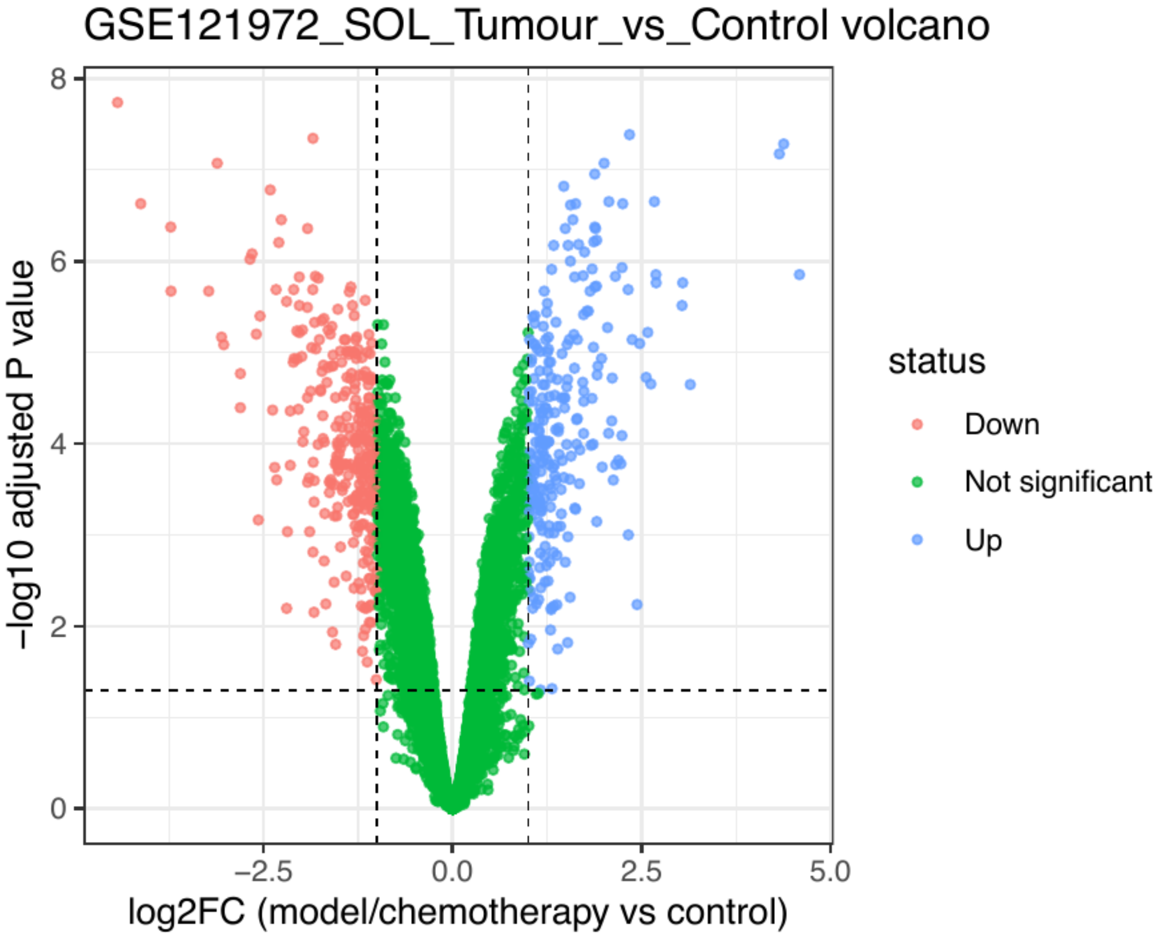


**Figure S25.** GSE121972 SOL Tumour bearing versus Control: differential expression volcano plot.

Original figure file: GSE121972_SOL_Tumour_vs_Control_volcano.pdf

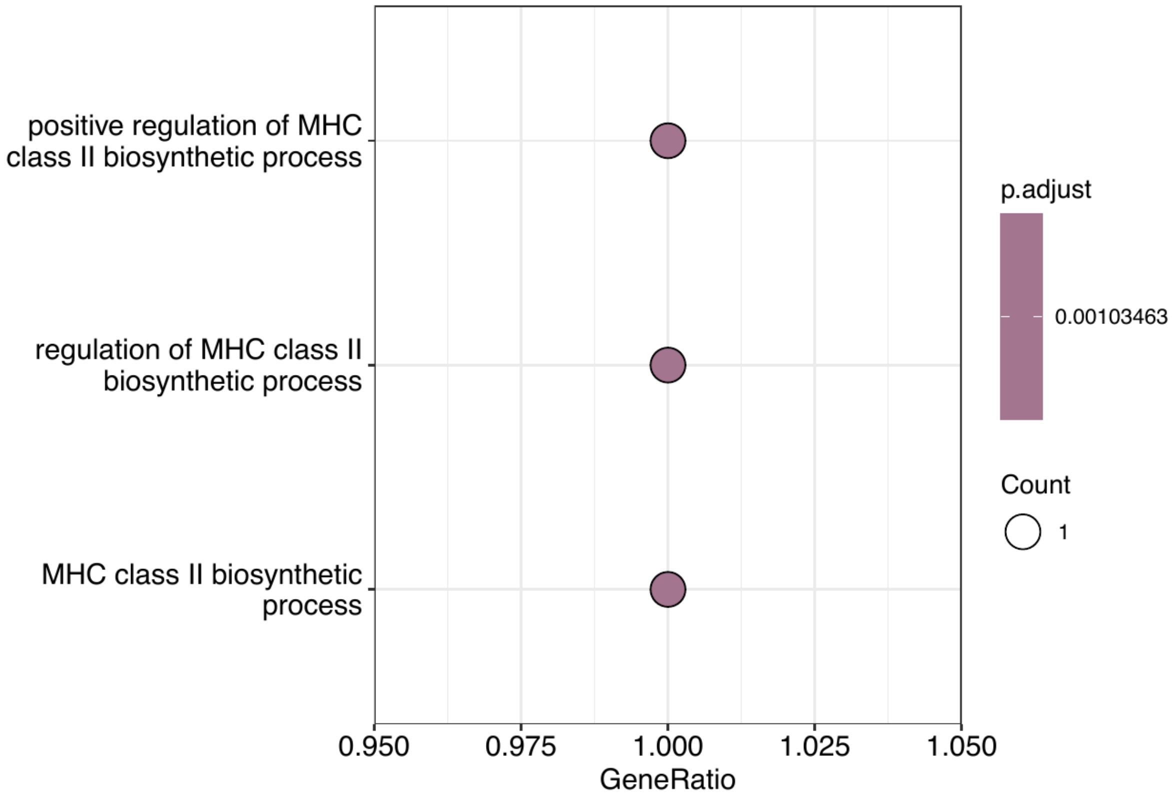


**Figure S26.** GSE77745 Doxorubicin versus Control: GO Biological Process enrichment dot plot.

Original figure file: GSE77745_Doxorubicin_vs_Control_GO_BP_dotplot.pdf

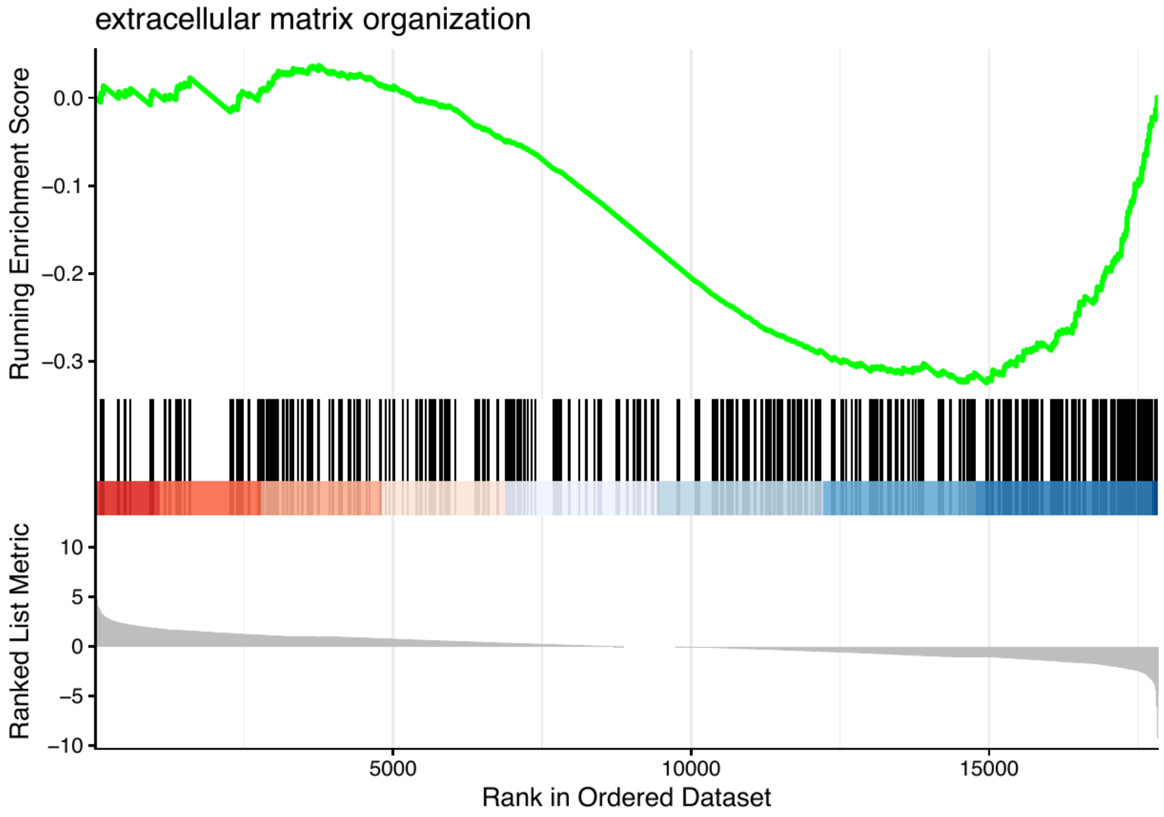


**Figure S27.** GSE77745 Doxorubicin versus Control: GSEA curves for extracellular matrix-related GO BP terms.

Original figure file: GSE77745_Doxorubicin_vs_Control_GSEA_GO_BP_ecm.pdf

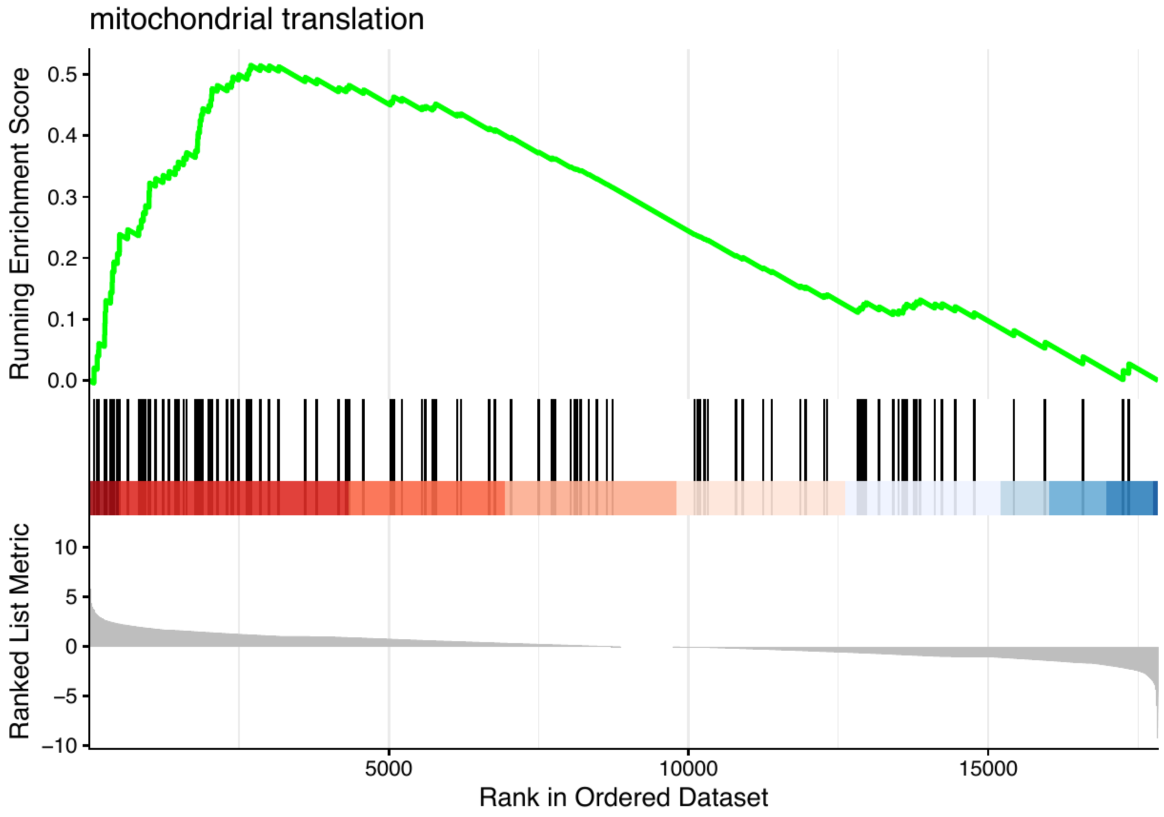


**Figure S28.** GSE77745 Doxorubicin versus Control: GSEA curves for energy metabolism-related GO BP terms.

Original figure file: GSE77745_Doxorubicin_vs_Control_GSEA_GO_BP_energy.pdf

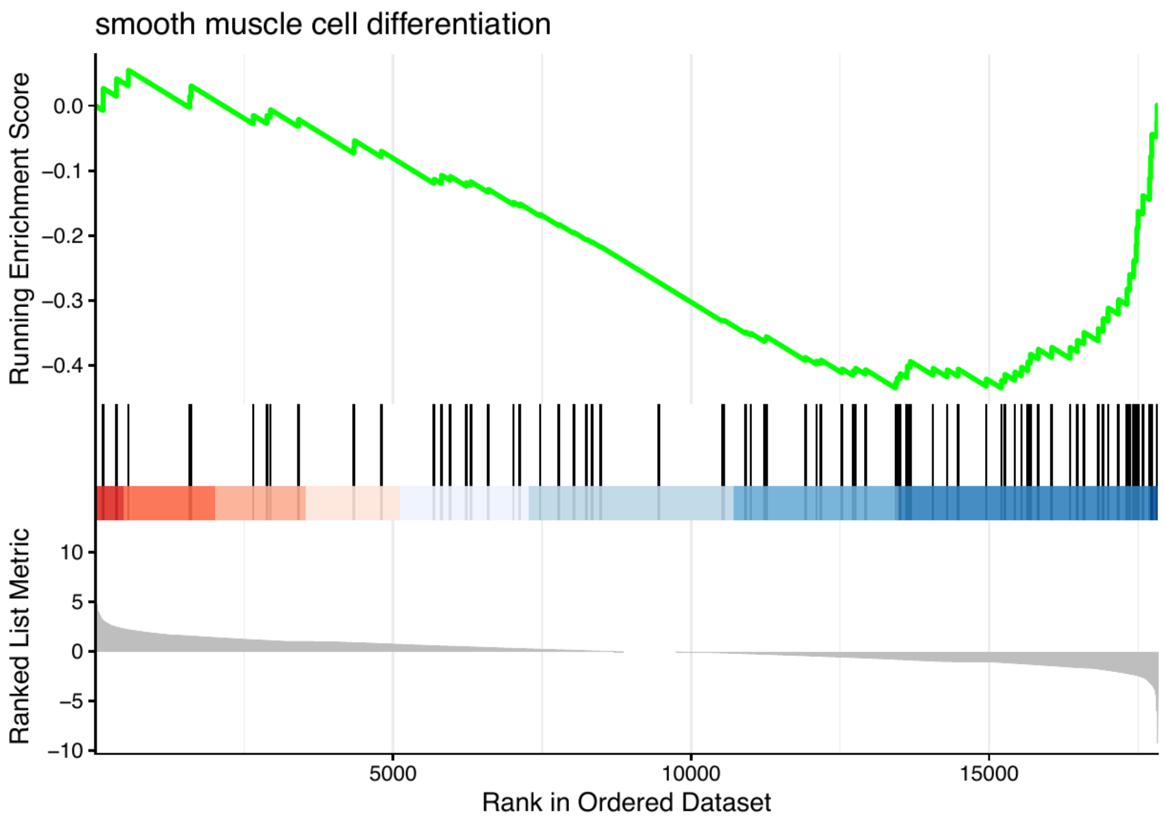


**Figure S29.** GSE77745 Doxorubicin versus Control: GSEA curves for muscle function-related GO BP terms.

Original figure file: GSE77745_Doxorubicin_vs_Control_GSEA_GO_BP_muscle.pdf

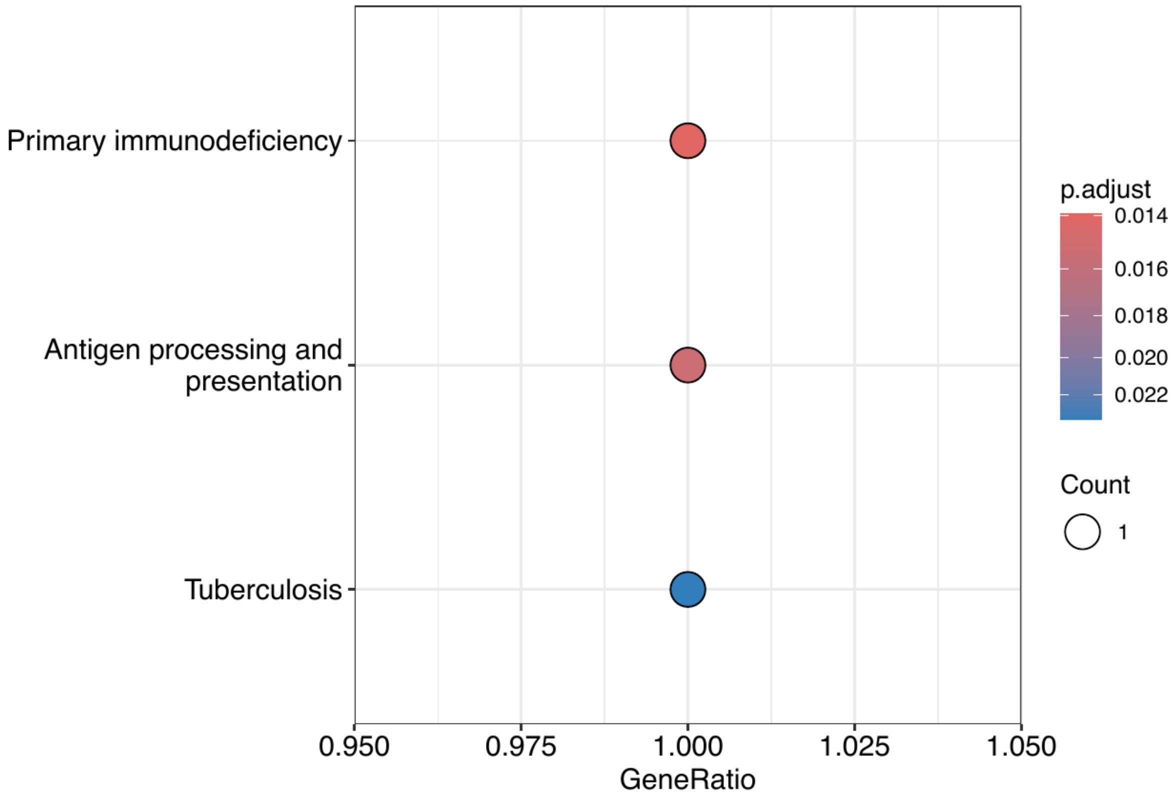


**Figure S30.** GSE77745 Doxorubicin versus Control: KEGG enrichment dot plot.

Original figure file: GSE77745_Doxorubicin_vs_Control_KEGG_dotplot.pdf

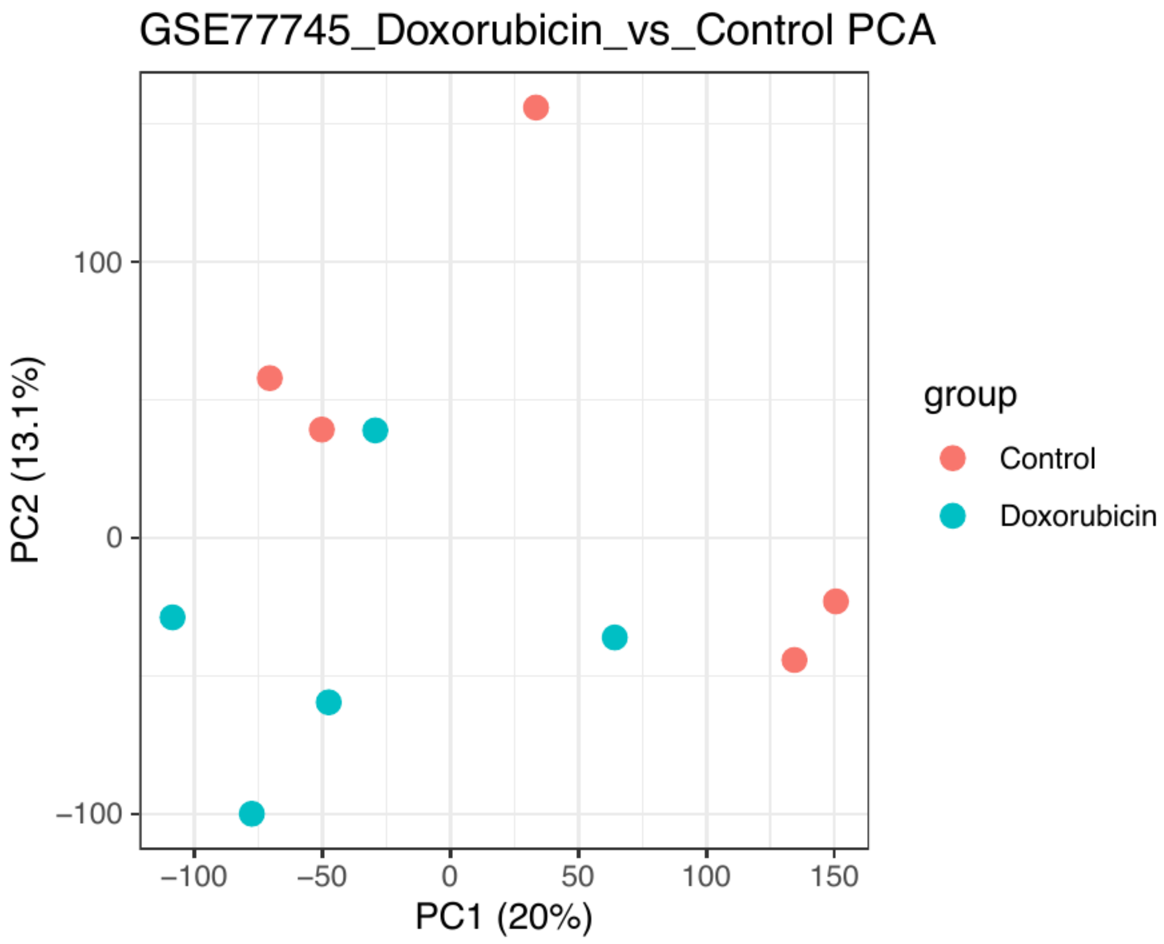


**Figure S31.** GSE77745 Doxorubicin versus Control: principal component analysis plot.

Original figure file: GSE77745_Doxorubicin_vs_Control_QC_PCA.pdf

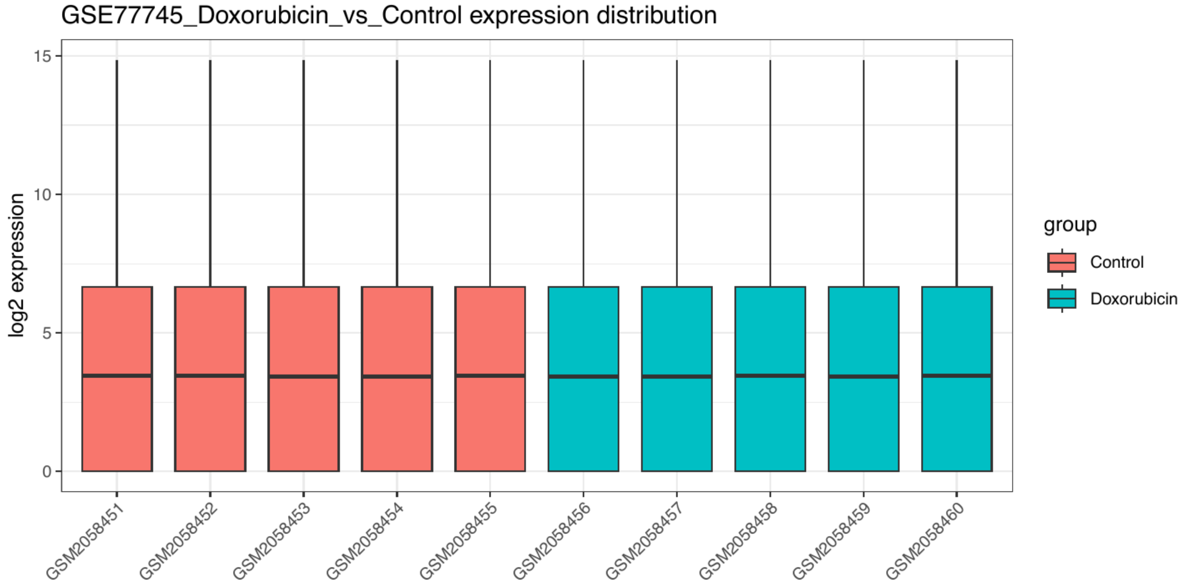


**Figure S32.** GSE77745 Doxorubicin versus Control: expression distribution boxplot.

Original figure file: GSE77745_Doxorubicin_vs_Control_QC_boxplot.pdf

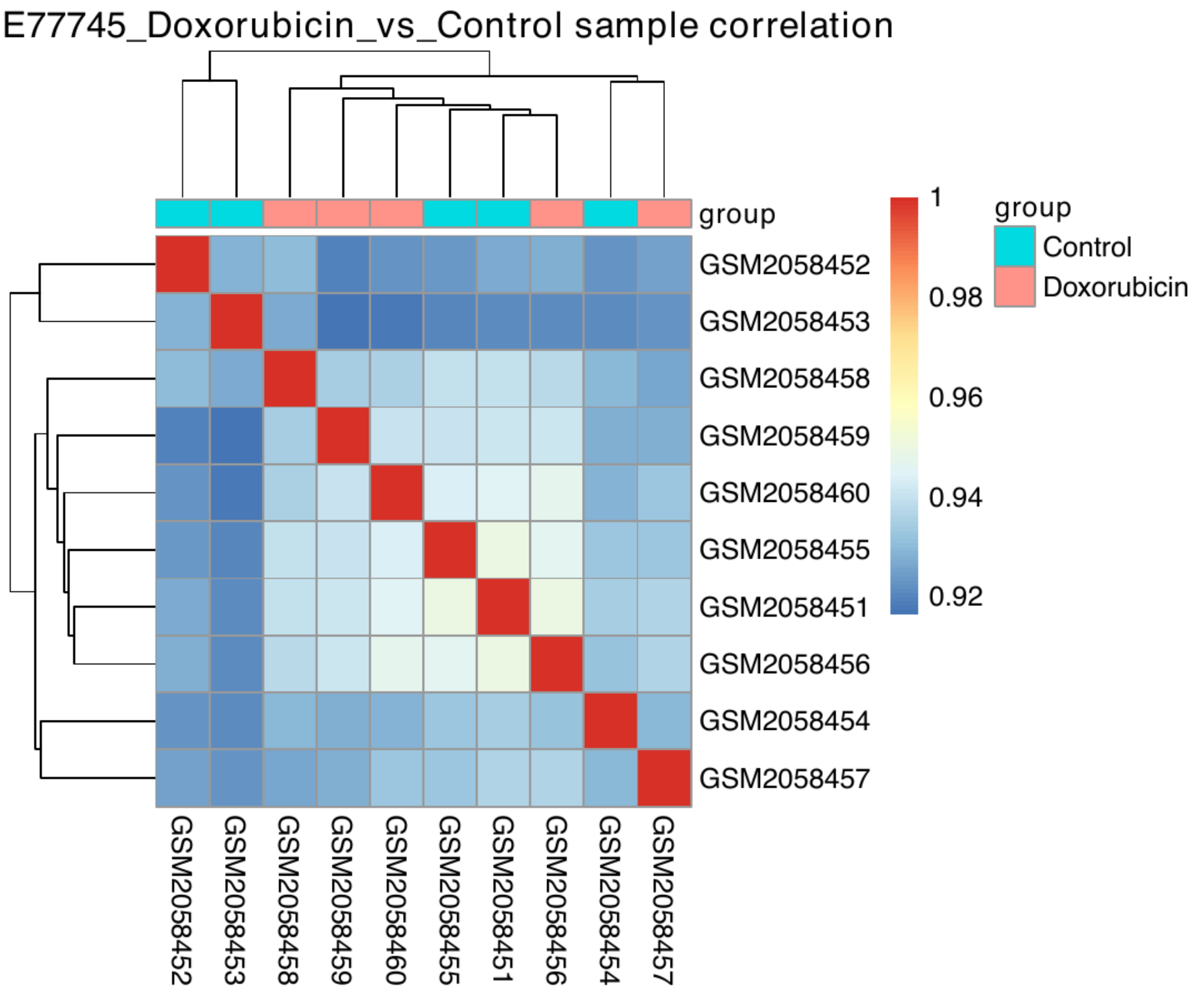


**Figure S33.** GSE77745 Doxorubicin versus Control: sample correlation heatmap.

Original figure file: GSE77745_Doxorubicin_vs_Control_QC_sample_correlation.pdf

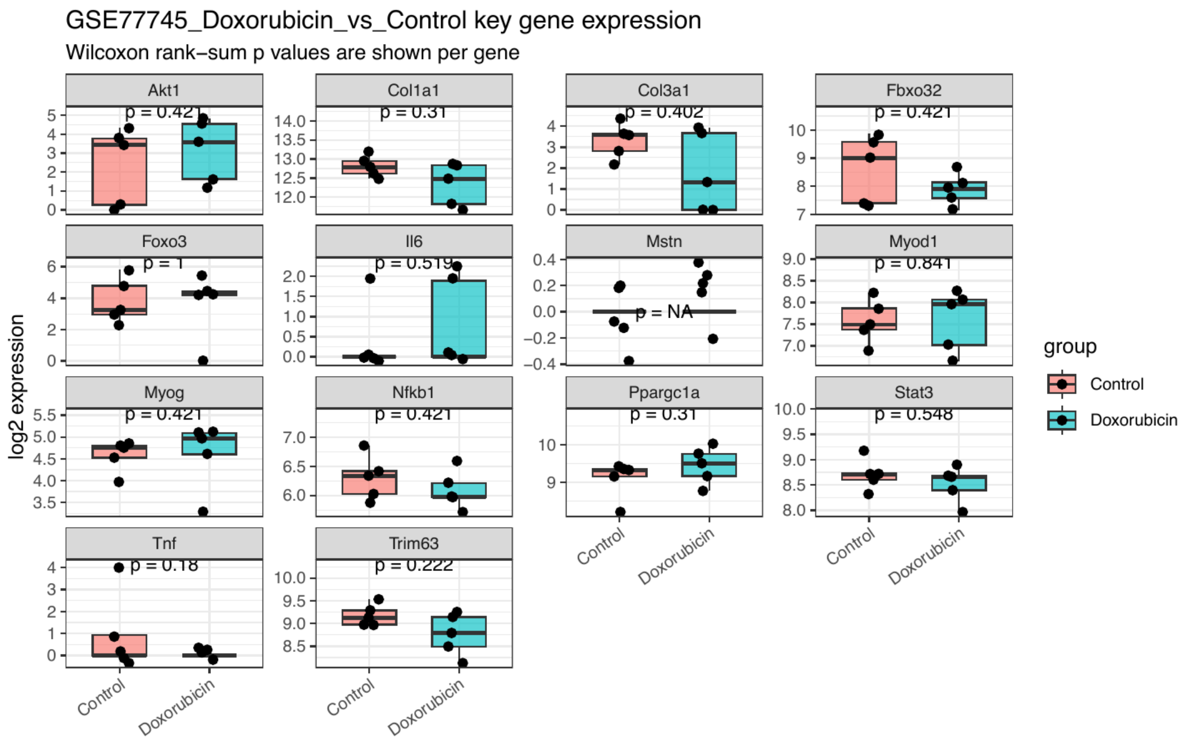


**Figure S34.** GSE77745 Doxorubicin versus Control: key gene expression plot.

Original figure file: GSE77745_Doxorubicin_vs_Control_key_gene_expression.pdf

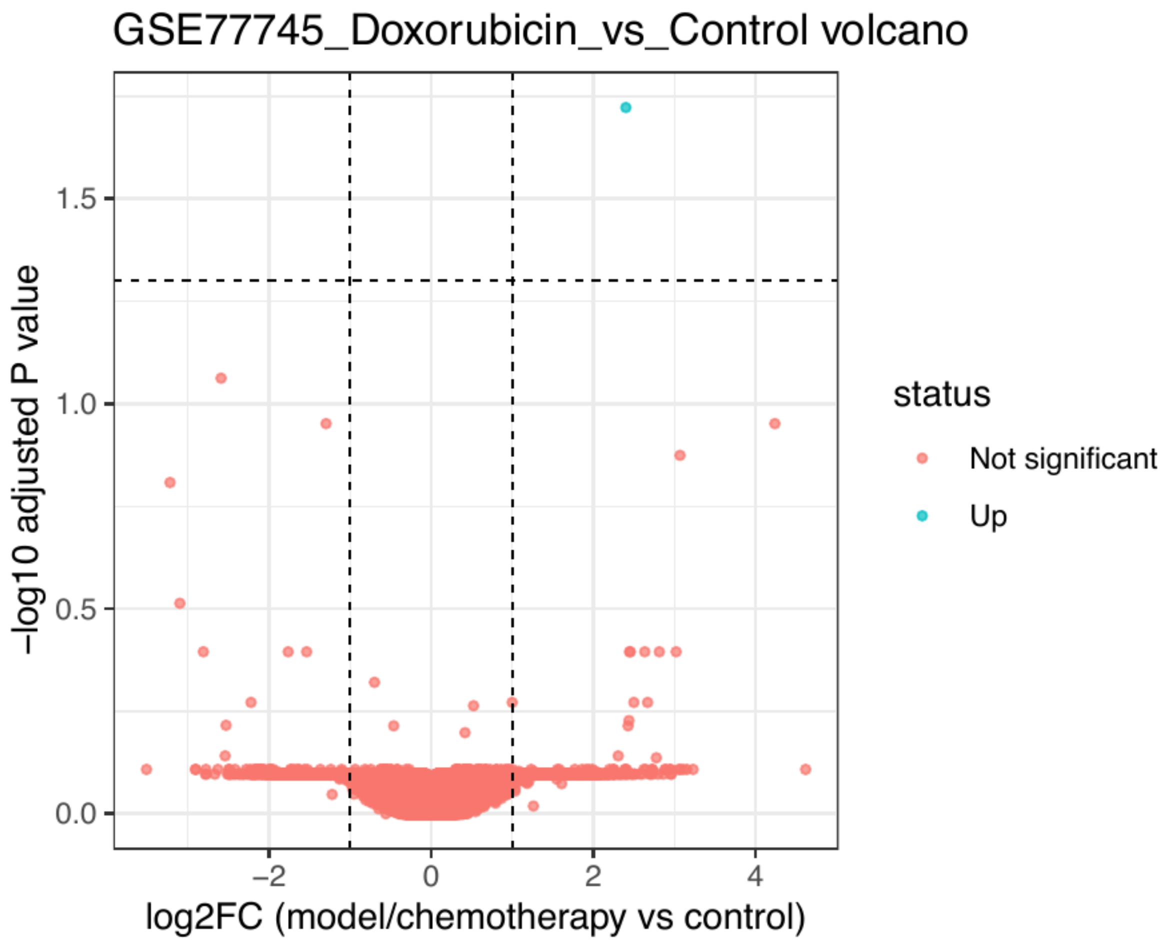


**Figure S35.** GSE77745 Doxorubicin versus Control: differential expression volcano plot.

Original figure file: GSE77745_Doxorubicin_vs_Control_volcano.pdf

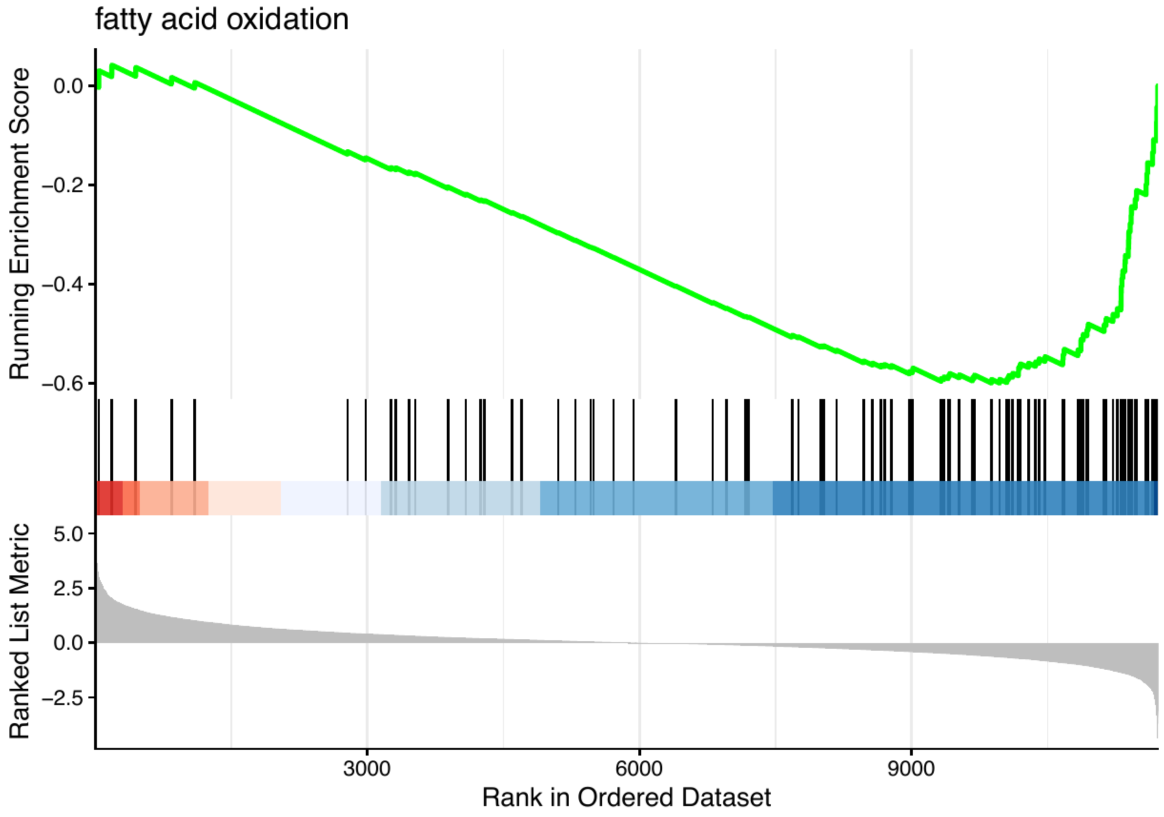


**Figure S36.** GSE80473 FOLFIRI versus Vehicle: GSEA curves for energy metabolism-related GO BP terms.

Original figure file: GSE80473_FOLFIRI_vs_Vehicle_GSEA_GO_BP_energy.pdf

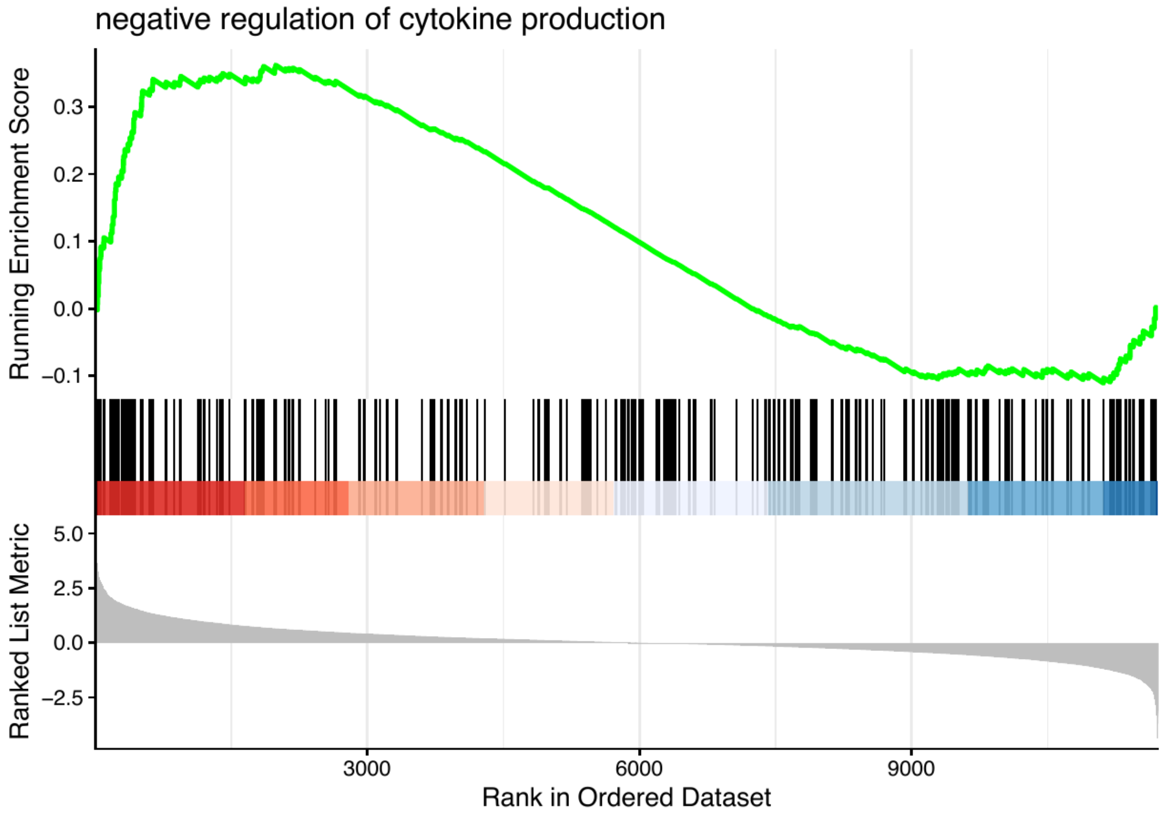


**Figure S37.** GSE80473 FOLFIRI versus Vehicle: GSEA curves for inflammatory response-related GO BP terms.

Original figure file: GSE80473_FOLFIRI_vs_Vehicle_GSEA_GO_BP_inflammatory.pdf

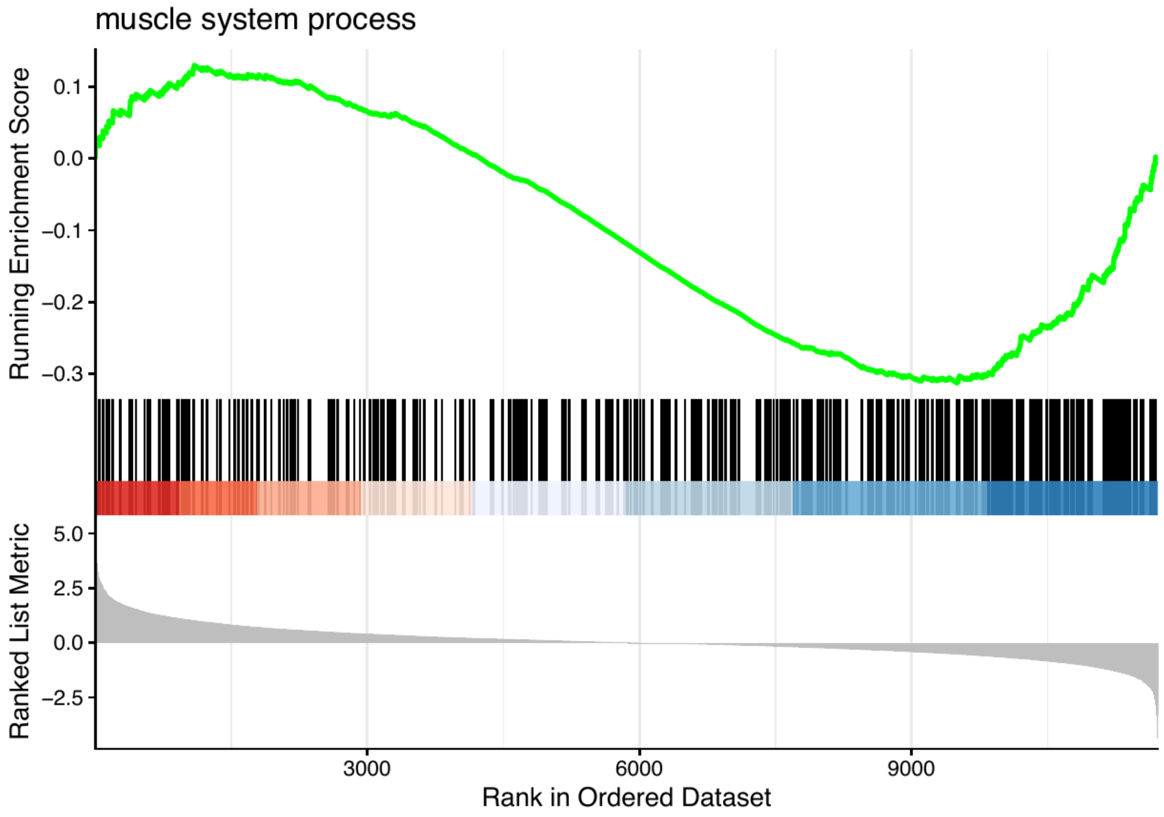


**Figure S38.** GSE80473 FOLFIRI versus Vehicle: GSEA curves for muscle function-related GO BP terms.

Original figure file: GSE80473_FOLFIRI_vs_Vehicle_GSEA_GO_BP_muscle.pdf

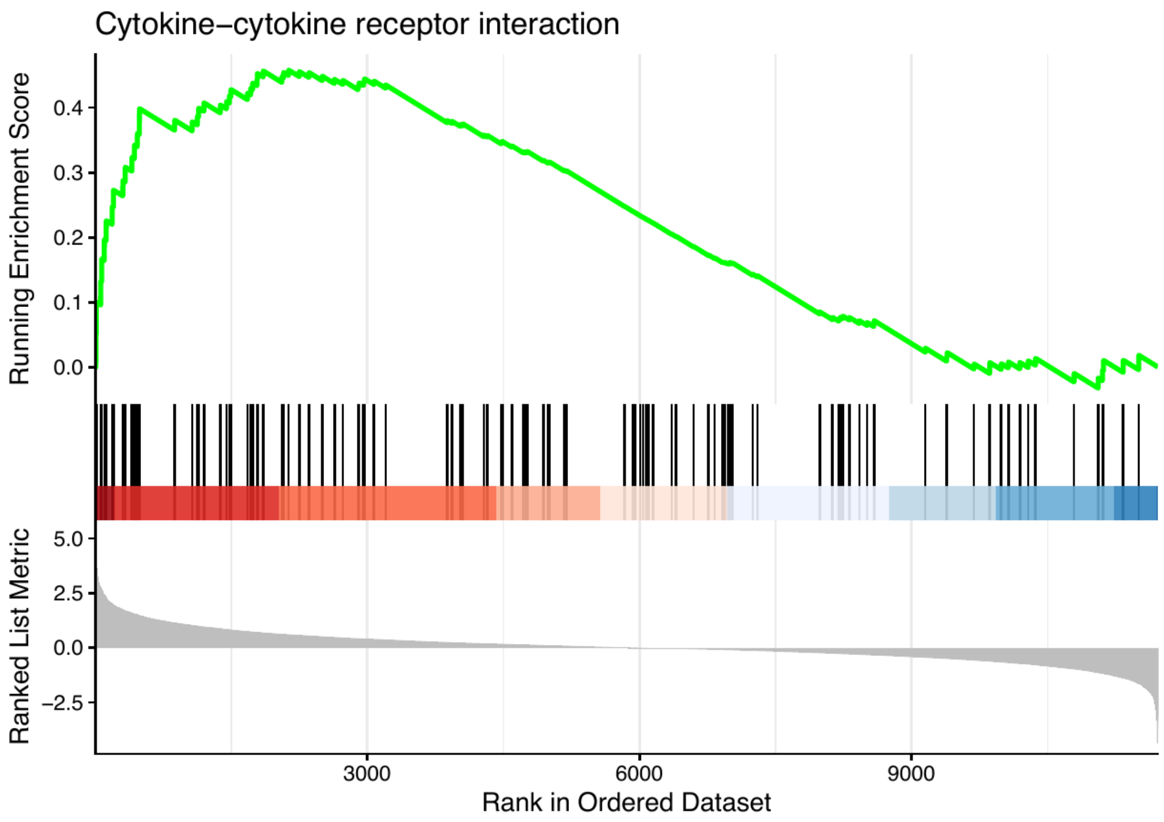


**Figure S39.** GSE80473 FOLFIRI versus Vehicle: GSEA curves for KEGG cytokine-related pathways.

Original figure file: GSE80473_FOLFIRI_vs_Vehicle_GSEA_KEGG_cytokine.pdf

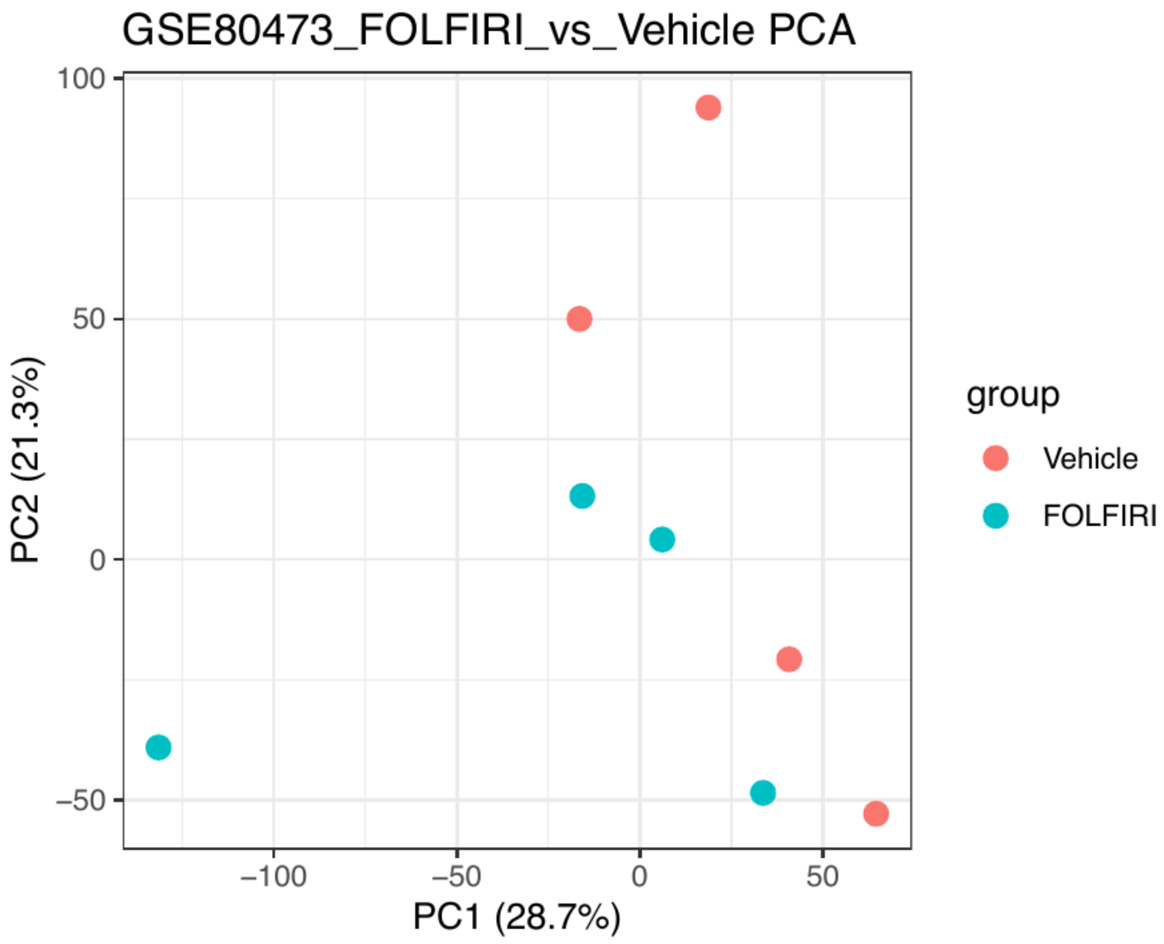


**Figure S40.** GSE80473 FOLFIRI versus Vehicle: principal component analysis plot.

Original figure file: GSE80473_FOLFIRI_vs_Vehicle_QC_PCA.pdf

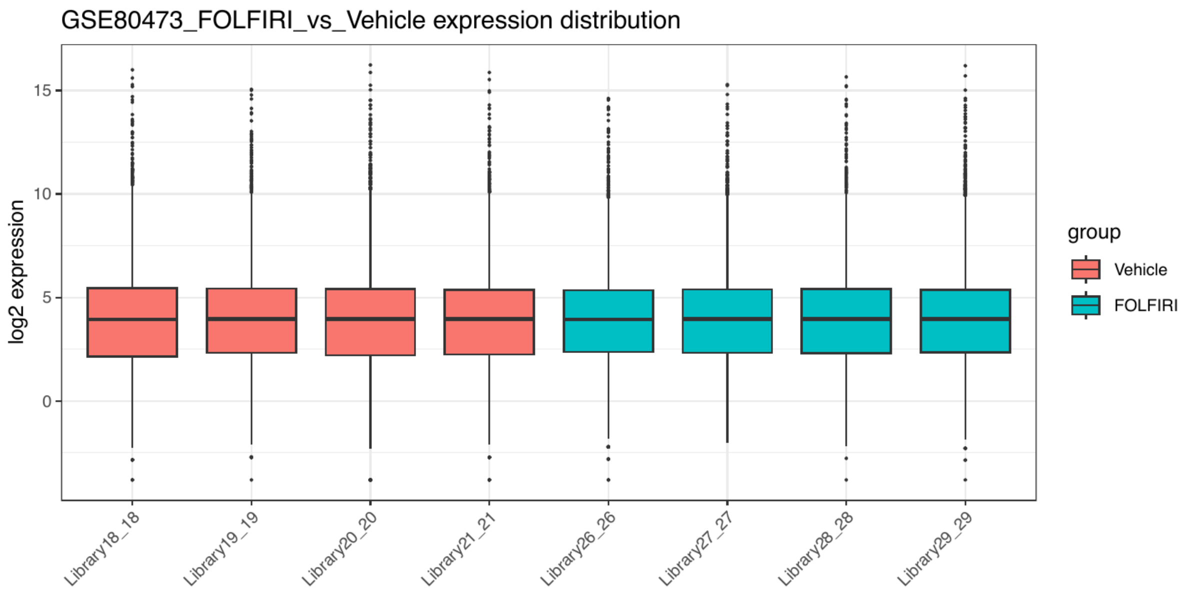


**Figure S41.** GSE80473 FOLFIRI versus Vehicle: expression distribution boxplot.

Original figure file: GSE80473_FOLFIRI_vs_Vehicle_QC_boxplot.pdf

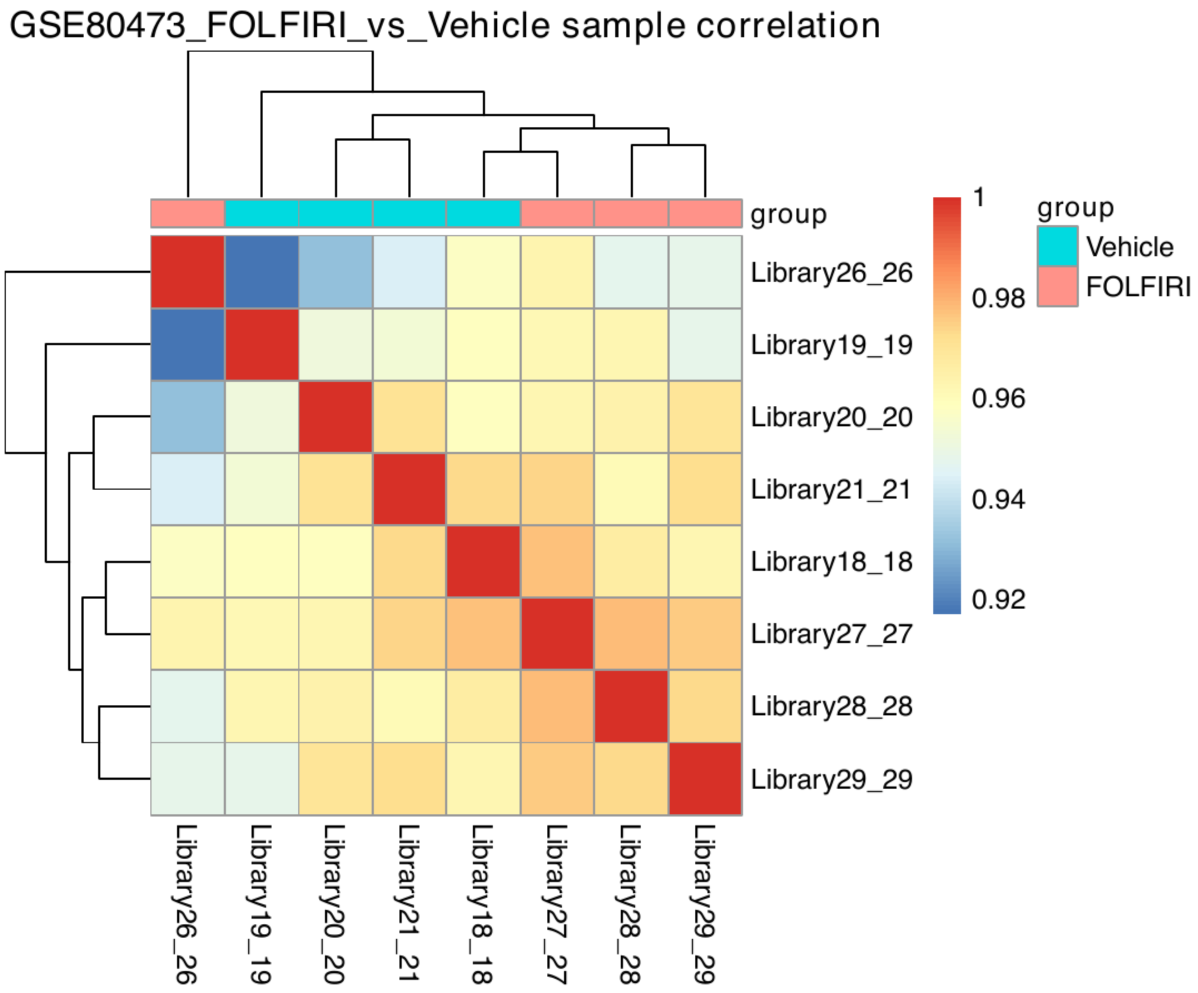


**Figure S42.** GSE80473 FOLFIRI versus Vehicle: sample correlation heatmap.

Original figure file: GSE80473_FOLFIRI_vs_Vehicle_QC_sample_correlation.pdf

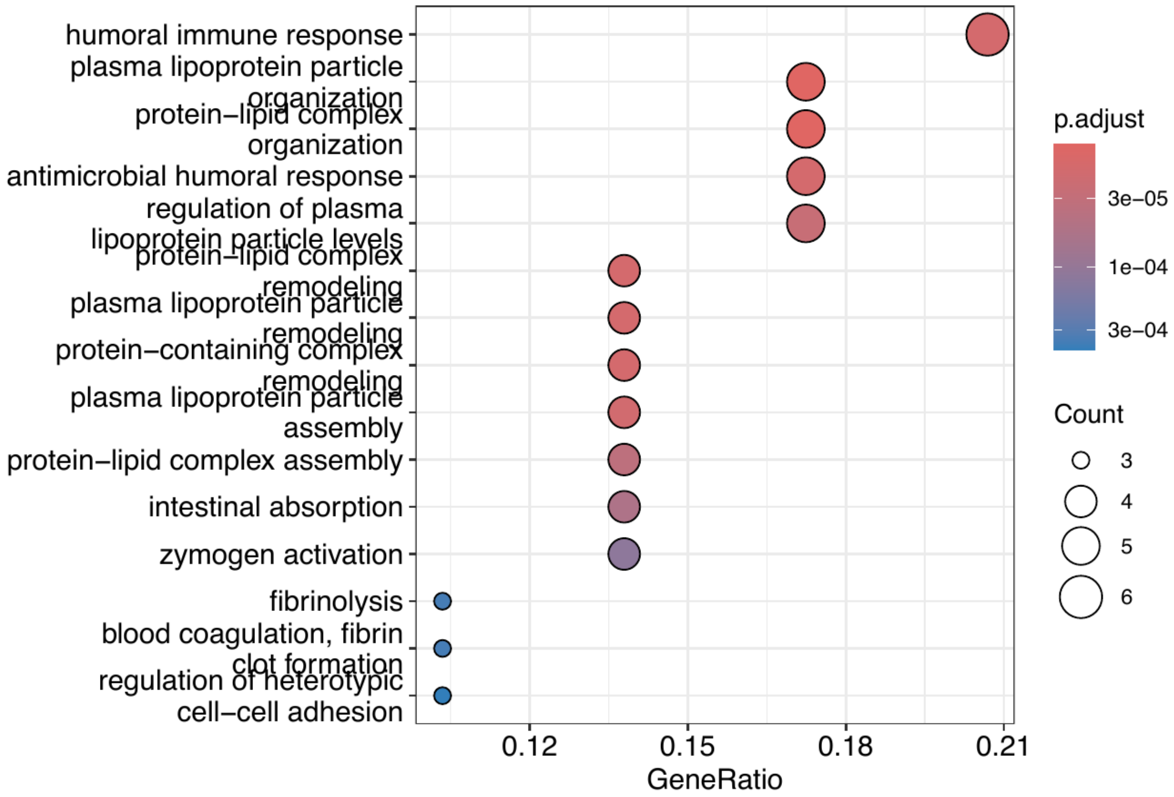


**Figure S43.** GSE80473 depositor-provided edgeR sensitivity analysis: GO Biological Process enrichment dot plot.

Original figure file: GSE80473_FOLFIRI_vs_Vehicle_depositor_edgeR_GO_BP_dotplot.pdf

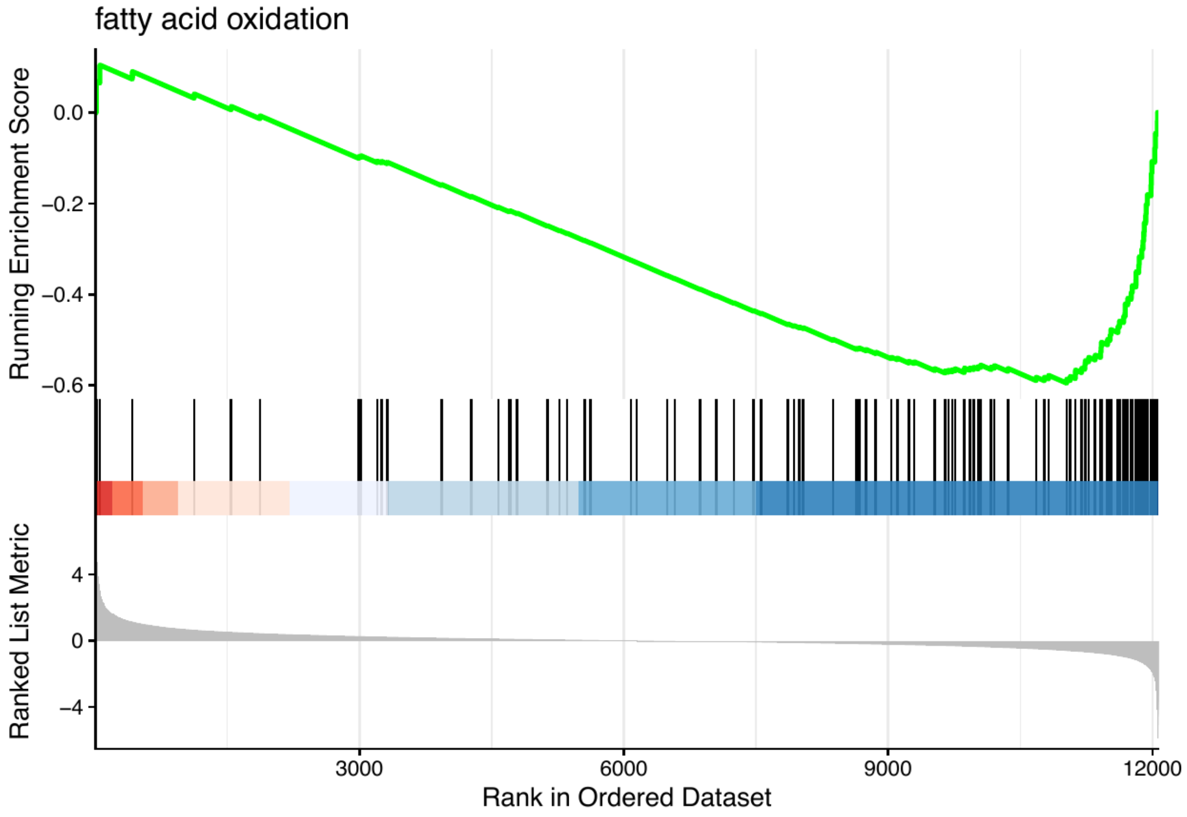


**Figure S44.** GSE80473 depositor-provided edgeR sensitivity analysis: GSEA curves for energy metabolism-related GO BP terms.

Original figure file: GSE80473_FOLFIRI_vs_Vehicle_depositor_edgeR_GSEA_GO_BP_energy.pdf

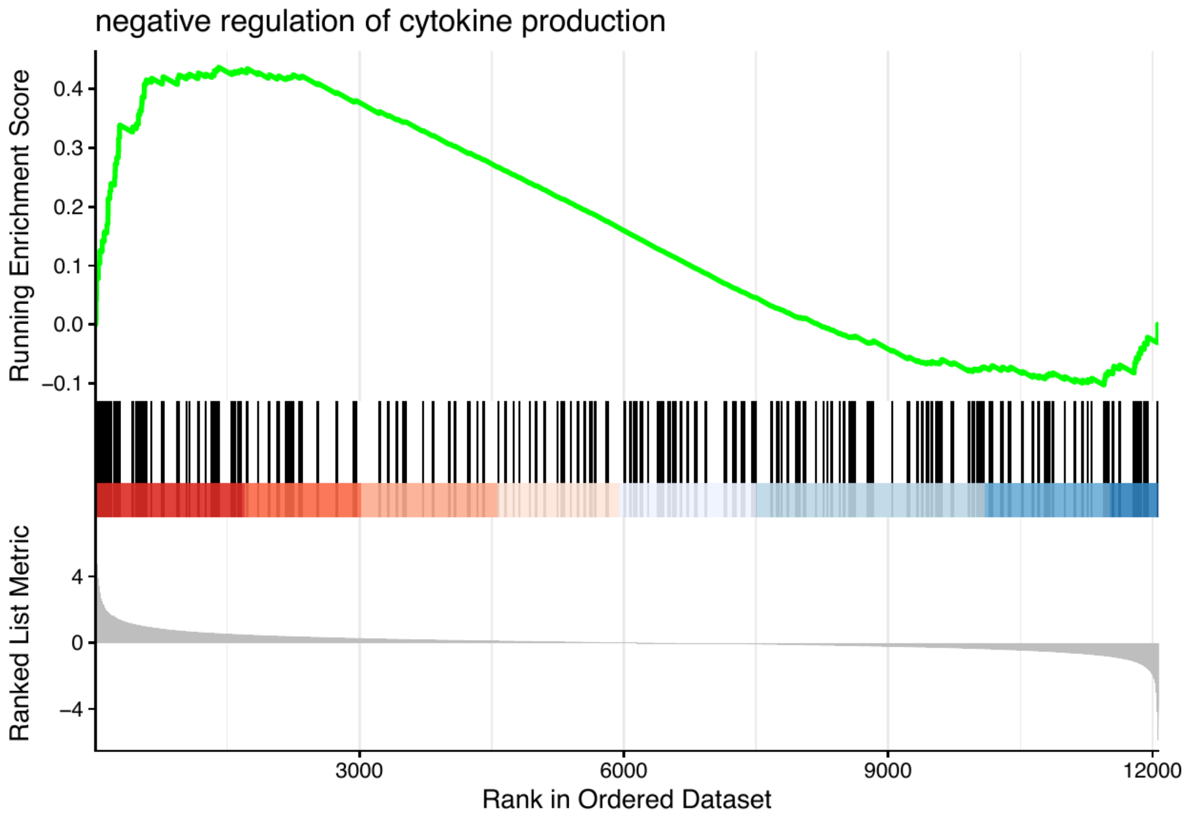


**Figure S45.** GSE80473 depositor-provided edgeR sensitivity analysis: GSEA curves for inflammatory response-related GO BP terms.

Original figure file: GSE80473_FOLFIRI_vs_Vehicle_depositor_edgeR_GSEA_GO_BP_inflammatory.pdf

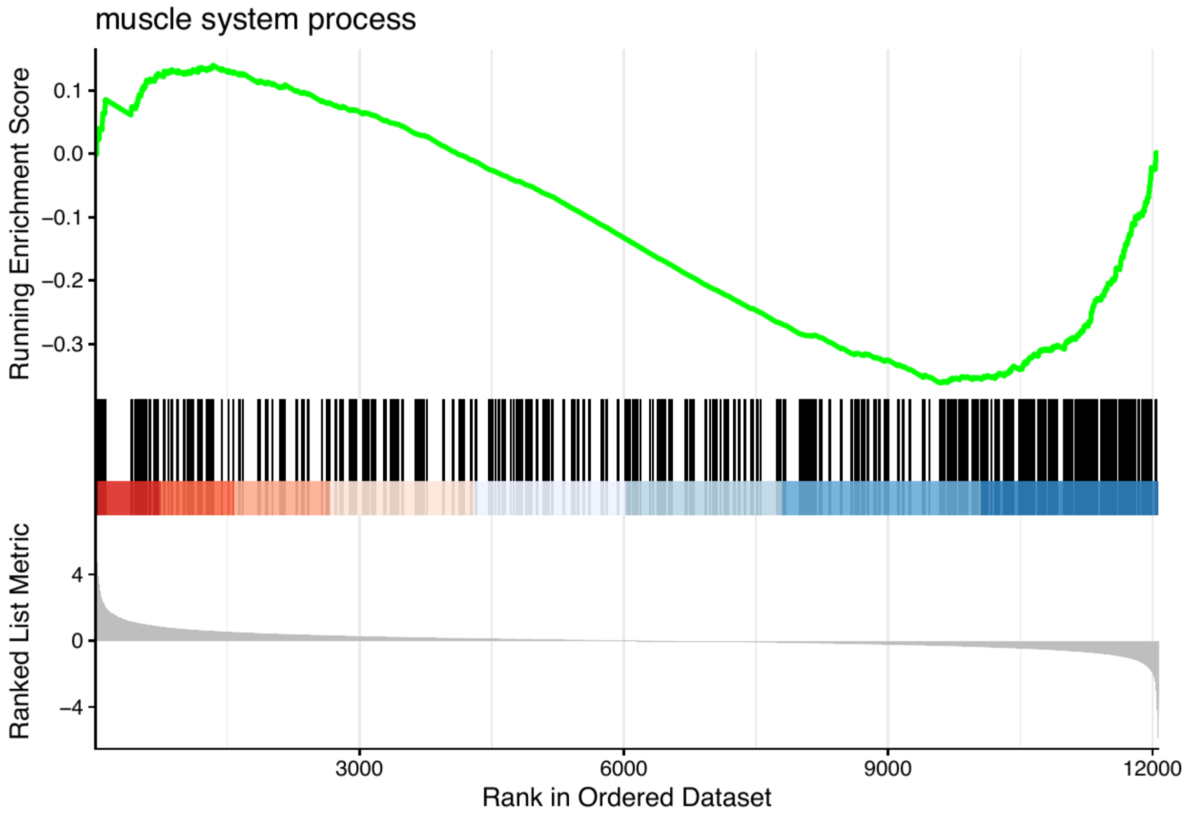


**Figure S46.** GSE80473 depositor-provided edgeR sensitivity analysis: GSEA curves for muscle function-related GO BP terms.

Original figure file: GSE80473_FOLFIRI_vs_Vehicle_depositor_edgeR_GSEA_GO_BP_muscle.pdf

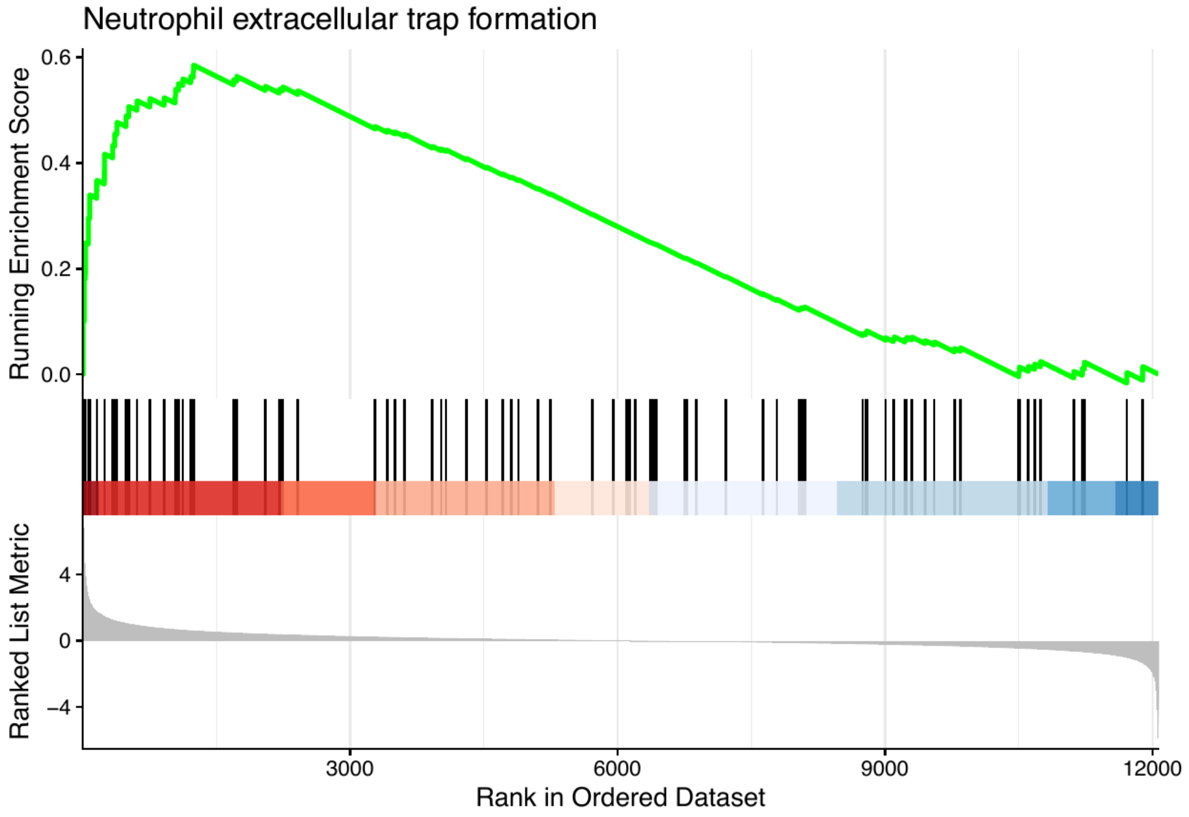


**Figure S47.** GSE80473 depositor-provided edgeR sensitivity analysis: GSEA curves for KEGG cytokine-related pathways.

Original figure file: GSE80473_FOLFIRI_vs_Vehicle_depositor_edgeR_GSEA_KEGG_cytokine.pdf

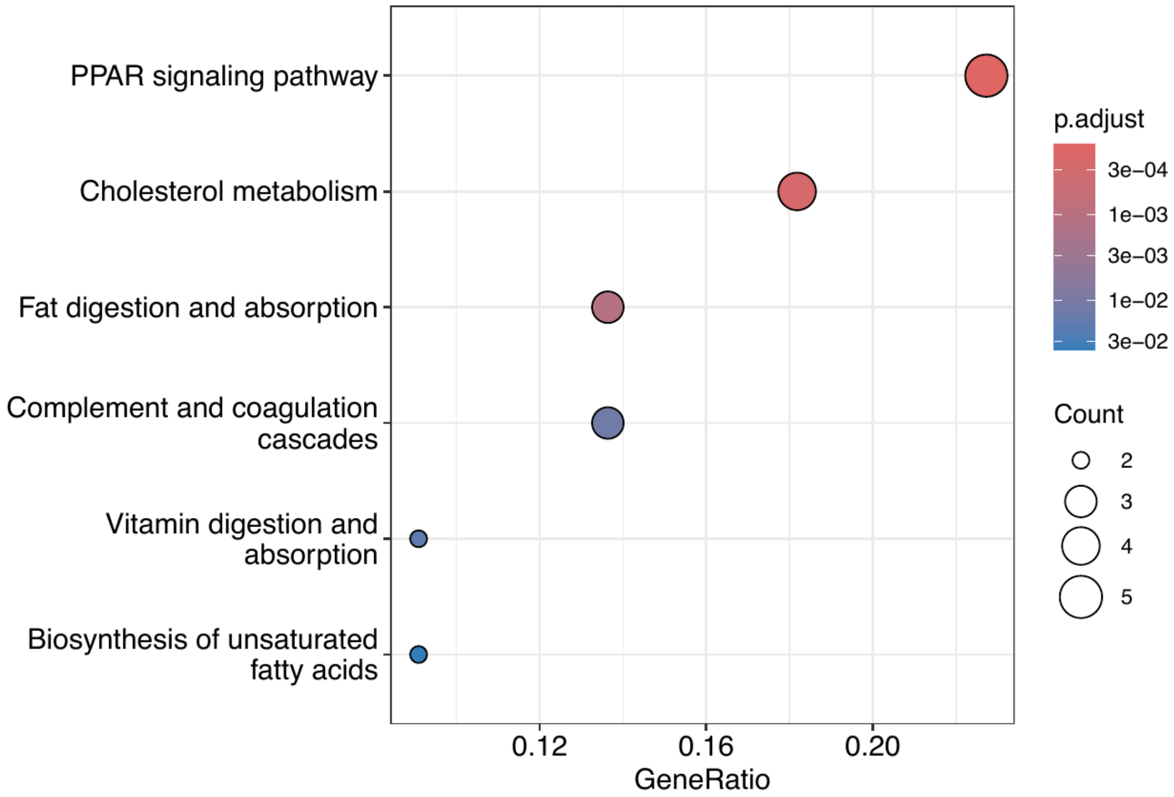


**Figure S48.** GSE80473 depositor-provided edgeR sensitivity analysis: KEGG enrichment dot plot.

Original figure file: GSE80473_FOLFIRI_vs_Vehicle_depositor_edgeR_KEGG_dotplot.pdf

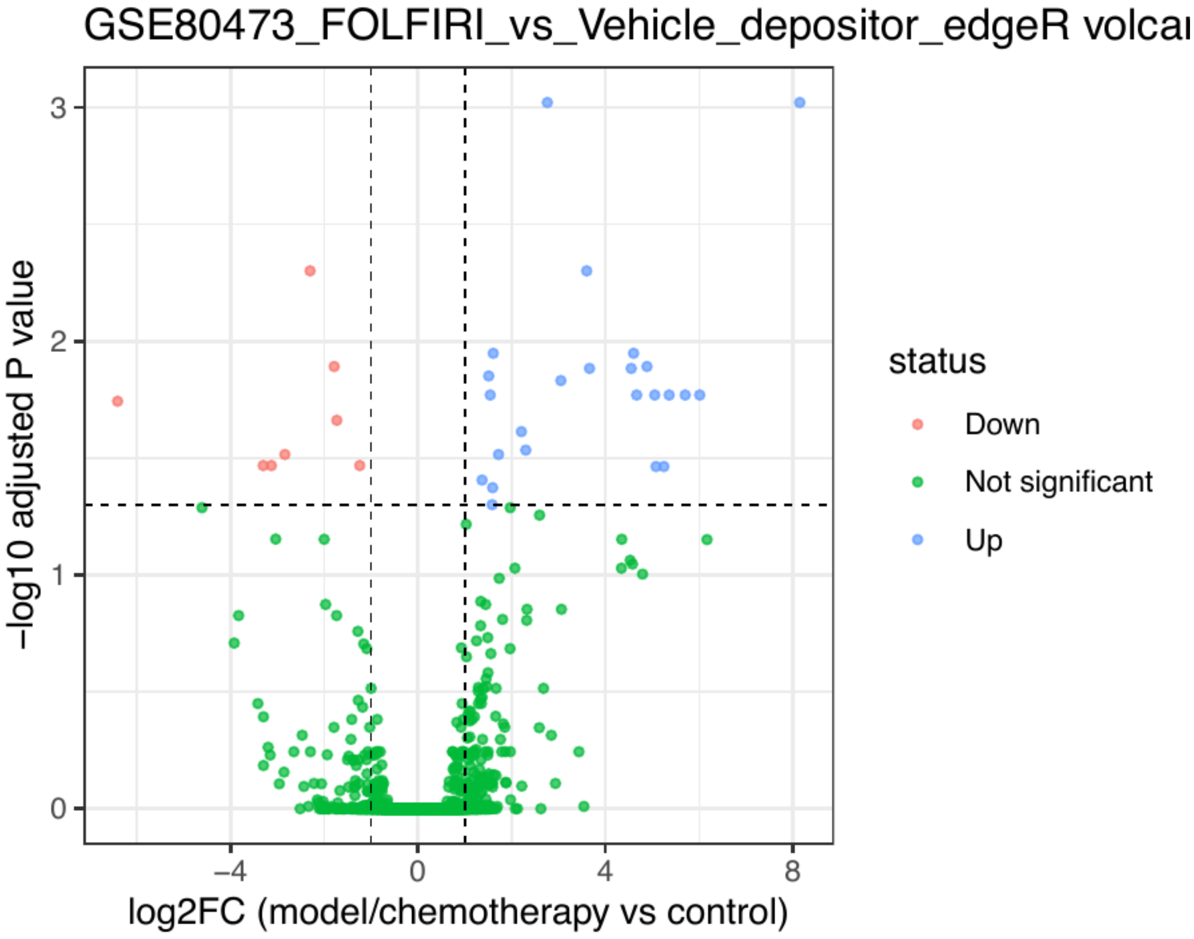


**Figure S49.** GSE80473 depositor-provided edgeR sensitivity analysis: differential expression volcano plot.

Original figure file: GSE80473_FOLFIRI_vs_Vehicle_depositor_edgeR_volcano.pdf

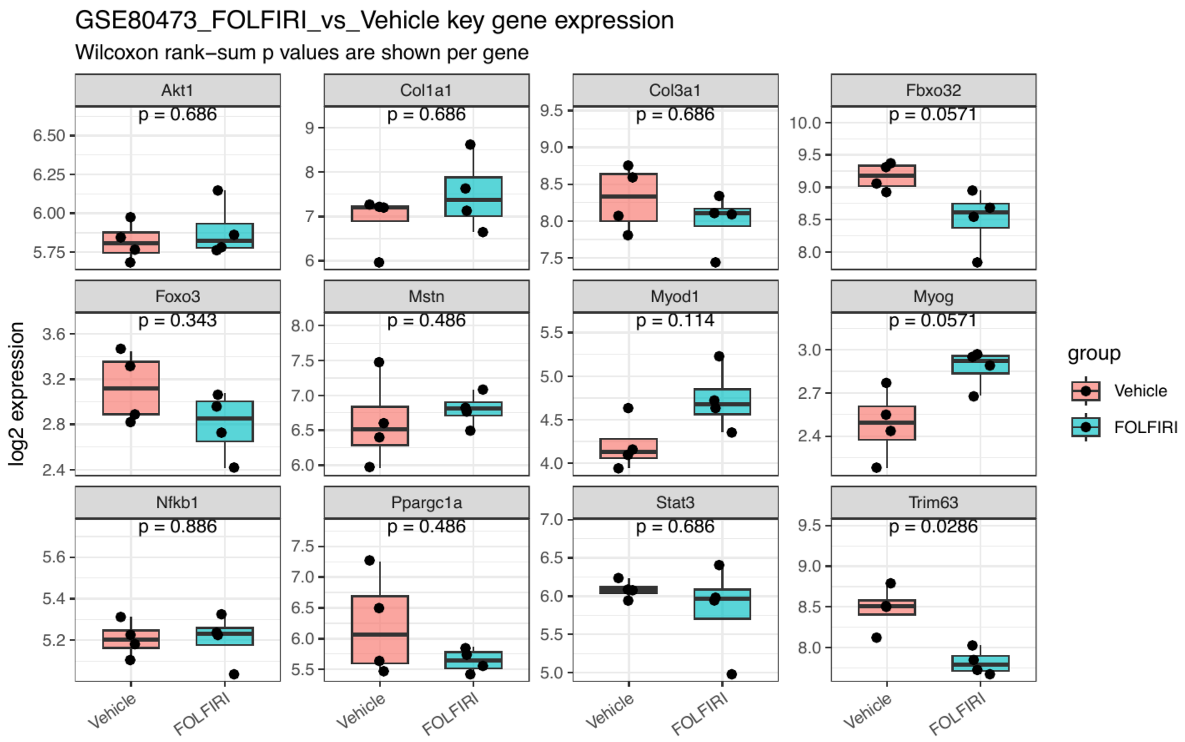


**Figure S50.** GSE80473 FOLFIRI versus Vehicle: key gene expression plot.

Original figure file: GSE80473_FOLFIRI_vs_Vehicle_key_gene_expression.pdf

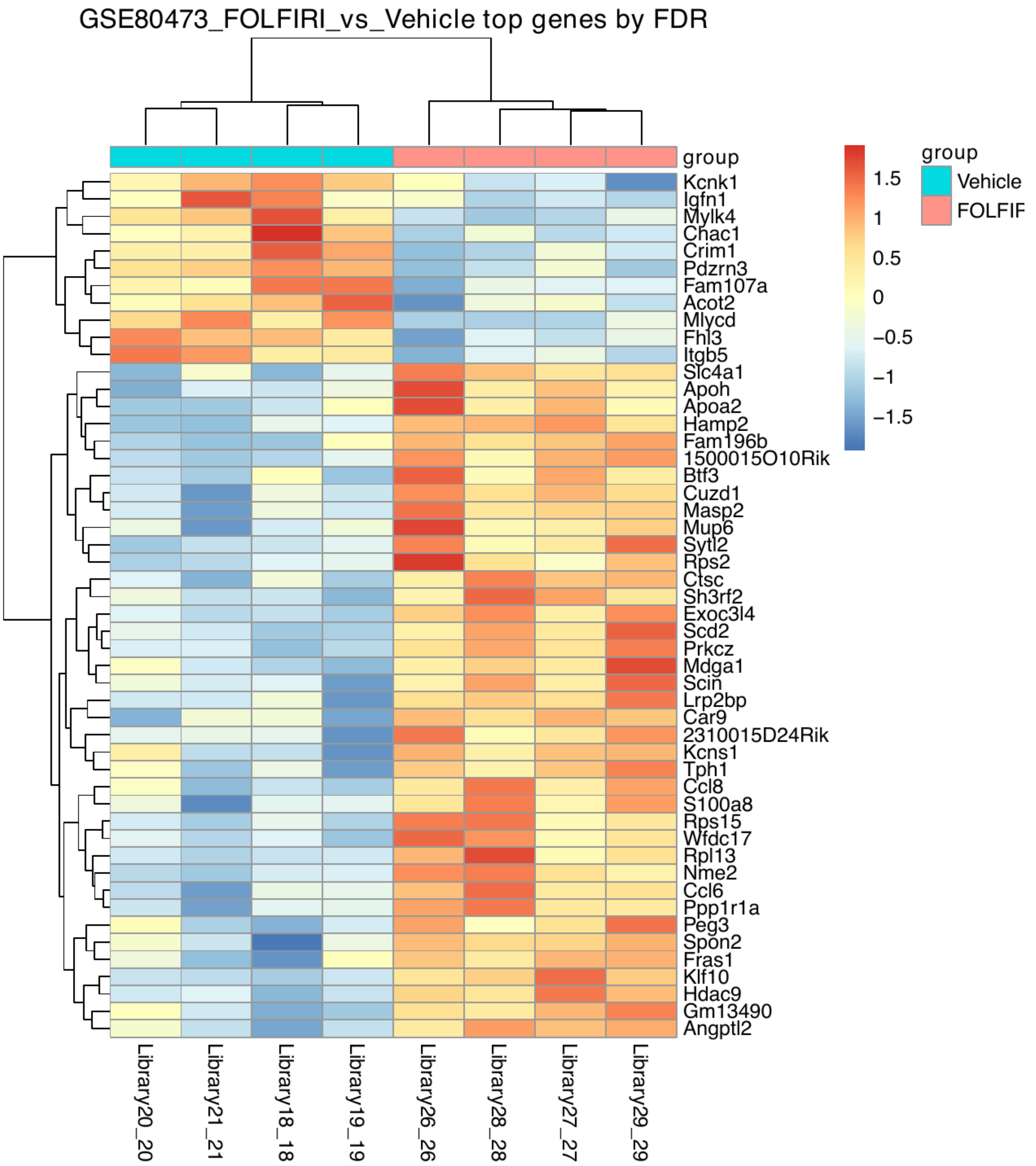


**Figure S51.** GSE80473 FOLFIRI versus Vehicle: heatmap of the top 50 variable or differentially associated genes.

Original figure file: GSE80473_FOLFIRI_vs_Vehicle_top50_heatmap.pdf

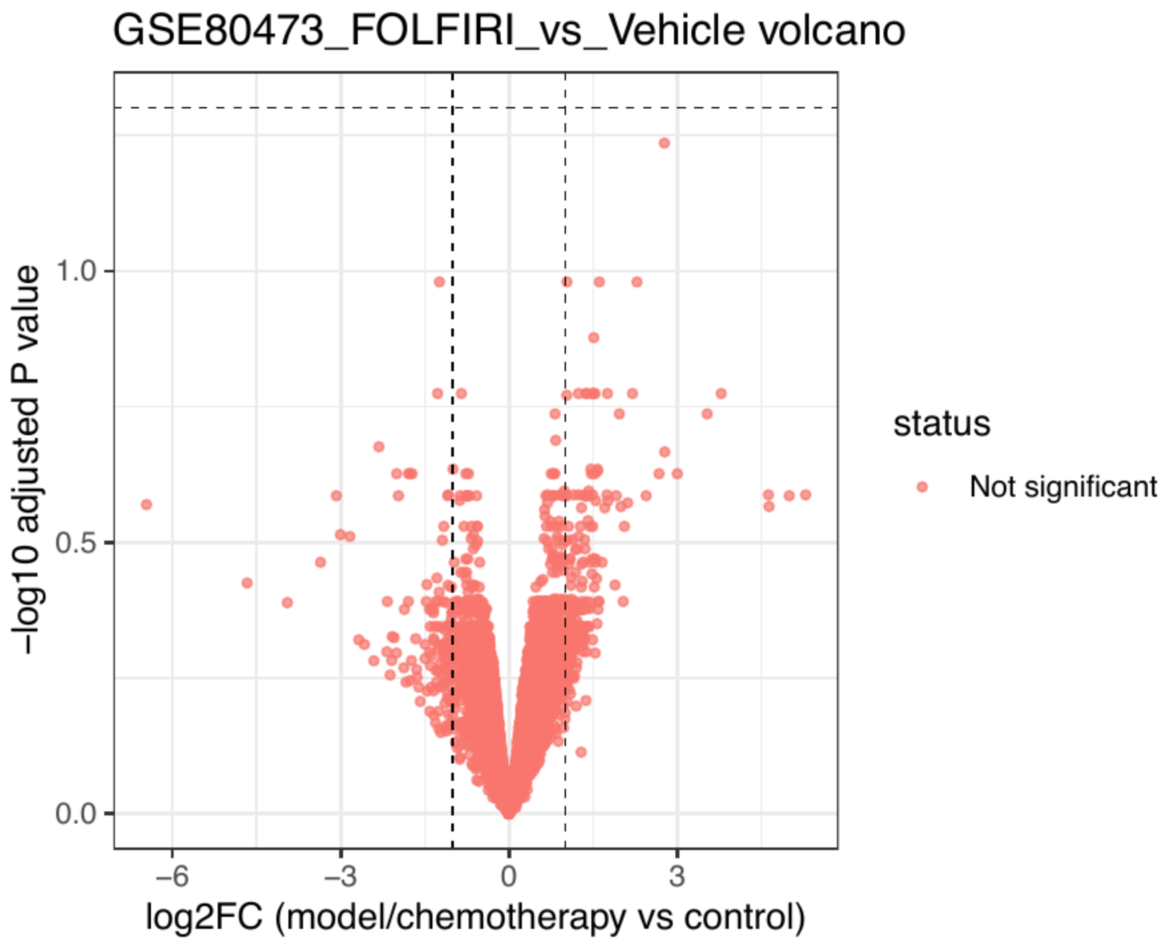


**Figure S52.** GSE80473 FOLFIRI versus Vehicle: differential expression volcano plot.

Original figure file: GSE80473_FOLFIRI_vs_Vehicle_volcano.pdf

## longitudinal food-intake:

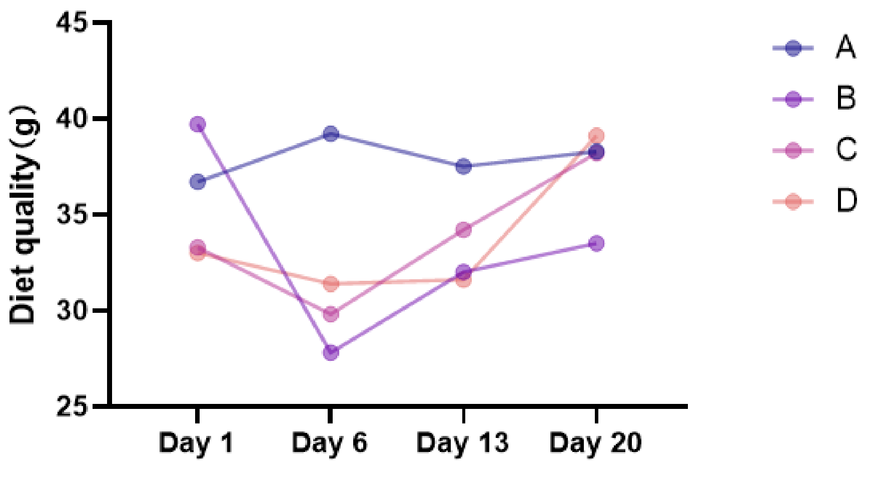


**Figure S53.** longitudinal food-intake
